# Transcriptome and metabolite analyses reveal the complex metabolic genes involved in volatile terpenoid biosynthesis in garden sage (*Salvia officinalis*)

**DOI:** 10.1038/s41598-017-15478-3

**Published:** 2017-11-22

**Authors:** Mohammed Ali, Penghui Li, Guangbiao She, Daofu Chen, Xiaochun Wan, Jian Zhao

**Affiliations:** 1 National Key Laboratory of Crop Genetic Improvement, Huazhong Agricultural University, Wuhan, 430070 China; 2 State Key Laboratory of Tea Plant Biology and Utilization, Anhui Agricultural University, Hefei, 230036 China; 3 Wuhan Doublehelix Biology Science and Technology Co. Ltd, Wuhan, 430070 China

## Abstract

A large number of terpenoid compounds have been extracted from different tissues of S. officinalis. However, the molecular genetic basis of terpene biosynthesis pathways is virtually unknown. In this study, approximately 6.6 Gb of raw data were generated from the transcriptome of S. officinalis leaves using Illumina HiSeq 2000 sequencing. After filtering and removing the adapter sequences from the raw data, the number of reads reached 21 million, comprising 98 million of high-quality nucleotide bases. 48,671 unigenes were assembled de novo and annotated for establishing a valid database for studying terpenoid biosynthesis. We identified 135 unigenes that are putatively involved in terpenoid metabolism, including 70 mevalonate and methyl-erythritol phosphate pathways, terpenoid backbone biosynthesis genes, and 65 terpene synthase genes. Moreover, five terpene synthase genes were studied for their functions in terpenoid biosynthesis by using transgenic tobacco; most transgenic tobacco plants expressing these terpene synthetic genes produced increased amounts of terpenoids compared with wild-type control. The combined data analyses from the transcriptome and metabolome provide new insights into our understanding of the complex metabolic genes in terpenoid-rich sage, and our study paves the way for the future metabolic engineering of the biosynthesis of useful terpene compounds in S. officinalis.

## Introduction

Garden sage (*Salvia officinalis* L.) belongs to the genus *Salvia*, which is one of the economically best-known genera due to its vast medicinal properties and rich aromatic oils. The genus *Salvia* (tribe Mentheae) is the largest of the Lamiaceae family, which comprises nearly 1,000 species. *Salvia* plants are widely distributed in three regions around the world but mainly exist in Central and South America (~500 species), West Asia (~200 species) and East Asia (~100 species), while the other *Salvia* species are spread throughout the world^1^. Most of these plants contain various medicinally active components used throughout history in folk medicine, e.g., *S*. *japonica*, *S*. *tuxtlensis*, *S*. *guaranitica*, *S*. *miltiorrhiza*, *S*. *chloroleuca*, *S*. *aureus*, *S*. *przewalskii*, *S*. *epidermidis*, *S*. *santolinifolia*, *S*. *hydrangea*, *S*. *tomentosa*, *S*. *isensis*, *S*. *lavandulifolia*, *S*. *glabrescens*, *S*. *nipponica*, *S*. *fruticosa*, *S*. *allagospadonopsis*, *S*. *macrochlamys* and *S*. *recognita*. Recently, *Salvia* species have become a valuable source for pharmaceutical research for identifying and discovering biologically active compounds^2^. Essential oils of *Salvia* species exhibit significant bioactivities, including antimutagenic, anticancer, antimicrobial, anti-inflammatory, choleretic, antioxidant and antimicrobial activities. *Salvia* essential oils contain more than 100 active compounds with pharmacological effects, and they can be categorized into monoterpenes, sesquiterpenes, diterpenes, and triterpenes^2^. During their biosynthesis, these terpenoids are sequentially built up from the isoprene unit (C5) building blocks, isopentyl diphosphates (IPP) and dimethylallyl diphosphate (DMADP). These components are condensed in a sequential manner by prenyltransferases, resulting in the formation of prenyl diphosphates, such as diphosphate (GPP), farnesyl pyrophosphate (FPP), and geranylgeranyl pyrophosphate (GGPP)^3^. These prenyl diphosphates are the immediate precursors for the biosynthesis of mono-, sesqui-, di- and tetraterpenes. Despite the scientific and medicinal interest in these terpenoids of *S*. *officinalis*, the genes that are involved in the biosynthesis of these compounds have not yet been fully identified or understood. Plant secondary metabolites have significant use in the food and pharmaceutical industries, such as in fine chemicals and cosmetics. The biosynthesis, regulation and metabolic engineering of useful secondary metabolites have been extensively studied^4^. In recent years, next-generation sequencing (NGS)-based RNA sequencing (RNA-Seq) has become a powerful tool for discovering genes that are involved in the biosynthesis of various secondary metabolite pathways in medicinal plants^5^. For example, the phenylpropanoid and terpenoid biosynthesis pathways in *Ocimum sanctum* and *Ocimum basilicum* ^6^, the biosynthesis of active ingredients in *Salvia miltiorrhiza* ^7^, the biosynthesis of carotenoids in *Momordica cochinchinensis* ^8^, the biosynthesis of cellulose and lignin in Chinese fir (*Cunninghaimia lanceolata*)^9^, and the biosynthesis of tea-specific compounds, i.e., catechins, caffeine and theanine pathways in tea (*Camellia sinensis*)^10^, have been explored using NGS. Characterization of plant terpene synthases (TPSs) is typically carried out by the production of the recombinant enzymes in *Escherichia coli*. This is often difficult due to enzyme solubility and codon usage issues. Furthermore, plant terpene synthases that are localized to the plastids, such as diterpene synthases, must be abridged in a more or less experimental approach to ameliorate expression^11,12^. Transgenic tobacco (*Nicotiana tabacum*) is very efficient and has been successfully used for the characterization of two diterpene genes in glandular trichomes: labdane and Z-abienol^13^. Here, we characterized genes that are involved in terpenoid biosynthesis in *S*. *officinalis* and determined their biological significance in *S*. *officinalis* for terpenoid production in various tissues. In this study, a transcriptome database was established for *S*. *officinalis* leaves using NGS technology to identify and to characterize genes that are related to the terpenoid biosynthesis pathway. The criteria used to achieve these objectives and to elucidate the complex metabolic pathways and genes for the understanding of terpenoid production in *S*. *officinalis* included the following: (i) transcriptome analysis of leaves using Illumina HiSeq 2000 sequencing; (ii) Gas Chromagraphy coupled Mass Spectrometry (GC-MS) analysis for three fresh plant parts (old leaves, young leaves, and stems); (iii) characterization of five terpene genes in transgenic *N*. *tabacum*; (iv) qRT-PCR of highly expressed genes that are involved in the biosynthesis of terpenoids; (v) and the combination of data from the transcriptome, qRT-PCR, and metabolome with GC-MS for revealing the functions of metabolic genes that are involved in the biosynthesis of valuable terpenoids.

## Results and Discussion

### Identification of essential oil components

**Figure 1:**
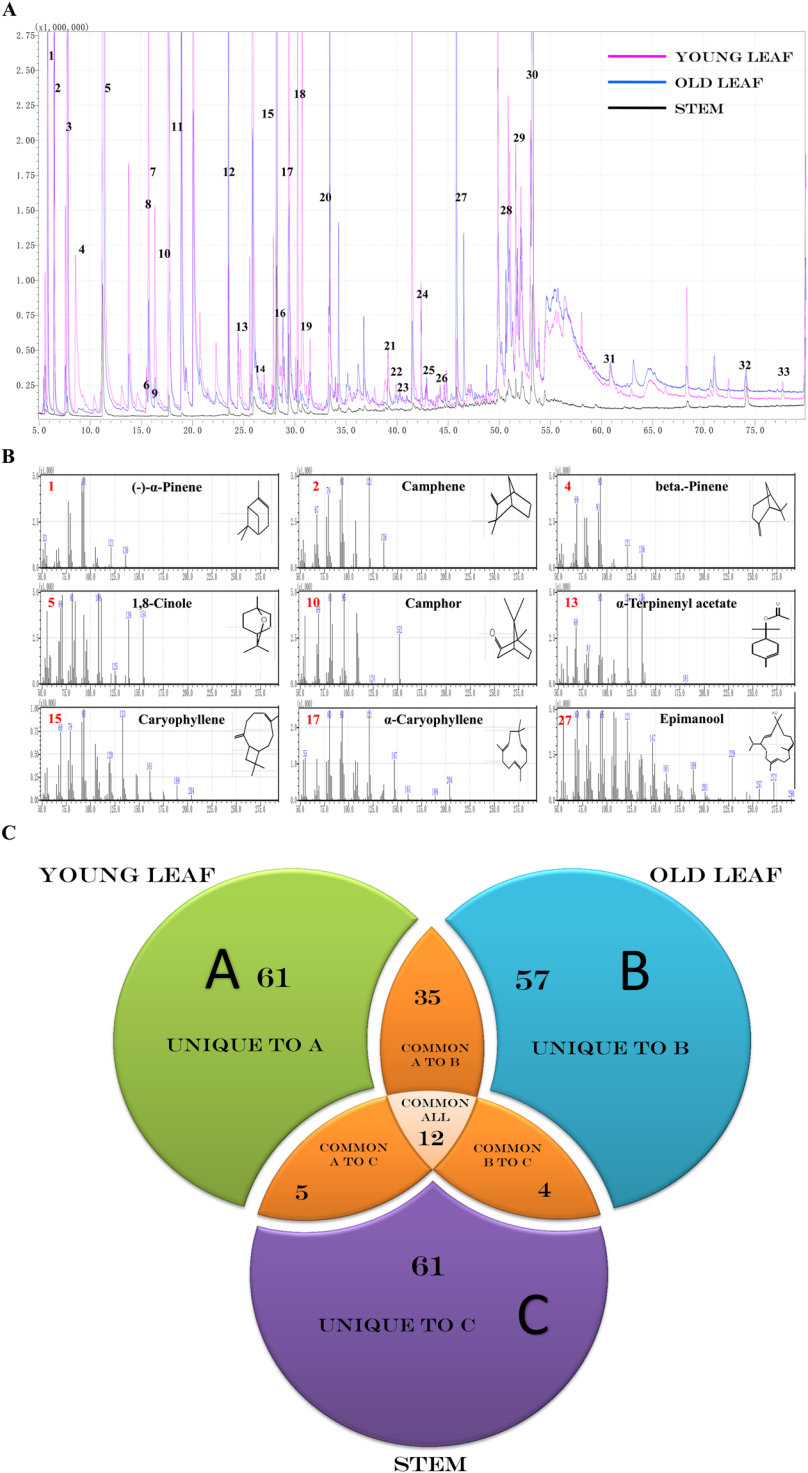
Typical GC-MS mass spectragraphs for terpenoids from young leaf, old leaf, and stem of *Salvia officinalis*. (**A**) GC-MS peaks of the essential oil extracts, (**B**) Mass spectrum of GC peaks with retention time for the major compound. (**C**) Three-Way-Venn-Diagram to show the number of unique and common compounds in the essential oil extracts from young leaf (**A**), old leaf (**B**), and stem (**C**) of *Salvia officinalis*.

For GC-MS analysis, 236 bioactive phytochemical compounds were identified using n-hexane extracts from three fresh aerial parts of *S*. *officinalis*. The numbers of obtained bioactive phytochemical compounds from young leaves, old leaves and stems were 113 (89.29%), 108 (91.54%) and 82 (85.27%), respectively. The results of the qualitative and quantitative analyses of all phytochemical compounds from the essential oils are reported in (Table 1 and Supplementary Table S1). The identified phytochemical compounds are listed based on the retention time, compound mass and percentage of peak area (Fig. 1A,B). In young leaves, the monoterpene compounds were shown as the main group (66.64%), followed by the group of sesquiterpene compounds (15.87%) and diterpene compounds (1.4%). In old leaves, the monoterpene compounds were observed to be the main group (52.7%), followed by the sesquiterpene group (15.01%) and the diterpene group (14.18%), and only one triterpene compound represented 0.16%. Sesquiterpenes form the main group of compounds (23%) found in the stems, followed by diterpenes (19.53%), monoterpenes (19.11%) and and one triterpene compound represented 0.02% (Supplementary Table S1). Moreover, the three hexane extracts from the different tissues for essential oils contained unique, common and major phytochemical compounds. For example, the essential oil extracts of young leaves (A) had 61 unique compounds, 35 common compounds shared with the essential oil extracts from old leaves, five common compounds shared with the essential oil extract from stems and 12 common compounds shared among all three plant parts. Furthermore, the old leaves (B) contained 57 unique compounds and four common compounds shared with the stems. On the other hand, the stems (C) contained 61 unique compounds (Fig. 1C). Regarding the major phytochemical compounds, 1,8-cineole (41.20%) was the major compound in the essential oil extracts from young leaves, followed by β-caryophyllene (9.01%), camphor (6.27%), β-pinene (6.23%), and α-terpinenyl acetate (4.23%), whereas the essential oil extracts of old leaves was characterized by 1,8-cineole (25.93%), followed by camphor (11.52%), sugiol (10.80%), β-caryophyllene (5.51%), and α-caryophyllene (3.72%). Sugiol was characterized as the major compound of stem extracts (15.89%), followed by 1,8-cineole (12.37%), β-caryophyllene (10.23%), α-caryophyllene (7.30%), and then isocaryophyllene oxide (3.24%) (Table 1). When comparing the composition of the three essential oil extracts of *S*. *officinalis*, we deduced that some common compounds exist at different levels within the parts of *S*. *officinalis* (Fig. 1A). Additionally, some of the compounds that have been found in *S*. *officinalis* were detected in other *Salvia* plant species (Table 1 and Supplementary Table S1)^14–16^. Therefore, we suggest that plant parts can have a major effect on the composition of their essential oils. From these and previous GC-MS data^17,18^, an important question has been raised: why do the monoterpene compounds of *S*. *officinalis* mostly accumulated in young leaves? This question was difficult to answer before conducting the present work because there was a lack of information at the genetic level regarding the terpenoid biosynthetic pathway and how these compounds are synthesized in *S*. *officinalis*.

**Table 1:** The major chemical compositions in the essential oils of *S*. *officinalis*.

N	Compound name	Retention time (min.)	Retention time index	Formula	Molecular Mass (g mol^−1^)	Terpene type	Young leaf	Old leaf	Stem	O.S.S
							% Peak area	% Peak area	% Peak area	
1	(−)-α-Pinene,	5.82	934	C10H16	136.23	Mono	**2.14**	**1.96**	**0.35**	S.L, S.A, S.F, S.C
2	Camphene-	6.48	941	C10H16	136.23	Mono	**0.54**	**2.17**	**0.15**	S.L, S.A, S.F, S.C
3	(±)-Sabinene	7.56	977	C10H16	136.23	Mono	**0.37**	**0.16**	—	S.L, S.A
4	beta.-Pinene	7.77	980	C10H16	136.23	Mono	**6.23**	**3.19**	**1.28**	S.L, S.A, S.F, S.C
5	1,8-cineole	11.29	1028	C10H18O	154.24	Mono	**41.2**	**25.93**	**12.37**	S.L, S.A, S.F
6	p-Menth-8-en-1-ol	15.47	1161	C10H18O	154.24	Mono	**0.07**	**0.15**	—	
7	δ-Thujone,	15.68	1103	C10H16O	152.23	Mono	**1.26**	**0.56**	—	
8	Thujone	16.31	1110	C10H16O	152.23	Mono	**0.51**	**0.17**	—	S.F
9	(−)-2-bornanone	17.69	1146	C10H18O	154.24	Mono	—	—	**1.62**	S.L, S.A, S.F, S.C
10	Camphor	17.70	1139	C10H16O	152.23	Mono	**6.27**	**11.52**	—	S.L, S.A, S.F
11	Terpinen-4-ol	19.33	1164	C10H18O	154.24	Mono	**0.06**	**0.16**	—	S.L, S.A, S.C
12	Bornyl acetate	23.54	1276	C17H24O4	196.28	Mono	**0.24**	**1.40**	**0.51**	S.F
13	α-Terpinenyl acetate	24.88	1341	C12H20O2	196.28	Mono	**4.23**	**1.26**	**1.52**	
14	α-Gurjunene,	27.76	1409	C15H24	204.35	Sesqui	**0.03**	**0.02**	—	S.L, S.A
15	Isocaryophyllene	28.20	1409	C15H24	204.35	Sesqui	**9.01**	**5.51**	**10.23**	S.F
16	Beta.-copaene	28.60	1455	C15H24	204.35	Sesqui	**0.02**	**0.01**	—	
17	α-caryophyllene	29.41	1442	C15H24	204.35	Sesqui	**2.22**	**3.72**	**7.30**	S.L, S.A
18	(−)-Germacrene D,	30.09	1482	C15H24	204.35	Sesqui	**0.06**	**0.14**	**1.29**	
19	Gamma.-Elemene	30.73	1433	C15H24	204.35	Sesqui	**0.8**	—	**0.36**	
20	Caryophyllene oxide	3.43	1573	C15H24	204.35	Sesqui	**0.48**	**2.13**	**3.24**	S.L, S.A, S.N
21	Geranyl-.alpha.-terpinene	39.88	2223	C20H32	272.46	Diter	**0.06**	**0.03**	—	S.L, S.A, S.F
22	Isoaromadendrene epoxide	40.79	1623	C15H24O	220.35	Sesqui	**0.4**	—	**0.08**	S.L, S.A
23	Beta.-ylangene	41.45	1421	C15H24	204.35	Sesqui	**0.96**	**0.36**	—	
24	Labda-8(20), Biformene	42.35	2004	C20H32	272.46	Diter	**0.17**	**0.25**	—	S.L, S.A
25	Kaur-16-en-18-yl acetate	42.79	1997	C22H34O2	330.50	Diter	**0.02**	**0.06**	—	
26	β-cis-Caryophyllene	43.82	1425	C15H24	204.35	Sesqui	**0.02**	**0.03**	—	S.F
27	Epimanool	45.80	2056	C20H34O	290.48	Diter	**2.07**	**2.10**	**167**	
28	Humulane-1,6-dien-3-ol	50.99	1619	C15H26O	222.36	Sesqui	**0.87**	**0.91**	—	
29	Ferruginol	52.11	2330	C20H30O	286.45	Diter	**0.51**	**0.63**	—	
30	Sugiol	53.07	2659.9	C20H28O2	300.43	Diter	**0.47**	**10.80**	**15.89**	
31	Totarol	60.84	2260	C20H30O	286.45	Diter	—	**0.13**	—	
32	Squalene	74.12	2817	C30H50	410.71	Diter	—	**0.16**	—	
33	2-methyloctacosane	79.90	2864	C29H60	408.78	Diter	**0.12**	**0.23**	**0.65**	
	Total						**89.29**	**91.54**	**85.27**	
Total Precentage of Monoterpenes							**66.64**	**52.7**	**19.11**	
Total Precentage of Sesquiterpenes							**15.87**	**15.01**	**23.12**	
Total Precentage of Triterpenes								**0.16**	**0.02**	
Total Precentage of Diterpenes							**1.4**	**14.18**	**19.53**	

Abbreviations: R.T: Retention Time, O.S.S: Other salvia species, SA: *Salvia acetabulosa*, S.L: *Salvia leriifolia*, S.F: *Salvia fruticosa*, S.N: *Salvia nemorosa*, S.C: *Salvia compressa*.

### Illumina sequencing and the *de novo* assembly of the *S*. *officinalis* leaf transcriptome

In the past few years, the Illumina sequencing platform has become a powerful method for analysing and discovering the genomes of non-model plants^19,20^. In this context, to generate transcriptome sequences, complementary DNA (cDNA) libraries were prepared from leaf tissues of *S*. *officinalis*, and cDNA was then sequenced using paired-end reads (PE) sequencing using an Illumina HiSeq 2000 platform. Previous reports involving Illumina sequencing reported that the use of PE sequencing showed significant improvement in the efficiency of *de novo* assembly and increased the depth of sequencing^9,21^. The cDNA sequencing generated 6.6 Gb of raw data from *S*. *officinalis* leaves. After filtering and removing the adapter sequences from the raw data, the number of reads was 21,487,871 (21.48 million), comprising of 98,521,170 high-quality nucleotide bases, with 95.90% Q20, 91.69% Q30 and 48.73% GC content. For further analysis, high-quality reads were selected, and the transcriptome was assembled using the Trinity program^22^, which produced 88,554 transcripts with an N50 length of 1,793 bp, an N90 length of 479 bp and a mean length of 1,113 bp. Moreover, 48,671 unigenes could be detected with an N50 length of 1,485 bp, an N90 length of 298 bp and a mean length of 813 bp. The distribution of the assembled transcript length ranged from 200 to >2,000 bases; the maximum number of transcripts (34,051 transcripts, 38.45%) ranged from 200 bp to 500 bp, followed by 22,529 transcripts (25.44%) ranging from 1,000 to 2,000 bp and then 17,658 transcripts (19.94%) ranging from 500 to 1,000 bp. On the contrary, the lowest number of transcripts (14,316 transcripts, 16.17%) was obtained for a size of more than 2,000 bp. By contrast, the assembled unigene lengths were distributed between 200 and >2,000 bp. The maximum number of unigenes (27,381 unigenes, 56.26%) ranged from 200 to 500 bp, followed by 8,576 unigenes (17.62%) ranging from 500 to 1,000 bp and then 8.068 unigenes (16.58%) ranging from 1000 to 2,000 bp. Finally, the lowest number of unigenes (4,646 unigenes, 9.54%) was obtained for a size of >2000 bp. The length distributions of the transcripts and unigenes are shown in Supplementary Table S2 and Fig. S1. Our results are in good agreement with those for *Boehmeria nivea*, *Medicago sativa*, *C*. *Longa*, *Centella asiatica* and *Apium graveolens*, in which the largest number of both transcript and unigene lengths were found to range between 75 and 500 bp^23,24^.

### Functional annotation and classification of assembled *S*. *officinalis* unigenes

**Figure 2:**
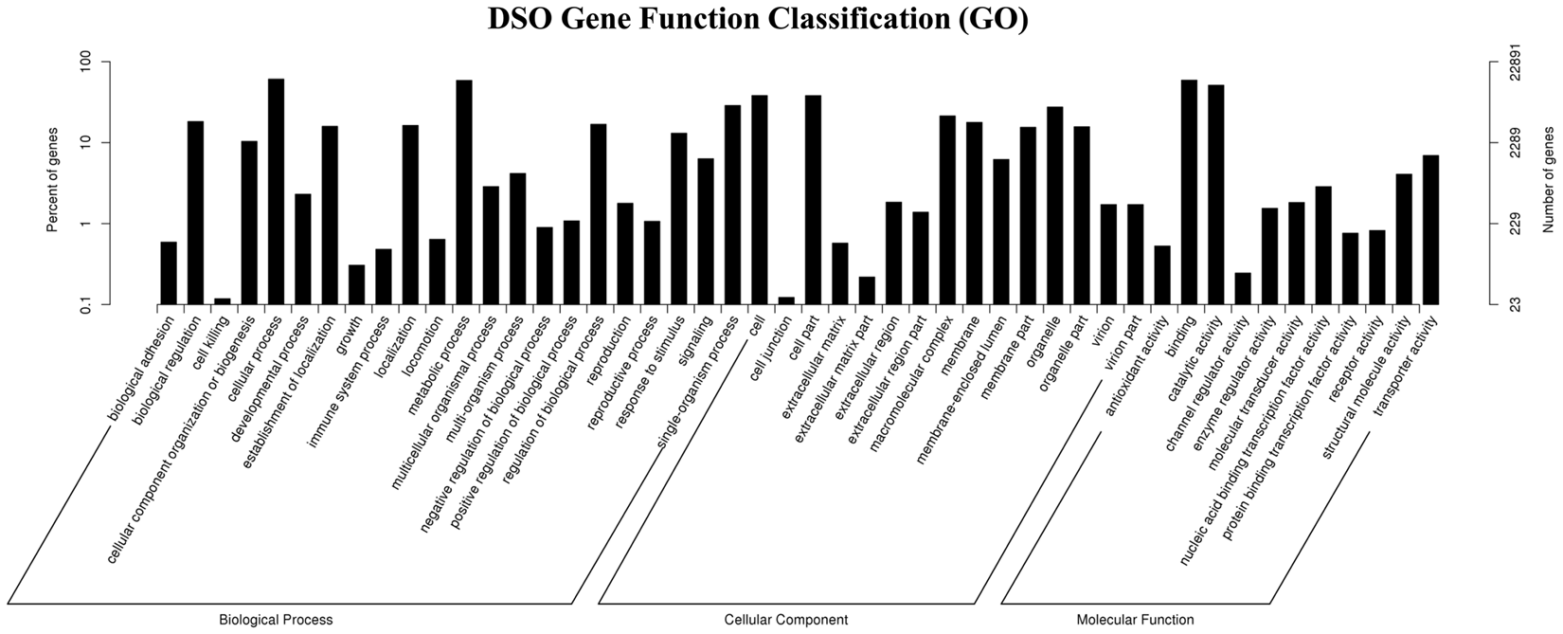
Functional annotation and classification of assembled unigenes from *S*. *officinalis*. Gene Ontology (GO) terms are summarized in three general sections of the biological process (BP), cellular component (CC) and molecular function (MF).

The total number of unigenes (48,671, 100% of all unigenes) was compared against the public dabases, including the NCBI non-redundant protein sequences (NR), the NCBI nucleotide sequences (NT), the Kyoto Encyclopedia of Genes and Genomes (KEGG), the KEGG orthology (KO), Swiss-Prot, the protein family annotation (PFAM), Gene Ontology (GO), and the euKaryotic Ortholog Groups database (KOG) annotation databases (Supplementary Table S3 and Fig. S2). The annotation percentage results in this research were higher than the annotation percentages in other non-model plant studies [58% in safflower (*Carthamus tinctorius*) and 58.01% in Chinese fir (*C*. *lanceolata*)]^9,25,26^. The international standardized gene functional annotation system (GO Annotation) provides a powerful way to recognize the functions and properties of sequences that have not been characterized for an organism^27^. The BLAST2 GO program was used to categorize the functions of these annotated unigenes, and a total of 22,891 unigenes (47.03% of all of the assembled unigenes) were mapped to at least one GO term. Based on sequence homology, the unigene sequences from *S*. *officinalis* were categorized into 48 functional groups under three general sections: 59,883 were assigned to the biological process (BP), 43,029 were assigned to the cellular component (CC) and 29,760 were assigned to the molecular function (MF) sections. As a result, cellular process (13,933) and metabolic process (13,423) were the most enriched GO terms in the biological process (BP) section. Regarding the CC section, the cell (8,737) and cell part (8,720) were the most enriched. Within the molecular function (MF) section, binding (13,539) and catalytic activity (11,726) were highly enriched (Fig. 2). These results revealed that the main Gene Ontology (GO) classifications in the annotated unigenes were responsible for metabolism and fundamental biological regulation. These results were similar to previous results with the *S*. *miltiorrhiza* transcriptome and with the transcriptomes of *O*. *sanctum* and *O*. *basilicum* (members of the same family), which have the highest percentages of metabolic process, cellular process, cell, cell part, binding and catalytic activity^28,29^. Moreover, these results are in agreement with previous studies on *de novo* transcriptome assembly in the tuberous root of sweet potato, *de novo* transcriptome sequencing from *R*. *sativus* and *de novo* characterization of roots from the Chinese medicinal plant *P*. *cuspidatum* ^26,29^. The lowest percentage of unigenes categories included channel regulator activity (56), cell junction (28) and cell killing (27). Therefore, the present work suggests that the enormous potential data that exist in the Gene Ontology (GO) classifications can be used to identify the new genes.

### KEGG analysis of *S*. *officinalis* transcriptomes

**Figure 3:**
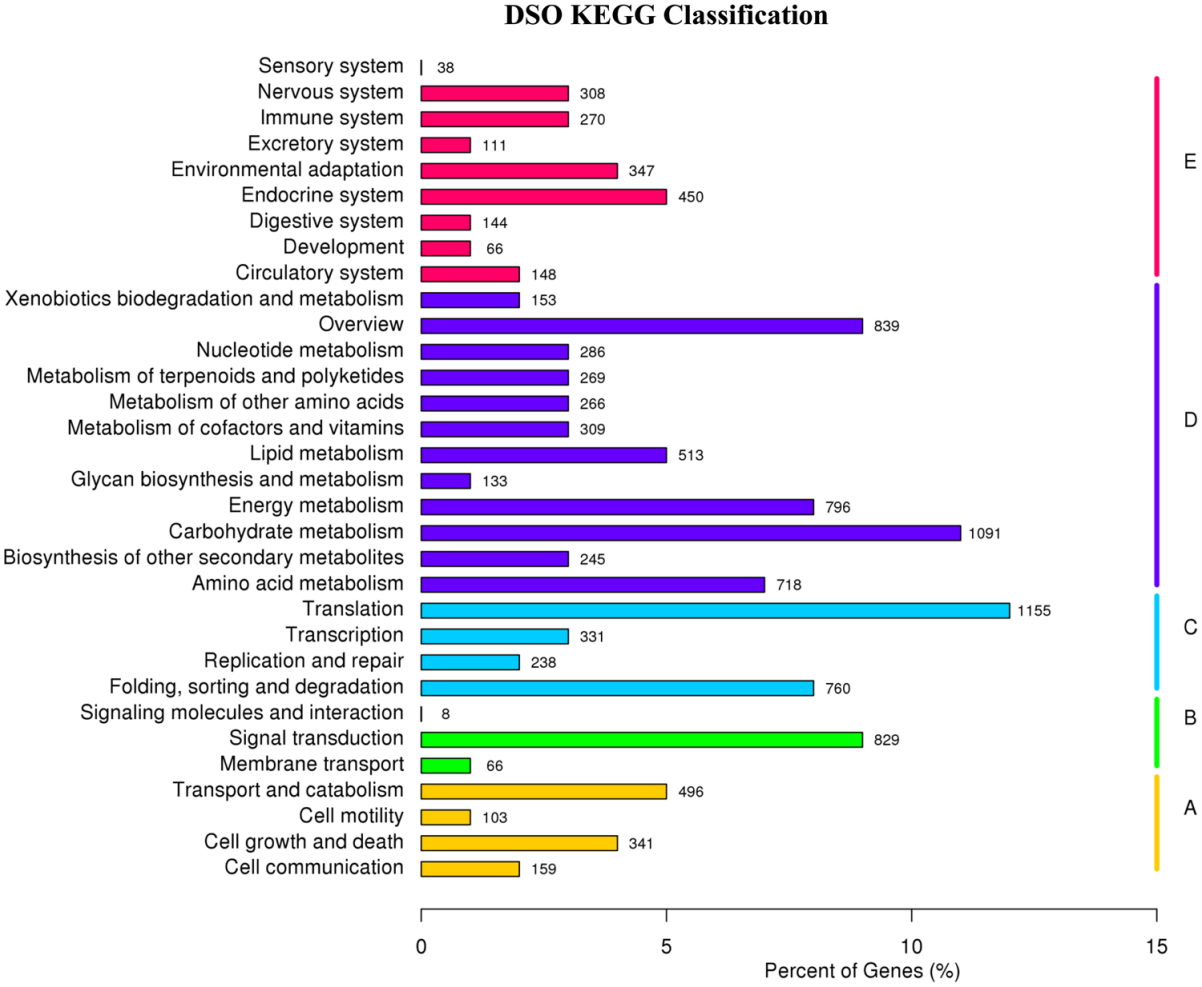
KEGG classified into five largest categories pathways includes cellular processes (**A**), environmental information processing (**B**), genetic information processing (**C**), metabolism (**D**) and organismal systems (**E**).

KEGG pathway database can facilitate the understanding of the functional annotations of enzymes and the biological functions of genes regarding their networks^7,30^. To identify active biological functional pathways in the leaf tissues of *S*. *officinalis*, all 48,671 unigene sequences were mapped in reference to the canonical pathways of KEGG, but 9,716 (19.96%) unigene sequences could be assigned to 267 KEGG pathways. Furthermore, all transcripts were classified into five larger pathway categories, including cellular processes, environmental information processing, genetic information processing, metabolism and organismal systems (Fig. 3). The highest number of transcripts from *S*. *officinalis* was assigned to the metabolism category, followed by genetic information processing, organismal systems, and cellular processes, whereas the lowest number of transcripts was related to the category of environmental information processing. Interestingly, 608 transcripts of *S*. *officinalis* were related to the biosynthesis of various secondary metabolite pathways, which were sorted into 27 subcategories, with phenylpropanoid biosynthesis (ko00940), terpenoid backbone biosynthesis (ko00900) and carotenoid biosynthesis (ko00906) representing the largest subcategories (Supplementary Table S4). These results were in agreement with previous results from the transcriptomes of *O*. *sanctum* and *O*. *basilicum*, which are members of the same family, and from *de novo* transcriptome sequencing from *R*. *sativus*, the transcriptome of which had the highest percentages of phenylpropanoid biosynthesis and terpenoid backbone biosynthesis^6,9^.

### Genes related to the biosynthesis of isoprenoids

**Figure 4:**
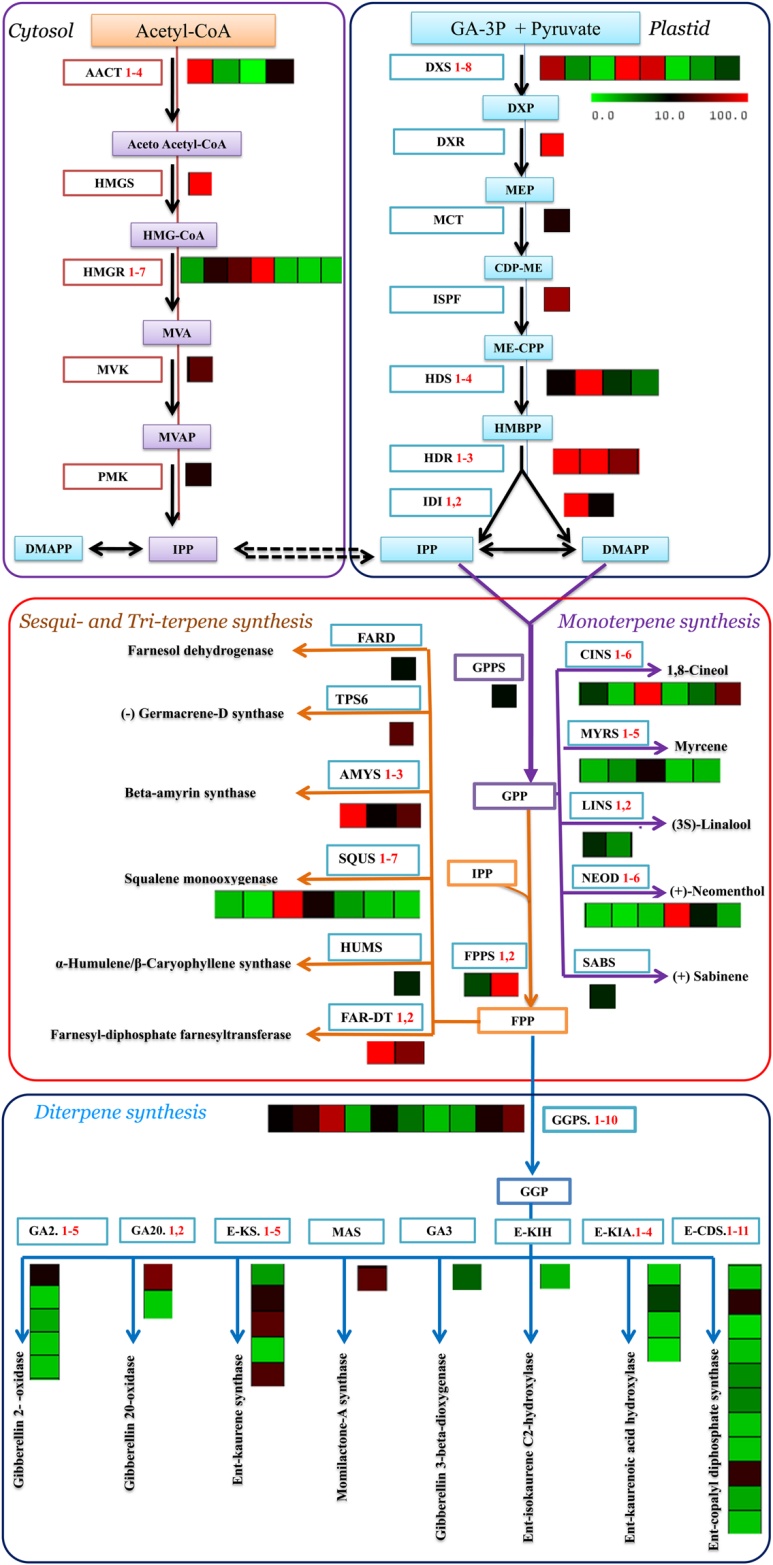
Representative terpenoid biosynthesis pathway with cognate heat maps for transcript levels of genes from transcriptome data with substrates and products, colored arrows connect substrates to their corresponding products. Green/red color-coded heat maps represent relative transcript levels of different terpenoid genes determined by Illumina HiSeq 2000 sequencing; red, upregulated; green, downregulated. Transcript levels data represent by FPKM: Fragments per Kilobase of transcripts per Million mapped fragments. MeV: MultiExperiment Viewer software was used to depict transcript levels. DXS: 1-deoxy-D-xylulose-5-phosphate synthase, DXR:1-deoxy-D-xylulose-5-phosphate reductoisomerase, MCT: 2-C-methyl-D-erythritol 4-phosphate cytidylyltransferase, ISPF: 2-C-methyl-D-erythritol 2,4-cyclodiphos-phate synthase, HDS:(E)-4-hydroxy-3-methylbut-2-enyl-diphosphate synthase, HDR: 4-hydroxy-3-methylbut-2-enyl diphosphate reductases, IDI: isopentenyl-diphosphate delta isomerase, AACT: acetyl-CoA C-acetyl transferase, HMGS: hydroxyl methyl glutaryl-CoA synthase, HMGR: hydroxymethyl glutaryl-CoA reductase (NADPH), MVK: mevalonate kinase, PMK: phospho-mevalonate kinase, GPPS: geranyl pyrophosphate synthase, FPPS: farnesyl pyrophosphate synthase, GGPS: geranylgeranyl pyrophosphate synthase, type II, CINO:1,8-cineole synthase, MYS: myrcene/ocimene synthase, LINA: (3S)-linalool synthase, NEOM:(+)-neomenthol dehydrogenase, SABI:(+)-sabinene synthase, TPS6:(−)-germacrene D synthase, AMS:beta-amyrin synthase, SEQ: Squalene monooxygenase, HUMS:α-humulene/β-caryophyllene synthase, GA2:gibberellin 2- -oxidase, GA20:gibberellin 20-oxidase, E-KS:ent-kaurene synthase, MAS:momilactone-A synthase, GA3:gibberellin 3-beta-dioxygenase, E-KIA: ent-isokaurene C2-hydroxylase, E-KIH:ent-kaurenoic acid hydroxylase, E-CDS: ent-copalyl diphosphate synthase.

Various types of terpenoids were found in the essential oil extracts of *S*. *officinalis*. The mixture contained mainly myrcene, (+)-neomenthol, 1,8-cineole, (3S)-linalool, α-humulene/β-caryophyllene, momilactone-A, gibberellin 3, gibberellin 2, ent-copalyl diphosphate, ent-kaurene, ent-kaurenoic acid, ent-isokaurene C2, gibberellin 20, and beta-amyrin. Precursor molecules for terpenoid biosynthesis are derived from the cytosolic mevalonate (MVA) and plastidial methyl-erythritol phosphate (MEP) pathways. Therefore, queries against the Lamiaceae family transcriptome libraries were applied to identify and to determine genes that encode enzymes involved in the different steps of the terpenoid biosynthesis pathway, such as Mevalonate diphosphate decarboxylase, Isopentenyl phosphate kinase, isopentenyl pyrophosphate isomerase for swithing IPP to DMAPP isomerase, GPS (geranyl pyrophosphate synthase), FPS (farnesyl pyrophosphate synthase) and GGPS (geranylgeranyl pyrophosphate synthase)^31,32^. Furthermore, we identified and estimated the expression levels of isoprenoid genes by using uniprot annotations against the transcriptome libraries (Table 2). From the annotation data analyses, we found many transcript genes related to isoprenoid biosynthesis from the MEP pathway with higher expression levels, including gene transcripts such as *SoDXS4*,*1*(1-deoxy-D-xylulose-5-phosphate synthase 4, 1), *SoDXR* (1-deoxy-D-xylulose-5-phosphate reductoisomerase), *SoMCT* (2-C-methyl-D-erythritol 4-phosphate cytidylyltransferase), *SoISPF* (2-C-methyl-D-erythritol 2,4-cyclodiphosphate synthase), *SoHDS2* ((E)-4-hydroxy-3-methylbut-2-enyl-diphosphate synthase 2), *SoHDR2*,3 (4-hydroxy-3-methylbut-2-enyl diphosphate reductase 2, 3) and *SoIDI1* (isopentenyl diphosphate isomerase1). Additionally, we obtained some gene transcripts that were related to isoprenoid biosynthesis from the MVA pathway with higher expression levels, such as *SoAACT1*, 4 (acetyl-CoA C-acetyltransferase 1, 4), *SoHMGS* (hydroxymethyl glutaryl-CoA synthase), *SoHMGR*4, 3, 2 (hydroxymethyl glutaryl-CoA reductase 4, 3, 2) *SoMVK* (mevalonate kinase) and *SoPMK* (phospho-mevalonate kinase). Moreover, the transcriptome dataset of *S*. *officinalis* presented other genes, such as *SoGPS*, *SoFPS2*, and *SoGGPSΙΙ10*, which are the immediate precursor of the mono-, sesqui-, and di-terpene biosynthesis pathway. The *So*GPS, *So*FPS2, and *So*GGPSΙΙ10 genes were highly abundant in leaves and had higher values of fragments per kilobase of transcripts per million mapped fragments (FPKM), which were 20.23, 281.11 and 49.23, respectively (Fig. 4 and Table 2). Our results were similar to previously obtained results from the transcriptomes of *O*. *sanctum* and *O*. *basilicum*, which are members of the same family and have a higher number of transcripts for the DXS and GPPS genes related to the terpenoid biosynthesis pathway^6^.

**Table 2:** Transcript abundance of MEP, MVA and other terpenoid backbone biosynthesis pathway genes as per the *S*. *officinalis* transcriptome data annotation.

Pathway	Gene name	Kegg Entry	Unigene ID	EC. No.	Gene length	Read in leaf	FPKM
MEP	*SoDXS1*	K01662	*SO|comp28301_c0*	2.2.1.7	2113	2308	72.96
	*SoDXS2*	K01662	*SO|comp3312_c0*	2.2.1.7	2536	153	4.36
	*SoDXS3*	K01662	*SO|comp528454_c0*	2.2.1.7	329	3	1.38
	*SoDXS4*	K01662	*SO|comp10248_c0*	2.2.1.7	2600	7406.34	186.68
	*SoDXS5*	K01662	*SO|comp28480_c0*	2.2.1.7	537	477.9	85.25
	*SoDXS6*	K01662	*SO|comp404816_c0*	2.2.1.7	417	5	1.38
	*SoDXS7*	K01662	*SO|comp4108_c0*	2.2.1.7	1360	75	3.91
	*SoDXS8*	K01662	*SO|comp15712_c0*	2.2.1.7	961	83	7.45
	*SoDXR*	K00099	*SO|comp10244_c0*	1.1.1.267	2044	4842.56	158.81
	*SoMCT*	K00991	*SO|comp17627_c0*	2.7.7.60	1180	373	22.99
	*SoISPF*	K01770	*SO|comp7621_c0*	4.6.1.12	1076	1173	80.86
	*SoHDS1*	K03526	*SO|comp23789_c0*	1.17.7.1	382	43	14.11
	*SoHDS2*	K03526	*SO|comp10199_c0*	1.17.7.1	4859	12460.35	161.86
	*SoHDS3*	K03526	*SO|comp23789_c1*	1.17.7.1	1511	161	7.89
	*SoHDS4*	K03526	*SO|comp8549_c0*	1.17.7.1	425	15	5.26
	*SoHDR1*	K03527	*SO|comp6889_c0*	1.17.1.2	460	599	138.19
	*SoHDR2*	K03527	*SO|comp26756_c1*	1.17.1.2	1992	22309.1	755.85
	*SoHDR3*	K03527	*SO|comp24712_c0*	1.17.1.2	1515	1249.27	57.42
	*SoIDI1*	K01823	*SO|comp26922_c0*	5.3.3.2	1512	6989.53	322
	*SoIDI2*	K01823	*SO|comp20600_c0*	5.3.3.2	845	148	13.84
MVA	*SoAACT1*	K00626	*SO|comp27297_c0*	2.3.1.9	1682	4550	185.61
	*SoAACT2*	K00626	*SO|comp1036893_c0*	2.3.1.9	226	2	3.25
	*SoAACT3*	K00626	*SO|comp1105580_c0*	2.3.1.9	206	0	0
	*SoAACT4*	K00626	*SO|comp15111_c0*	2.3.1.9	1820	510.61	19.06
	*SoHMGS*	K01641	*SO|comp10117_c0*	2.3.3.10	1878	6188.8	223.01
	*SoHMGR1*	K00021	*SO|comp63356_c0*	1.1.1.34	1323	69	3.71
	*SoHMGR2*	K00021	*SO|comp26128_c0*	1.1.1.34	2000	596	25.17
	*SoHMGR3*	K00021	*SO|comp17290_c0*	1.1.1.34	2973	2018.51	44.04
	*SoHMGR4*	K00021	*SO|comp27097_c0*	1.1.1.34	2068	6819.56	220.78
	*SoHMGR5*	K00021	*SO|comp574496_c0*	1.1.1.34	244	2	2.34
	*SoHMGR6*	K00021	*SO|comp12556_c0*	1.1.1.34	262	2	1.79
	*SoHMGR7*	K00021	*SO|comp8357_c0*	1.1.1.34	286	3	2.02
	*SoMVK*	K00869	*SO|comp26218_c0*	2.7.1.36	1670	671.21	42.87
	*SoPMK*	K00938	*SO|comp23601_c0*	2.7.4.2	2023	656.67	23.66
Monoterpene	*SoGPS*	K14066	*SO|comp20551_c0*	2.5.1.1	1329	511.75	20.23
Sesqui and Triterpene	*SoFPPS1*	K00787	*SO|comp15527_c0*	2.5.1.10	1426	101	7.1
	*SoFPPS2*	K00787	*SO|comp10352_c0*	2.5.1.10	1540	6232.1	281.11
Diterpene	*SoGGPSΙΙ1*	K13789	*SO|comp10800_c1*	2.5.1.29	426	47	12.46
	*SoGGPSΙΙ2*	K13789	*SO|comp25415_c0*	2.5.1.29	1441	548.03	26.69
	*SoGGPSΙΙ3*	K13789	*SO|comp8246_c0*	2.5.1.29	1466	1556	74.28
	*SoGGPSΙΙ4*	K13789	*SO|comp116899_c0*	2.5.1.29	251	3	3.14
	*SoGGPSΙΙ5*	K13789	*SO|comp10800_c0*	2.5.1.29	827	149	14.33
	*SoGGPSΙΙ6*	K13789	*SO|comp60755_c0*	2.5.1.29	1035	77	5.57
	*SoGGPSΙΙ7*	K13789	*SO|comp107254_c0*	2.5.1.29	793	25	2.54
	*SoGGPSΙΙ8*	K13789	*SO|comp394168_c0*	2.5.1.29	243	3	3.56
	*SoGGPSΙΙ9*	K13789	*SO|comp24966_c0*	2.5.1.29	1367	392	20.3
	*SoGGPSΙΙ10*	K13789	*SO|comp28724_c0*	2.5.1.29	1515	1071	49.23
Other Terpenoid Backbone Biosynthesis	*SoFLDH*	K15891	*SO|comp24181_c1*	1.1.1.216	1829	436	16.18
	*SoFOLK1*	K15892	*SO|comp22857_c1*	2.7.1.	789	89	9.11
	*SoFOLK2*	K15892	*SO|comp31365_c0*	2.7.1.	1300	605.78	33.27
	*SoFOLK3*	K15892	*SO|comp825225_c0*	2.7.1.	205	2	5.34
	*SoFOLK4*	K15892	*SO|comp22857_c0*	2.7.1.	273	27	21.03
	*SoPCYOX1*	K05906	*SO|comp17568_c0*	1.8.3.5, 1.8.3.6	2275	717	21.78
	*SoSTE24-1*	K06013	*SO|comp520699_c0*	3.4.24.84	326	4	1.88
	*SoSTE24-2*	K06013	*SO|comp8969_c0*	3.4.24.84	1628	659.79	27.93
	*SoCHLP1*	K10960	*SO|comp12058_c0*	1.3.1.83	1475	92	4.49
	*SoCHLP2*	K10960	*SO|comp18613_c0*	1.3.1.83	2153	20001.75	619.4
	*SoFACE2*	K08658	*SO|comp23619_c0*	3.4.22.-	1334	215	13.49
	*SoPCME1*	K15889	*SO|comp7802_c0*	3.1.1.-	1819	1899.94	71.36
	*SoPCME2*	K15889	*SO|comp1774_c0*	3.1.1.-	332	3	1.35
	*SoPCME3*	K15889	*SO|comp321627_c0*	3.1.1.-	531	8	1.45
	*SoFNTB*	K05954	*SO|comp25369_c0*	2.5.1.58	1536	283.6	12.95
	*SoFNTA*	K05955	*SO|comp21306_c0*	2.5.1.58	1260	297	18.19
	*SoSPS*	K05356	*SO|comp10273_c0*	2.5.1.84, 2.5.1.85	1915	6102.29	215.16
	*SoDHDDS1*	K11778	*SO |comp24971_c0*	2.5.1.87	1475	6128.72	362.2
	*SoDHDDS2*	K11778	*SO |comp24827_c0*	2.5.1.87	1631	135.49	6.75
	*SoDHDDS3*	K11778	*SO |comp193146_c0*	2.5.1.87	368	5	1.77
	*SoDHDDS4*	K11778	*SO |comp26151_c1*	2.5.1.87	1619	1109.77	74.03
	*SoDHDDS5*	K11778	*SO |comp16046_c0*	2.5.1.87	1617	3689.56	179.12
	*SoICMT1*	K00587	*SO|comp11697_c0*	2.1.1.100	677	27	3.41

Abbreviations: FPKM: Fragments per Kilobase of transcripts per Million mapped fragments, *SoDXS*: 1-deoxy-D-xylulose-5-phosphate synthase, *SoDXR*:1-deoxy-D-xylulose-5-phosphate reductoi*So*merase, *SoMCT*: 2-C-methyl-D-erythritol 4-phosphate cytidylyltransferase, *SoISPF*: 2-C-methyl-D-erythritol 2,4-cyclodiphosphate synthase, *SoHDS*:(E)-4-hydroxy-3-methylbut-2-enyl-diphosphate synthase, *SoHDR*: 4-hydroxy-3-methylbut-2-enyl diphosphate reductases, *SoIDI*: i*So*pentenyl-diphosphate delta i*So*merase, *SoAACT*: acetyl-CoA C-acetyl transferase, *SoHMGS*: hydroxyl methyl glutaryl-CoA synthase, *SoHMGR*: hydroxyl methyl glutaryl-CoA reductase (NADPH), *SoMVK*: mevalonate kinase, *SoPMK*: phospho-mevalonate kinase, *SoGPS*: geranyl pyrophosphate synthase, *SoFPPS*: farnesyl pyrophosphate synthase, *SoGGPS*: geranylgeranyl pyrophosphate synthase, type II, *SoFLDH*: farne*So*l dehydrogenase, *SoFOLK*: farne*So*l kinase, *SoPCYOX1*: prenylcysteine oxidases/farnesyl cysteine lyase, *SoSTE24*: STE24 endopeptidases, *SoCHLP*: geranylgeranyl reductase, *SoFACE*: prenyl-protein peptidases, *SoPCME*: prenylcysteine alpha-carboxyl methylesterase, *SoFNTB*: protein farnesyltransferase subunit beta, *SoFNTA*: protein farnesyltransferase/geranylgeranyltransferase type-1 subunit alpha, *SoSPS*: all-trans-nonaprenyl-diphosphate synthase, *SoDHDDS*: Di trans, polycis-polyprenyl diphosphate synthase, *So ICMT*: protein-S-i*So*prenylcysteine O-methyltransferase.

### Genes related to terpene synthases

Plants produce various terpenoid compounds with highly diverse structures. These compounds play an important role and function in the interactions with environmental factors and in fundamental biological processes^32,33^. Multiple terpenoids are synthesized in plants by the expression of many TPS genes. Moreover, some TPSs have the ability to catalyse the production of multiple products. Thus, the *TPS* gene family was classified according to phylogenetic relationships into eight subfamilies (TPS a, b, c, d, e/f, g, and h), which comprise mono-, sesqui-, di- and triterpene synthases^34^. Therefore, the annotation of transcriptome data from *S*. *officinalis* against the Lamiaceae family and Arabidopsis revealed many terpene synthases involved in the terpenoid biosynthesis pathway, e.g., myrcene, (+)-neomenthol, 1,8-cineole, (3S)-linalool, α-humulene/β-caryophyllene, momilactone-A, gibberellin 3, gibberellin 2, ent-copalyl diphosphate, ent-kaurene, ent-kaurenoic acid, ent-isokaurene C2, gibberellin 20, beta-amyrin and squalene. From the dataset, 65 TPS unigenes were identified and determined based on sequence similarities with a TPS sequence in the canonical annotation reference database. Twenty unigenes were annotated as being involved in monoterpene biosynthesis, including myrcene/ocimene synthase, (+)-neomenthol dehydrogenase, 1,8-cineole synthase, (+)-sabinene synthase and (3S)-linalool synthase, and three other unigenes were annotated as being involved in sesquiterpene biosynthesis, including α-humulene/β-caryophyllene synthase and (−)-germacrene D synthase. Additionally, 29 unigenes were annotated as being involved in diterpene biosynthesis, including momilactone-A synthase, gibberellin 3-beta-dioxygenase, gibberellin 2-oxidase, ent-copalyl diphosphate synthase, ent-kaurene synthase, ent-kaurenoic acid hydroxylase, ent-isokaurene C2-hydroxylase and gibberellin 20-oxidase. Finally, 12 unigenes were annotated as being involved in triterpene biosynthesis, including beta-amyrin synthase, squalene monooxygenase, and farnesyl-diphosphate, but some of these 12 genes showed high abundance in leaves and higher FPKM values (Fig. 4 and Table 3). The previous compounds have significant pharmacological activities, such as anticancer, anti-HIV, antiviral, anti-inflammatory and antibacterial activities. Sesquiterpenoids are similar to triterpenoids as both share the same origin and originate from farnesyl diphosphate (FDP). Triterpenoid compounds originate from the conversion of FDP into squalene by squalene synthase (SQS) and then to (S)-2,3-epoxysqualene by squalene monooxygenase (SQE)]. Subsequently, (S)-2,3-epoxysqualene is converted to beta-amyrin and camelliol C in the presence of multifunctional (S)-2,3-epoxysqualene cyclase via beta-amyrin synthase and camelliol C synthase, respectively. Similar reports about triterpenoid biosynthesis from (S)-2,3-epoxysqualene cyclases are available for *O*. *basilicum* and *Catharanthus roseus* ^35,36^.

**Table 3:** Transcript abundance of TPS genes as per the *S*. *officinalis* transcriptome.

Terpene synthase	Kegg Entry	Unigene ID	Annotation	Length (bp)	EC. No.	Read in leaf	FPKM
Monoterpene	K12467	*So|comp422551_c0*	myrcene/ocimene synthase	203	4.2.3.15	1	2.83
	K12467	*So |comp431748_c0*	myrcene/ocimene synthase	214	4.2.3.15	2	4.24
	K12467	*So |comp11163_c0*	myrcene/ocimene synthase	387	4.2.3.15	53	16.93
	K12467	*So |comp191_c0*	myrcene/ocimene synthase	280	4.2.3.15	3	2.15
	K12467	*So |comp189977_c0*	myrcene/ocimene synthase	458	4.2.3.15	12	2.79
	K15095	*So |comp8961_c0*	(+)-neomenthol dehydrogenase	256	1.1.1.208	2	1.95
	K15095	*So |comp210403_c0*	(+)-neomenthol dehydrogenase	433	1.1.1.208	5	1.29
	K15095	*So |comp972_c0*	(+)-neomenthol dehydrogenase	327	1.1.1.208	5	2.33
	K15095	*So |comp26078_c1*	(+)-neomenthol dehydrogenase	566	1.1.1.208	55	9.04
	K15095	*So |comp12329_c0*	(+)-neomentholdehydrogenase	447	1.1.1.208	14	3.4
	K15095	*So |comp10962_c0*	(+)-neomenthol dehydrogenase	1072	1.1.1.208	2170.28	136.67
	K07385	*So |comp5570_c0*	1,8-cineole synthase	354	4.2.3.108	20	7.73
	K07385	*So |comp184887_c0*	1,8-cineole synthase	402	4.2.3.108	8	2.37
	K07385	*So |comp144107_c0*	1,8-cineole synthase	295	4.2.3.108	4	2.46
	K07385	*So |comp53392_c0*	1,8-cineole synthase	453	4.2.3.108	24	5.69
	K07385	*So |comp26990_c0*	1,8-cineole synthase	2092	4.2.3.108	10511.52	335.99
	K07385	*So |comp14705_c0*	1,8-cineole synthase	1978	4.2.3.108	1339.74	49.23
	K07385	*So |comp18462_c0*	(+)-sabinene synthase	1182	4.2.3.108	93	7.5
	K15086	*So |comp15872_c0*	(3S)-linalool synthase-1	1763	4.2.3.25	95	8.28
	K15086	*So |comp6814_c0*	(3S)-linalool synthase-2	1789	4.2.3.25	116	4.41
Sesquiterpene	K14184	*So |comp101158_c0*	α-humulene/β-caryophyllene synthase	284	4.2.3.57	10	6.87
	K15803	*So |comp26367_c0*	(−)-germacrene D synthase	1965	4.2.3.75	777.46	39.5
Diterpene	K13070	*So |comp21612_c0*	momilactone-A synthase	1348	1.1.1.295	309	18.14
	K04124	*So |comp14297_c0*	gibberellin 3-beta-dioxygenase	1149	1.14.11.15	97	6.17
	K04125	*So |comp19885_c0*	gibberellin 2- oxidase	1257	1.14.11.13	319	18.23
	K04125	*So |comp436332_c0*	gibberellin 2- oxidase	217	1.14.11.13	1	1.97
	K04125	*So |comp324252_c0*	gibberellin 2- oxidase	226	1.14.11.13	2	3.25
	K04125	*So |comp747555_c0*	gibberellin 2- oxidase	212	1.14.11.13	1	2.22
	K04125	*So |comp5948_c0*	gibberellin 2- oxidase	274	1.14.11.13	3	2.31
	K04120	*So |comp2895_c0*	ent-copalyl diphosphate synthase	392	5.5.1.13	7	2.18
	K04120	*So |comp274743_c0*	ent-copalyl diphosphate synthase	356	5.5.1.13	6	2.29
	K04120	*So |comp246076_c0*	ent-copalyl diphosphate synthase	488	5.5.1.13	7	1.46
	K04120	*So |comp132266_c0*	ent-copalyl diphosphate synthase	656	5.5.1.13	18	2.38
	K04120	*So |comp15163_c0*	ent-copalyl diphosphate synthase	486	5.5.1.13	21	4.45
	K04120	*So |comp350819_c0*	ent-copalyl diphosphate synthase	209	5.5.1.13	2	4.8
	K04120	*So |comp112536_c0*	ent-copalyl diphosphate synthase	491	5.5.1.13	11	2.27
	K04120	*So |comp689_c0*	ent-copalyl diphosphate synthase	544	5.5.1.13	13	2.27
	K04120	*So |comp6575_c0*	ent-copalyl diphosphate synthase	2713	5.5.1.13	1174	28.26
	K04120	*So |comp2827_c0*	ent-copalyl diphosphate synthase	246	5.5.1.13	3	3.39
	K04120	*So |comp23218_c0*	ent-copalyl diphosphate synthase	3679	5.5.1.13	1267.32	25.28
	K04121	*So |comp20544_c0*	ent-kaurene synthase	1775	4.2.3.19	992	38.08
	K04121	*So |comp21378_c0*	ent-kaurene synthase	3023	4.2.3.19	760.01	24.46
	K04121	*So |comp22699_c0*	ent-kaurene synthase	2049	4.2.3.19	968	41.49
	K04121	*So |comp186871_c0*	ent-kaurene synthase	674	4.2.3.19	14	1.78
	K04121	*So |comp4251_c0*	ent-kaurene synthase	510	4.2.3.19	20	3.88
	K04123	*So |comp15654_c0*	ent-kaurenoic acid hydroxylase	1954	1.14.13.79	212	7.43
	K04123	*So |comp338203_c0*	ent-kaurenoic acid hydroxylase	257	1.14.13.79	2	1.92
	K04123	*So |comp404902_c0*	ent-kaurenoic acid hydroxylase	324	1.14.13.79	4	1.91
	K04123	*So |comp353538_c0*	ent-kaurenoic acid hydroxylase	288	1.14.13.79	2	1.32
	K16083	*So |comp1777_c0*	ent-isokaurene C2-hydroxylase	231	4.2.3.103	2	2.94
	K05282	*So |comp23119_c0*	gibberellin 20-oxidase	1354	1.14.11.12	997.41	52.75
	K05282	*So |comp210082_c0*	gibberellin 20-oxidase	466	1.14.11.12	9	2.03
Triterpene	K15813	*So |comp27006_c0*	beta-amyrin synthase	3060	5.4.99.39	14004.17	296.25
	K15822	*So |comp17362_c0*	beta-amyrin synthase	2661	5.4.99.39	1712.24	42.09
	K15813	*So |comp16071_c0*	beta-amyrin synthase	282	5.4.99.39	13	13.23
	K00511	*So |comp26984_c0*	Squalene monooxygenase	2092	1.14.13.132	8377.74	267.79
	K00511	*So |comp24504_c0*	Squalene monooxygenase	2141	1.14.13.132	600	18.69
	K00511	*So |comp1139_c0*	Squalene monooxygenase	326	1.14.13.132	3	1.41
	K00511	*So |comp98442_c0*	Squalene monooxygenase	263	1.14.13.132	3	2.65
	K00511	*So |comp14919_c0*	Squalene monooxygenase	492	1.14.13.132	14	3.81
	K00511	*So |comp11693_c0*	Squalene monooxygenase	211	1.14.13.132	1	2.28
	K00511	*So |comp12366_c0*	Squalene monooxygenase	594	1.14.13.132	12	1.83
	K00801	*So |comp26757_c1*	farnesyl-diphosphate farnesyltransferase	1934	2.5.1.21	3936.99	139.91
	K00801	*So |comp29648_c0*	farnesyl-diphosphate farnesyltransferase	443	2.5.1.21	228	56.24

### SSR discovery and analysis

The Illumina HiSeq 2000 system offers the opportunity to analyse molecular markers such as simple sequence repeats (SSRs) that are related to terpenoid pathway genes. SSR molecular markers have proven to be a powerful method for understanding genetic variation. Moreover, polymorphic SSR markers are very important for the investigation of related comparative genomics, genetic diversity, evolution, linkage mapping, gene-based association studies, and relatedness. Even though SNP markers have become promising, especially for studying complex genetic traits and high-throughput mapping, SSRs provide many advantages compared with other marker systems. Hence, SSRs have become the preferable codominant molecular marker for the construction of linkage maps^37^. Therefore, the development of novel SSR molecular markers for *S*. *officinalis* plants could be a valuable tool for breeding studies and genetic applications. Therefore, SSR markers were identified from transcriptome sequencing data using MISA (MIcroSAtellite) (http://pgrc.ipkgatersle-ben.de/misa/misa.html). Of the 48,671 transcripts of *S*. *officinalis*, 7,439 transcripts were observed to have SSRs (Supplementary Table S5). The total number of SSR-containing sequences in *S*. *officinalis* was 9,149 following stringent selection criteria used to identify these SSRs. The analysis data showed that dinucleotide repeats were the most abundant motif type in *S*. *officinalis* (4,295; 44.132%), followed by mononucleotide (2,348; 24.13%), trinucleotide (2,317; 23.81%), tetranucleotide (116; 1.191%), and hexanucleotide (39; 0.4%) types, while the pentanucleotide type was the least abundant motif (34; 0.35%) (Supplementary Tables S6 and S7 and Fig. S3). Except for the absence of mononucleotides, these results were similar to the previous results obtained from the transcriptomes of *O*. *sanctum* and *O*. *basilicum* (members of the same family), which have dinucleotide repeats as the most abundant motif type, followed by tri-, tetra-, hexa- and penta nucleotide types as the least abundant motif ^6^. After analysing the data from mono- to hexanucleotide motifs to obtain the number of repeat units, we found that the highest repeat unit of potential SSRs was 6, which accounted for 1,999 SSRs (21.86%), followed by 10 SSRs (1,490; 16.30%), 5 (1,411; 15.43%), and 7 (1,301; 14.23%), and the smallest repeat unit of potential SSRs was ≥24 (7; 0.08) (Supplementary Table S7). The AG/CT dinucleotide repeat was the most prevalent motif detected in all SSRs (2,999; 30.81%) followed by A/T as a mononucleotide repeat (2,272; 23.34%). By contrast, the least abundant motif in all SSRs (4; 0.041%) was detected in (AAAAC/GTTTT/AAAAG/CTTTT/AAAAT/ATTTT/AAACC/GGTTT) as pentanucleotide repeat and in (AAAATG/ATTTTC/AAATAG/ATTTCT/AAATTC/AATTTG/AACAAT/ATTGTT) as hexanucleotide repeat. Finally, several SSR motifs were associated with many unique sequences that encode enzymes (e.g. SoDXS4, SoDXS5, SoHDR2, SoHMGS, SoHMGR3, SoFLDH, SoPCYOX1, SoFNTA, SoDHDDS1, SoDHDDS5, momilactone-A synthase, SoGGPSΙΙ7, SoGGPSΙΙ10, ent-copalyl diphosphate synthase, ent-kaurenoic acid hydroxylase, beta-amyrin synthase and squalene monooxygenase) involved in terpenoid biosynthesis (Supplementary Table S8).

### Validation of the gene expression patterns by quantitative RT-PCR

**Figure 5:**
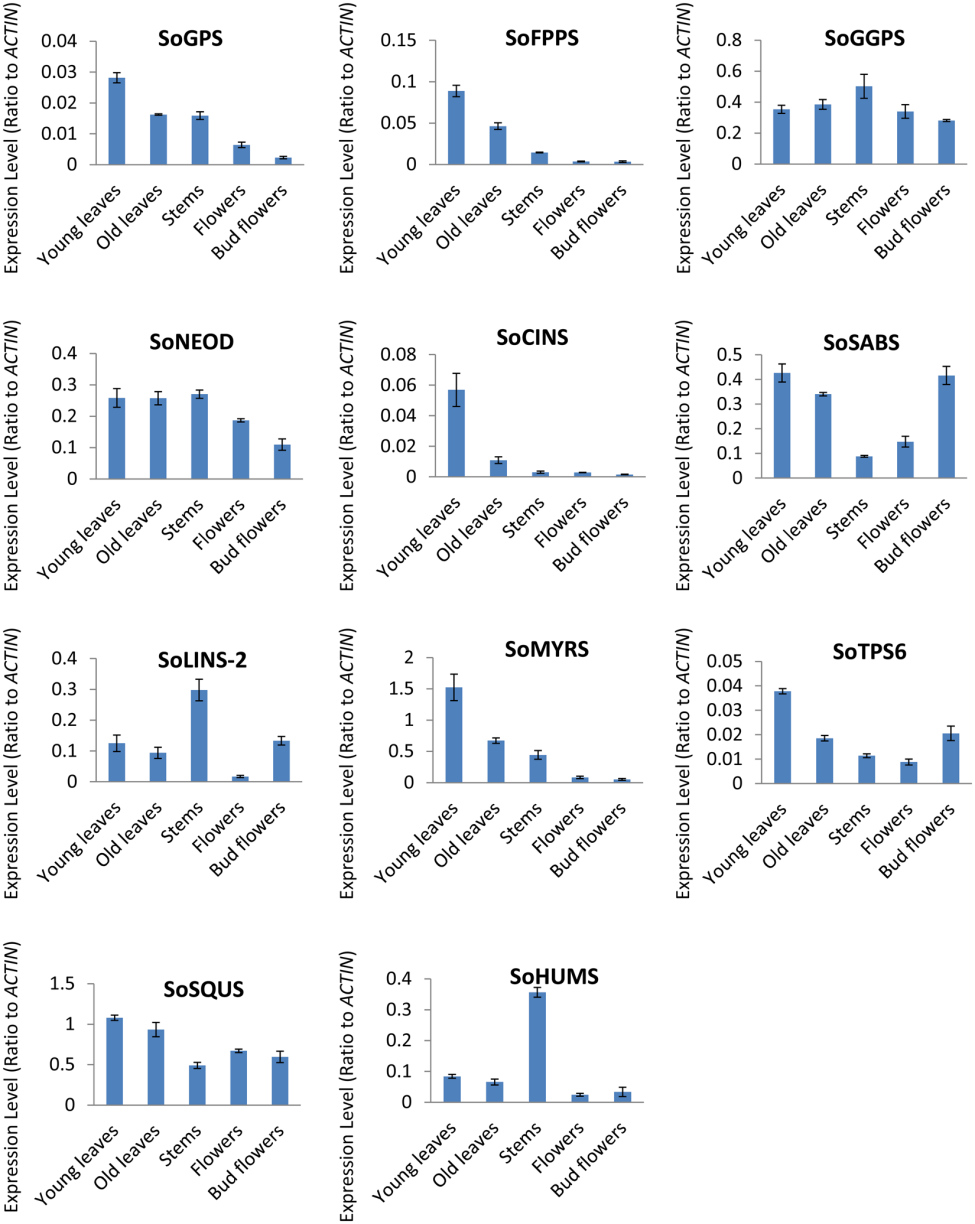
Quantitative RT-PCR validation of expression of terpene synthase genes selected from the DGE analysis in *S*. *officinalis*. Total RNAs were extracted from young leaves, old leaves, stem, flower and bud flower samples and the expression of *SoNEOD*, *SoGPS*, *SoFPPS*, *SoGGPS*, *SoMYRS*, *SoLINS*, *SoHUMS*, *SoTPS6*, *SoSQUS*, *SoSABS* and *SoCINS* genes were analysed using quantitative real-time. *SoACTIN* was used as the internal reference. The values are means ± SE of three biological replicates.

To determinate the reliability of the Illumina HiSeq 2000 read analysis, eleven candidate genes with a higher differential expression were selected, and their expression profiles were compared within young leaf, old leaf, stem, flower and bud flower samples. Quantitative real-time (qRT) PCR was used to determine the ‘transcriptional control’, which indicates the number of mRNA copies of the enzyme that complements the end-product quantity. Therefore, the correlation between the *TPS* mRNAs with their products and the end-products showed a relationship between the chosen differentially expressed genes (DEGs), monoterpene synthase (*SoGPS*; comp20551_c0), sesquiterpene synthase (*SoFPS*2; comp10352_c0), diterpene synthase (*SoGGPS*; comp25415_c0), myrcene/ocimene synthase (*SoMYRS*; comp11163_c0) 1,8-cineole synthase (*SoCINS*; comp26990_c0), (3S)-linalool synthase-2 (*SoLINS*; comp6814_c0), α-humulene/β-caryophyllene synthase (*SoHUMS*;comp101158_c0), (−)-germacrene D synthase (*SoTPS6*; comp26367_c0), squalene monooxygenase (*SoSQUS*; comp26984_c0), (+)-sabinene synthase (*SoSABS*; comp18462_c0) and (+)-neomenthol dehydrogenase (*SoNEOD*; comp10962_c0) and the terpenoid biosynthesis pathway of *S*. *officinalis*. *SoACTIN* was used as an internal reference gene (Supplementary Table S9). The expression patterns of the eleven selected DEGs in the young leaf, old leaf, stem, flower, and bud flower samples were examined (Fig. 5) by qRT-PCR, and the results were consistent with the results from the Illumina HiSeq 2000 read analysis. At the current stage, we may be able to answer the question which terpenoid compounds accumulate mostly in which *S*. *officinalis* tissue. From our results, we found that *SoGPS*, *SoFPS*, *SoMYRS*, and *SoCINS* genes showed the highest expression levels in young leaves, followed by old leaves, stems, flowers and bud flowers. Moreover, (+)-sabinene synthase (*So*SABS) genes showed the highest expression levels in young leaves, followed by bud flowers, old leaves, flowers, and stems. (3S)-linalool synthase (*SoLINS*) genes showed the highest expression levels in stems, followed by bud flowers, young leaves, old leaves, and flowers. Furthermore, diterpene synthase gene *SoGGPS* showed the highest expression levels in stems, followed by old leaves, young leaves, flowers and bud flowers. On the other hand, *SoTPS6* gene showed the highest expression levels in young leaves followed by bud flowers, old leaves, stems, and flowers. Squalene monooxygenase (*SoSQUS*) gene showed the highest expression levels in young leaves followed by old leaves, flowers, bud flowers, and stems. Finally, a α-humulene/β-caryophyllene synthase (*SoHUMS*) gene showed the highest expression levels in stems, followed by young leaves, old leaves, bud flowers and flowers. These results were compatible with our GC-MS analysis data, indicating that indicated that the main group of terpenes in young leaves, old leaves and stems consisted of mono- and sesquiterpenes. According to the findings of the GC-MS analysis, the major monoterpene compound in young and old leaves was 1,8-cineole (Table 1). Therefore, we suggest that young leaves are the primary site for monoterpene, sesquiterpene and 1,8-cineole synthase biosynthesis and accumulation, followed by old leaves, and then stems. These results are in agreement with those of previous studies^38,39^ that reported that the main monoterpenes in *S*. *officinalis* and other *Salvia* plant species are formed and accumulate in very young leaf epidermal glands, as the formation of most epidermal glands and the accumulation of the monoterpenes take a very short time in young leaf tissues. Consequently, in our study we focused on young leaves in which these genes are expressed at higher levels; monoterpenes and sesquiterpenes are also formed at their highest levels in young leaves. In addition, from our study, we found a correlation between the 1,8-cineole accumulation and 1,8-cineole synthase (*SoCINS*) expression levels in different tissues. For instance, the most abundant 1,8-cineole accumulation and highest *SoCINS* expression were in young leaves, followed by old leaves, stems, flowers and bud flowers (Table 1 and Fig. 1). Our results are in line with those of previous studies^40–47^ that reported that the monoterpene levels are thought to be mainly controlled transcriptionally producing different TPS enzymes. (+)-Neomenthol was not detected by GC-MS analysis as was expected from gene expression analysis, showing the expression of a putative neomenthol dehydrogynase gene that were detected in the Illumina HiSeq 2000 reads and qRT-PCR. This could be due to other unknown reasons^48^. The combination of the analysed data reads from the Illumina HiSeq 2000, qRT- PCR and the GC-MS will pave the way for understanding the complex mechanisms for controlling and regulating the diverse production of terpene compounds.

### Functional characterization of *TPS* genes in transgenic *N*. *tabacum* leaves

**Figure 6:**
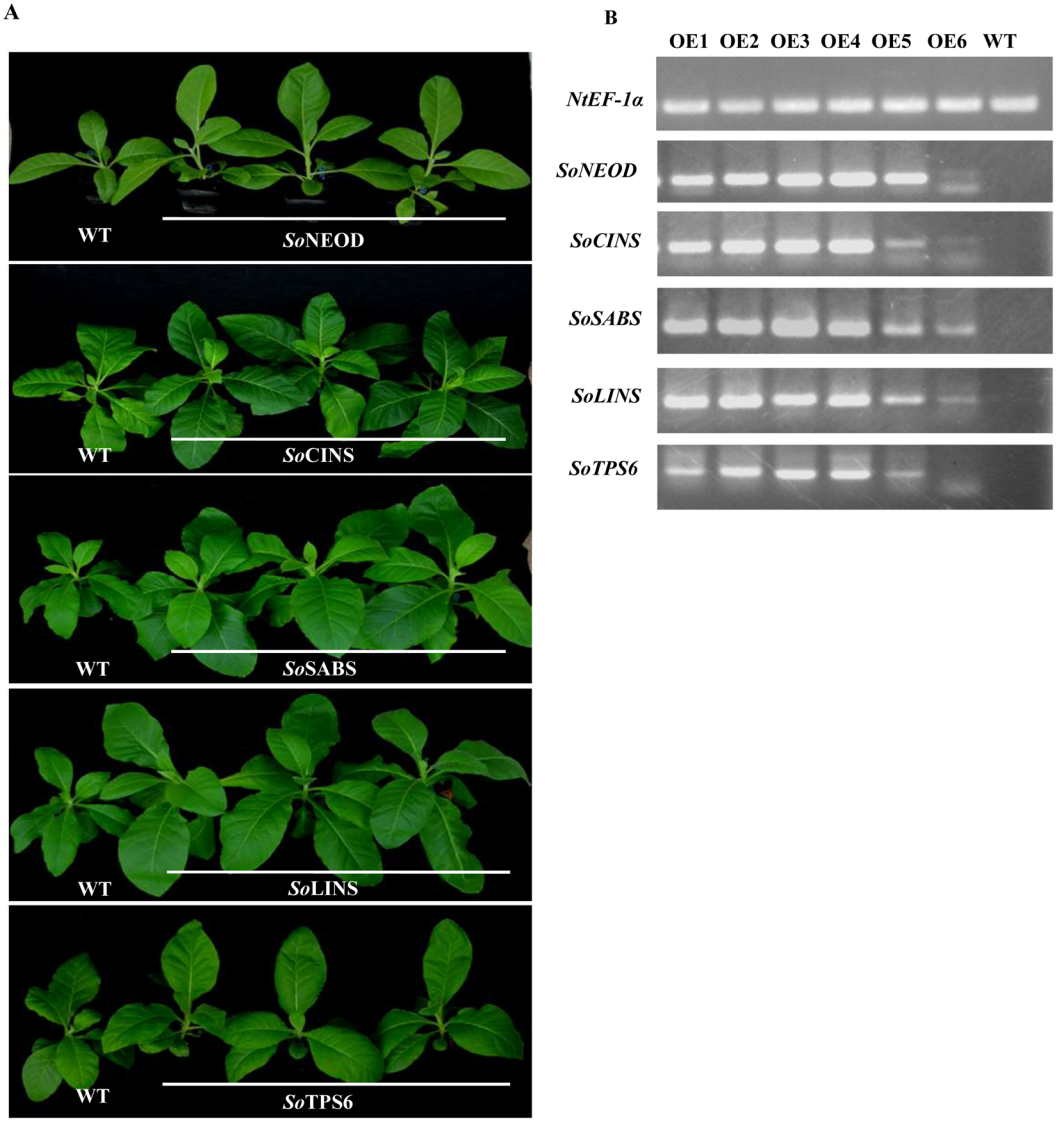
Overexpression of five *S*. *officinalis TPS* genes in transgenic *N*. *tabacum*. (**A**) Transgenic tobacco plants after adaptation to soil pots. (**B**) Semiquantitative RT-PCR analysis of the terpene synthase gene expression.

**Figure 7:**
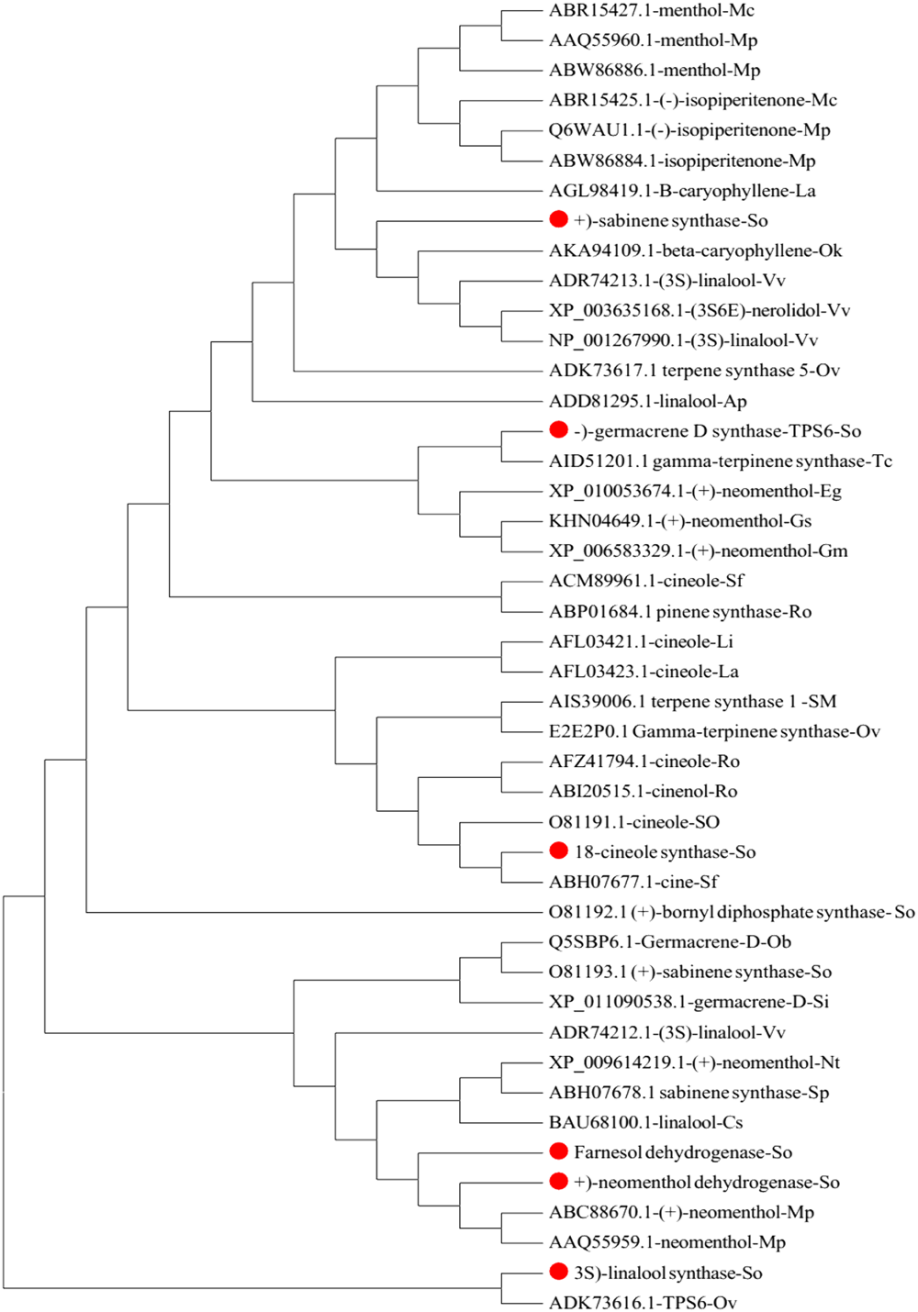
Phylogenetic analysis of terpenoid biosynthesis genes from *S*. *officinalis* and other plants. MEGA6 program was used for building up the tree through neighbor joining method.

To test *N*. *tabacum* in a transgenic expression system for the production of *Salvia* terpenes, the following genes were selected from *S*. *officinalis*: (+)-neomenthol dehydrogenase (*NEOD*), 1,8-cineole synthase (*CINS*), (+)-sabinene synthase (*SABS*), (3S)-linalool synthase (*LINS*), and (−)-germacrene D synthase (*TPS6*) encoded by *SoNEOD*, *SoCINS*, *SoSABS*, *SoLINS*, and *SoTPS6*, respectively.The stable constitutive expression of the Salvia *TPS* genes in tobacco was carried out by the infection of *N*. *tabacum* leaves using *A*. *tumefaciens* strain EHA105 carrying pB2GW7-*NEOD*, pB2GW7-*CINS*, pB2GW7-*SABS*, pB2GW7-*LINS*, and pB2GW7-*TPS6* under the control of 35S promoter. Samples of infected were collected 45 days after transgenic tobacco acclimatization (Fig. 6A). We then used semiquantitative RT-PCR to analyse the positive transgenic tobacco and assessed the expression levels of terpene genes from the different samples (Fig. 6B and Supplementary Fig. S4). The terpenes were extracted with hexane and analysed by GC-MS. The mono-, sesqui-, di- and triterpene peaks were clearly detected, and the type and amount of compounds represented by the percentage of peak area (% peak area). Compounds were identified by comparing the mass spectra of the compounds with mass spectra libraries. The annotation of the detected components was also confirmed by comparing them with the published references and extracts of tobacco cultivars, which produce different types and amounts of terpenes^49,50^. Overexpression of *SoNEOD*, *SoCINS*, *SoSABS*, *SoLINS*, and *SoTPS6* genes in tobacco plants produced different amounts of mono-, sesqui-, di-, and triterpenes and other terpenoids. Moreover, from the results shown in Table 4, Supplementary Fig. 5 and Table S10, we found that the transient expression of the different TPS genes from Salvia produced different types and amounts of mono-, sesqui-, di-, and triterpenes and other terpenoid compounds. We also could show a high similarity between the product patterns of *TPS* genes from Salvia with these from other plant species (Fig. 7).

**Table 4:** The major terpenoid compositions in transgenic *N. tabacum* leaves overexpressing *SoNEOD, SoCINS,SoSABS, SoLINS, and SoTPS6*.

N	Compound name	Retention time (min.)	Retention time index	Major fragment ions (*m/z*)	Formula	Molecular Mass (g mol^-1^)	Terpene Type	% Peak area					
								W.T	*SoNEOD*	*SoCINS*	*SoSABS*	*SoLINS*	*SoTPS6*
1	L-.alpha.-Terpineol	20.607	1144	139,136,121,107,93,81,79	C10H18O	154.2493	Mono	—	—	0.05	—	—	—
2	L-(-)-Nicotine	26.372	1360.5	162,133,119,92,84,51	C10H14N2	162.2316		22.49	0.08	—	0.71	—	0.35
3	trans-.beta.-Ionone	30.596	1488	177,159,149,135,133,121,119	C13H20O	192.2973		—	—	0.05	0.17	—	—
4	Topanol;Stavox	31.146	1514.	220,205,177,161,145,115,105	C15H24O	220.3505	Sesqui	—	0.04	—	—	0.04	—
5	cis-Carveol	35.519	1226	137,134,123,119,109,105,95	C10H16O	152.2334	Mono	—	—	—	—	0.1	—
6	.alpha.-Campholenal	35.809	1122	137,119,108,81,67	C10H16O	152.2334	Mono	—	0.08	—	0.23	—	—
7	Menthofuran;	36.17	1163	150,108,79,77,39	C10H14O	150.2176	Mono	—	—	0.05	—	—	—
8	α-Bulnesene	45.066	1506	204,189,161,147,135,107,93	C15H24	204.3511	Sesqui	—	—		0.22	—	—
9	cis-9-Hexadecenal	45.266	1759	220,149,138,135,124,121,111	C16H30O	238.4088		—	—	—	0.33	—	—
10	Triadimefon	45.59	1997	208,181,128,110,85,57,41,29	C14H16ClN3O2	293.749		—	—	0.32	—	—	—
11	Cyclooctasiloxane, hexadecamethyl-	45.773	1688	415,401,355,281,221,147,73	C16H48O8Si8	593.2315		—	0.29	—	—	1.57	1.04
12	7-Hexadecenal, (Z)-	45.855	2144	220,135,121,111,98,82,67,55	C16H30O	238.4088		—	—	1.7	—	—	—
13	β-Elemol	46.067	1547	204,189,161,135,121,107,93	C15H26O	222.3663	Sesqui	0.14	2.33	—	1	—	—
14	(+) Ledol	46.099	1599	204,189,161,122,109,69,43,41	C15H26O	222.3663	Sesqui	—	0.2	0.1	—	0.33	—
15	Ledol	46.367	1600	204,189,161,147,122,109,81,69	C15H26O	222.3663	Sesqui	—	—	0.37	1.22	—	—
16	d-Ledol	46.431	1602	204,189,161,147,133,122,119	C15H26O	222.3663	Sesqui	—	—	—	—	0.51	—
17	Retinol, acetate	46.635	2531	268,253,145,119,105,91,81,43	C22H32O2	328.4883		0.32	1.3	—	—	—	—
18	Phytol	46.736	2110	278,196,179,137,123,95,72	C20H40O	296.5310	Diter	—	—	—	0.74	0.36	5.11
19	cis-Phytol	47.331	2114	278,193,179,151,137,123,109	C20H40O	296.5310	Diter	3.19	6.28	6.75	10.84	3.92	—
20	Carveol	48.108	1225	137,134,119,105,93,92,91,77,41	C10H16O	152.2334	Mono	—	—	—	—	0.27	—
21	α-Limonene diepoxide	48.755	1294	137,123,95,79,67	C10H16O2	168.2328	Mono	—	—	—	2.34		—
22	Ledol	48.982	1580	204,189,161,135,133,109,107,69	C15H26O	222.3663	Sesqui	—	—	—	0.28	2.22	—
23	Caryophyllene	50.085	1420	204,189,161,133,120,93,79,69	C15H24	204.3511	Sesqui	30.71	31.53	—	45.45	54.04	72.67
24	Isopulegol	50.305	1143	154,136,121,93,81,67	C10H18O	154.2493	Mono	—	0.12		—	—	—
25	.alpha.-Guaiene	50.47	1437	204,189,161,147,133,105,93,79	C15H24	204.3511	Diter	—	—	0.57	—	—	—
26	5.beta.,7.beta.H,10.alpha.-Eudesm-11-en-1.alpha.-ol	50.703	1638	222,207,161,137,125,95,81,55	C15H26O	222.3663	Sesqui	—	—	—	—	0.24	—
27	Lycopene	51.348	3949	457,413,347,321,269,203,177	C40H56	536.89	Sesqui	—	—	0.19	0.39	—	—
28	α-Limonene diepoxide	53.194	1294	137,123,95,79,67	C10H16O2	168.2328	Mono	—	—	—	—	0.15	—
29	α-Elemol	53.591	1546	204,189,161,121,107,93,81,59	C15H26O	222.3663	Sesqui		0.53	—	—	—	—
30	Triadimenol	53.554	2023	168,128,112,99,70,59,43,41,29	C14H18ClN3O2	295.765	Sesqui	—	—	0.2	—	—	—
31	Squalene	53.568	2833	367,341,299,203,175,161,137	C30H50	410.7180	Triter	—	—	—	—	0.48	—
32	Verbenol	53.994	1130	137,119,94,81,59	C10H16O	152.2334	Sesqui	—	—	—	—	0.1	—
33	Caryophyllene oxide	54.241	1546	220,205,177,161,135,121,93,79	C15H24O	220.3505	Sesqui	—	—	0.09	—	—	—
34	Globulol	54.833	1581	204,149,161,109,82,69,55,43,41	C15H26O	222.3663	Sesqui	—	—	—	—	0.05	—
35	Caryophyllene oxide	58.543	1583	205,177,149,138,109,93,91,79	C15H24O	220.3505	Sesqui	—	—	0.13	—	—	—
36	Dotriacontane	74.491	3200	407,379,337,295,253,225,127	C32H66	450.8664		5.48	—	—	—	—	—
37	Trans-Squalene	74.571	2835	341,328,299,203,149,127,81,69	C30H50	410.7180	Triter	—	—	—	0.13	—	1.24

The putative functions of *TPS* genes isolated from *S*. *officinalis* were initially predicted according to the conserved motifs using the InterPro protein sequence analysis & classification (http://www.ebi.ac.uk/interpro/) database. The SoCINO protein with a 591-aa length has an N-terminal domain (IPR001906) from 66–279 aa and a metal-binding domain (IPR005630) from 265–589 aa; inside the latter domain are two motifs: one is an RR (x) 8 W motif (RRTGGYQPTLW) starting at 57 aa, and the other one is a DDxxD motif (DDVFD) starting at 345 aa. On the other hand, the *So*LINA protein is 505 aa in length. This protein has an N-terminal domain (IPR001906) from 1–183 aa and a metal-binding domain (IPR005630) from 171–497 aa, and inside the last domain are DDxxD conserved motifs (DDIFD) starting at 250 aa. Finally, the protein sequences contained one or two of this domain belonging to the *TPS* gene family.

Croteau and coworkers had revealed the carbocationic reaction mechanism for all monoterpene synthases by reporting that the reaction was initiated by the divalent metal ion-dependent ionization of the substrate. The resulting cationic intermediate undergoes a series of hydride shifts or other rearrangements and cyclizations until the reaction was terminated by the addition of a nucleophile or proton loss. They also illustrated this reaction mechanism by studying the native enzymes with substrate inhibitors, analogues and intermediates^51,52^. Moreover, Croteau *et al*. 1987^53^ elucidated the preliminary conversion of the geranyl cation to the tertiary linalyl cation to facilitate cyclization to a six-membered ring. Afterwards, the linalyl cation provides the cyclic α-terpinyl cation; this is an important branching point intermediate in the formation of all cyclic monoterpenes because multiple terpene products can be obtained through electrophilic attack of C1 on the C6–C7 linalyl cation double bond and from the α-terpinyl cation^53^. From the previous discussion, the reaction mechanisms of monoterpene synthases are highly reticulate. The individual intermediate may have multiple fates, which suggests the explanation for the ability of terpene enzymes to make various terpene products^54–57^. On the other hand, the carbocationic reaction mechanism that uses sesquiterpene synthase to form sesquiterpenes by catalysing farnesyl pyrophosphate (FPP) recycling is similar to the reaction mechanism by those monoterpene synthases. Moreover, the larger carbon skeleton of FPP and the presence of three double bonds instead of two suggest a rationale for increases of the structural diversity of the sesquiterpene products. Furthermore, the initial cyclization reactions for sesquiterpene synthases can be divided into two types. Type one involves cyclization of the initially formed farnesyl cation to yield 11-membered (E)-humulyl cation) rings of large size and a C2–C3 double bond (this type has no barrier to cyclization). The second type involves cyclization that proceeds after the tertiary nerolidyl cation produced from preliminary isomerization of the C2–C3 double bond. This isomerization mechanism is directly analogous to the isomerization of GPP to yield a linalyl cation in monoterpene synthesis. The nerolidyl cation is considered an intermediate in the sesquiterpene synthase mechanism^58–62^.

Collectively, we can state that the ability of *TPS* genes to convert a prenyl diphosphate substrate into diverse products during different reaction cycles is one of the unique traits of this type of enzyme. As described above, this property is found in the majority of all characterized monoterpene and sesquiterpene synthases. However, some monoterpene and sesquiterpene synthases can catalyse substrates into a single product, and the proteins may have specific methods for multiple product formations. For example, γ-humulene synthase from *A*. *grandis* has two DDxxD motifs located on opposite sides and can generate 52 different sesquiterpenes. This protein is able to bind substrates with two different conformations, resulting in different sets of products^63^. In another example regarding the first monoterpene synthase cloned from *Salvia officinalis*, (+)-sabinene synthase produces 63% (+)-sabinene but also 21% γ-terpinene, 7.0% terpinolene, 6.5% limonene and 2.5% myrcene in *in vitro* assays^64^. These additional monoterpene products or their immediate metabolites are also found in the monoterpene-rich essential oil of the *S*. *officinalis* plant.

## Conclusion

In this study, a large, high-quality transcriptome database was established for *S*. *officinalis* leaves using NGS technology to characterize and to identify genes that are related to terpenoid biosynthesis. Using *de novo* sequencing and analysis of the *S*. *officinalis* transcriptome data via the Illumina HiSeq 2000 system, we identified many genes that encode enzymes involved in the terpenoid biosynthesis pathway. The purpose of identifying these genes is not only to facilitate functional studies but also to develop biotechnology for improving the production of medicinal ingredients through metabolic engineering. We profiled terpenoids from three tissues of *S*. *officinalis* and used qRT-PCR to determine the correlation between the expression levels of *TPS* genes and the end-products. By combining the transcriptome and metabolome analyses with RNA-Seq or qRT-PCR with GC-MS approaches, this study paves the way for understanding the complex metabolic genes for the production of the diverse terpene compounds in garden sage. The results from our study will allow to understand the specific activities of TPSs in *S*. *officinalis* for the production of interesting compounds and to develop new technology for utilization.

To our knowledge, this is the first study to use Illumina HiSeq 2000 paired-end sequencing technology to investigate the global transcriptome of *S*. *officinalis*. The valuable genetic resource in *Salvia* will provide the foundation for future genetic and functional genomic research on *S*. *officinalis* or closely related species. We further studied the functions of various *S*. *officinalis TPS* genes, including *SoNEOD*, *SoCINS*, *SoSABS*, *SoLINS*, and *SoTPS6*, by stably expressing these genes in *N*. *tabacum* transgenic plants. *SoNEOD*, *SoCINS*, *SoSABS*, *SoLINS*, and *SoTPS6* were functionally expressed in the leaves of *N*. *tabacum*, and these transgenes altered the levels of terpenoids, as confirmed by GC-MS analysis of extracted transgenic *N*. *tabacum* leaves. The GC-MS analysis revealed that these *S*. *officinalis* terpene synthases isolated from *S*. *officinalis* can convert a prenyl diphosphate substrate into diverse products, which is one of the unique traits of this type of enzyme. Our study provides new insights into our understanding of plant terpenoid biosynthesis and the potential for biotechnology application.

## Materials and Methods

### Plant materials and tissue collection

Seeds of *Salvia officinalis* were collected from the Egyptian Desert Gene Bank, North Sinai Research Station, Department of Plant Genetic Resources, Desert Research Center, Egypt, and grown at Huazhong Agricultural University, Wuhan, China. Different tissues were sampled from one-year-old *S*. *officinalis* plants. For RNA-Seq, three biological replicates from leaves were sampled and handled. Each replicate consisted of two young and two old leaves from the same plant. For qRT-PCR, three biological replicates were collected from the following five parts (young leaves, old leaves, stems, flowers and bud flowers). All samples were immediately frozen in liquid nitrogen and then stored at −80 °C until RNA extraction. Furthermore, another three biological replicates from the individual three fresh parts were collected for isolation of the essential oil.

### Isolation of chemical compounds

The correct method to reduce technical variability throughout a sampling procedure is essential to stop cell metabolism and to avoid leaking of metabolites during the various preparation steps before the actual metabolite extraction. Therefore, three biological replicates from each of the three fresh parts were immediately frozen on dry ice. In the laboratory, the frozen three biological replicates from each of the three fresh part samples were homogenized in liquid nitrogen with a mortar and pestle, after which the plant material (ca. 10 g) was directly soaked in n-hexane as a solvent in Amber storage bottles, 60 ml screw-top vials with silicone/PTFE septum lids (http://www.sigmaaldrich.com) were used to reduce loss of volatiles to the headspace then incubated with shaking at 37 °C and 200 rpm for 72 h. Afterward, the solvent was transferred using a glass pipette to a 10-ml glass centrifuge tube with screw-top vials with silicone/PTFE septum lids and centrifuged at 5,000 rpm for 10 minutes at 4 °C to remove plant debris. The supernatant was pipetted into glass vials with a screw cap and oil was concentrated until remaining 1.5 ml of concentrated oils under a stream of nitrogen gas with a nitrogen evaporator (Organomation) and water bath at room temperature (Toption-China-WD-12). The concentrated oils transferred to a fresh crimp vial amber glass, 1.5 ml screw-top vials with silicone/PTFE septum lids were used to reduce a loss of volatiles to the headspace. For absolute oil recovery, the remaining film crude oil in the internal surface of concentrated glass vials was dissolved in the minimum volume of n-hexane, thoroughly mixed and transferred to the same fresh crimp vial amber glass, 1.5 ml. And the crimp vial was placed on the autosampler of GC-MS system for GC-MS analysis, or each tube was covered with parafilm after closed with screw-top vials with silicone/PTFE septum lids and stored at −20 °C until GC-MS analysis.

### GC-MS analysis of essential oil components

GC analysis was performed using a Shimadzu model GCMS-QP2010 Ultra (Tokyo, Japan) system. An approximately 1 µl aliquot of each sample was injected (split ratios of 15:1) into a GC-MS equipped with an HP-5 fused silica capillary column (30 m × 0.25 mm ID, 0.25 µm film thickness). Helium was used as the carrier gas at a constant flow of 1.0 ml min^−1^. The mass spectra were monitored between 50–450 m/z. Temperature was initially under isothermal conditions at 60 °C for 10 min. Temperature was then increased at a rate of 4 °C min^−1^ to 220 °C, held isothermal at 220 °C for 10 min, increased by 1 °C min^−1^ to 240 °C, held isothermal at 240 °C for 2 min, and finally held isothermal for 10 min at 350 °C. The identification of the volatile constituents were done by parallel comparison of their recorded mass spectra with the data stored in the Wiley GC/MS Library (10^th^ Edition) (Wiley, New York, NY, USA), and the retention time index (http://massfinder.com/wiki/MassFinder_Analysing_your_own_data), with the Volatile Organic Compounds (VOC) Analysis S/W software, and the NIST Library (2014 edition), The Adams Library (http://essentialoilcomponentsbygcms.com/list-of-compounds-in-the-essential-oil-components-database/), and the Terpenoids Library (http://massfinder.com/wiki/Terpenoids_Library_List). The relative% amount of each component was calculated by comparing its average peak area to the total areas, as well as Retention time index. (All of the experiments were performed simultaneously three times under the same conditions for each isolation technique with total GC running time was 80 minutes.

### RNA extraction

Total RNAs from the three biological leaf replicates were extracted for RNA-Seq. Moreover, total RNAs from three biological replicates from each of the plant parts (young leaves, old leaves, stems, flowers and bud flowers) were extracted for qRT-PCR. Additionally, total RNAs from three biological replicates of transgenic *N*. *tabacum* were extracted for semiquantitative RT-PCR using the TRIzol Reagent (Invitrogen, USA) and treated with DNase I (Takara). RNA quality was examined on 1% agarose gels, and the purity was analysed using a Nano-Photometer® spectrophotometer (IMPLEN, CA, USA). RNA concentration was determined using a Qubit® RNA Assay Kit in a Qubit® 2.0 Fluorometer (Life Technologies, CA, USA). RNA pools were prepared for cDNA libraries by mixing equal volumes from the three RNAs replications in one tube.

### cDNA library preparation and sequencing

Three micrograms of RNA per sample were used for generating a sequencing library. cDNA was synthesized using an RNA Library Prep Kit for Illumina® (NEB, USA) for generated sequencing libraries according to the manufacturer’s instructions. The first strand of cDNA was synthesized in the presence of random hexamer primers and M-MuLV Reverse Transcriptase (RNase H), and the second strand of cDNA was synthesized in the presence of DNA polymerase I and RNase H. The remaining cDNA was converted into blunt ends in the presence of exonuclease/polymerase activities. After the adenylation of 3′ ends of DNA fragments, NEB Next, an adaptor with a hairpin loop structure, was ligated to prepare for hybridization. To select cDNA fragments of preferentially 150~200 bp in length, the library fragments were purified using an AMPure XP system (Beckman Coulter, Beverly, USA). Then, 3 μl of USER Enzyme (NEB, USA) was used with size-selected, adaptor-ligated cDNA at 37 °C for 15 min followed by 95 °C for 5 min. Afterwards, PCR was performed with Phusion High-Fidelity DNA polymerase, universal PCR primers and Index (X) Primer. Finally, PCR products were purified (AMPure XP system), and the library quality was assessed using an Agilent Bioanalyzer 2100 system (Agilent Technologies, CA, USA). Clustering of the index-coded samples was performed on a cBot Cluster Generation System using a TruSeq PE Cluster Kit v3-cBot-HS (Illumina) according to the manufacturer’s instructions (Novogene Experimental Department). After cluster generation, the library preparations were sequenced on an Illumina HiSeq 2000 platform, and paired-end reads were generated.

### Quality control

Raw data (raw reads) in fastq format were first processed through in-house Perl scripts. During this step, clean data (clean reads) were obtained by removing reads containing adapters, reads containing ploy-N and low-quality reads from the raw data. At the same time, Q20, Q30, GC content and sequence duplication level of the clean data were calculated. All of the downstream analyses were based on high-quality clean data.

### *De novo* transcriptome assembly

*De novo* assembly of the processed reads was carried out using Trinity program (Version: trinityaseq_r 2012-10-05)^20^, with the min_kmer_cov set to 2 by default and all other parameters set to default. The Trinity method consists of three software modules, (1) Inchworm, (2) Chrysalis and (3) Butterfly, applied sequentially to process large volumes of RNA-Seq reads. In the first step, read datasets were assembled into linear contigs by the first module (Inchworm program). The minimally overlapping contigs were then clustered into sets of connected components (build graph components) by the second module (Chrysalis program), and the transcripts were then constructed from each de Bruijn graph by the third software module (Butterfly program). Finally, the transcripts were clustered by similarity of correct match length beyond 80% for longer transcripts or 90% for shorter transcripts using the multiple sequence alignment tool.

### Annotation of unigenes

Unigenes were used as query sequences to search the annotation databases, including the NCBI non-redundant protein sequences database (NR) (http://www.ncbi.nlm.nih.gov/) and Swiss-Prot (a manually annotated and reviewed protein sequence database) (http://www.ebi.ac.uk/uniprot/), based on sequence homology to entries in the Gene Ontology (GO) database (http://www.geneontology.org/). Unigene sequences from *S*. *officinalis* were categorized into three general sections: biological process (BP), cellular component (CC) and molecular function (MF). Additionally, the unigenes were used as query sequences for searching the Kyoto Encyclopedia of Genes and Genome (KEGG) pathways database (http://www.genome.jp/kegg/) and the Pfam (Protein family) database (http://pfam.sanger.ac.uk/).

### Differential expression analysis

Expression levels of unigenes were normalized and calculated as the values of fragments per kilobase of transcripts per million mapped fragments (FPKM) during the assembly and clustering process. Differential expression analysis of unigenes was performed using the DESeq R package (1.10.1). DESeq provides statistical routines for assessing the differential gene expression in leaf tissues and assigns genes as differentially expressed when the P-value < 0.05. P-value results were corrected using the Benjamini and Hochberg approach for controlling the false discovery rate (FDR)^65^.

### Quantitative real-time PCR (qRT-PCR) analysis

Quantitative RT-PCR was performed using an IQ^TM^ 5 Multicolor Real-Time PCR Detection System (Bio-Rad, USA) as described previously^66^ with SYBR Green Master (ROX) (Newbio Industry, China) following the manufacturer’s instructions at a total reaction volume of 20 µl. Gene-specific primers for *So*Actin as a reference gene and for the other eleven genes (*SoNEOD*, *SoGPS*, *SoFPPS*, *SoGGPS*, *SoMYRS*, *SoLINS*, *SoHUMS*, *SoTPS6*, *SoSQUS*, *SoSABS* and *SoCINS*) involved in the biosynthesis of terpenes were designed using the primer designing tools of IDTdna (http://www.idtdna.com), as listed in Supplementary Table S9. The quantitative RT-PCR conditions were set as standard conditions: 95 °C for 3 min, 40 cycles of amplification (95 °C for 10 s, 60 or 58 °C for 30 s and 72 °C for 20 s), and a final extension at 65 °C for 1 min. The gene expression was normalized using *SoActin* as a reference gene. The relative expression levels were calculated by comparing the cycle thresholds (CTs) of the target genes with that of the reference gene *SoActin* using the 2^−ΔΔCt^ method^67,68^. The sizes of amplification products were 140–160 bp. The quantified data were analysed using the Bio-Rad IQ^TM^ 5 Multicolor Real-Time Manager software. Finally, the relative expression levels of *SoNEOD*, *SoGPS*, *SoFPPS*, *SoGGPS*, *SoMYRS*, *SoLINS*, *SoHUMS*, *SoTPS6*, *SoSQUS*, *SoSABS*, and *SoCINS* were detected. All reactions were performed with three replications.

### Identification of simple sequence repeats (SSRs)

All of the transcripts of *S*. *officinalis* were analysed with the MISA program version 1.0 (http://pgrc.ipkgatersleben.de/misa/misa.html) for the detection of SSR motifs that have mono- to hexanucleotide repeats. In addition, primers for each SSR were designed using Primer3 version 2.3.5 (http://primer3.sourceforge.-net/releases.php). The minimum number of SSR repeat units during analysis was ≥24 for mono- and dinucleotides and was 8, 7, 7, and 9 for tri-, tetra-, penta-, and hexanucleotide repeats, respectively. The default parameters corresponding to each unit size of the minimum number of repetitions were 1–10, 2–6, 3, 5, 4, 5, 5, 5, and 6-5 for Unigene SSR detection.

### Full-length terpene synthase cDNA clones and vectors

Full-length cDNAs for *SoNEOD*, *SoCINS*, *SoSABS*, *SoLINS* and *SoTPS6* were obtained by PCR amplification using short and long gene-specific primers (Supplementary Table S11) based on RNA-Seq sequence information from the transcriptome sequencing of *S*. *officinalis* leaves. Leaf cDNA was used as a template for the initial PCR amplification and performed using short primers with the KOD-Plus DNA polymerase (Novagen) under the following PCR conditions: 3 min at 94 °C followed by 10 s at 98 °C; 30 s at 60, 57, 59, 60 or 60 °C (different annealing temperatures), 1.5 min at 68 °C, and then 10 min at 68 °C. This process was repeated for 35 cycles. The cDNA was used as a template for PCR cloning using long primers with the KOD-Plus DNA polymerase for the Gateway pDONR221 vector. The amplified PCR products were purified and cloned into the Gateway entry vector pDONR221 using bp Clonase (Invitrogen, USA). The resulting pDONR221 constructs harbouring target genes were sequenced, and Gateway LR Clonase (Invitrogen, USA) was used for recombination into the destination vector pB2GW7 for tobacco transformation. All final constructs containing *SoNEOD*, *SoCINS*, *SoSABS*, *SoLINS* and *SoTPS6* were confirmed by sequencing.

### *Nicotiana* plant growth conditions and preparation of *Agrobacterium* cultures for infection

Wild-type *Nicotiana tabacum* plant seeds were grown under standard greenhouse conditions for ten days at the Wuhan Doublehelix Biology Science and Technology Company, Wuhan, Hubei, China. In addition, the constructs of pB2GW7 vectors with all inserted genes were introduced into *Agrobacterium tumefaciens* strain EHA105 by direct electroporation. Recombinant *A*. *tumefaciens* was grown for two days at 28 °C in solid LB media supplemented with 50 μg/ml each of rifampicin and spectinomycin. An individual colony of each sample was inoculated into 1.0 ml of liquid medium and grown at 28 °C under 200 rpm agitation overnight with the same media composition. After 24 h, 1.0 ml of each sample of liquid medium was transferred to a 250-ml conical flask containing 50 ml of LB media supplemented with the same compositions; the samples were grown at 28 °C in a shaker overnight until an optical density of 0.6–1.0 (OD 600) was reached. Overnight cell cultures were harvested by centrifugation at 5,000 rpm for 10 min at 4 °C, and the pellet was resuspended in the infection medium (50 ml of LB-free media + 50 μl of acetosyringone). *Nicotiana tabacum* plantlet leaves were collected from the greenhouse and sterilized by soaking in 70% ethanol for 30 s, soaking in 0.1% HgCl for 6 min, and then washing three times using autoclaved water each time for 3 min. Then, we cut the leaves into small pieces (1 cm × 1 cm) and discarded the petiole and midrib, after which the leaf pieces were soaked in Petri dishes with infection media for 10 min and stirred every 2 min. The transformation procedure was performed as described previously^69^. More than 15 individual transgenic tobacco lines were generated for each transgene and examined with PCR for positive transgenic lines of more than 10 lines for each transgene. The positive plants with good roots were transferred to the greenhouse for adaptation. Then, the transgenic tobacco plants were analysed for terpenoid profiling and target gene expression.

### Semiquantitative RT-PCR analysis

Semiquantitative real-time PCR was performed on an Eppendorf PCR (Eppendorf Mastercycler-Nexus GSX1, POCD Scientific, Australia) system with a total reaction volume of 25 µl. A gene-specific primer for NtEF-1α (*Nicotiana tabacum* EF-1-alpha-related GTP-binding protein) was used as a reference gene, and the other five gene-specific primers for *SoNEOD*, *SoCINS*, *SoSABS*, *SoLINS*, and *SoTPS6*, which are involved in the biosynthesis of terpenes, were designed using the primer designing tools of IDTdna (http://www.idtdna.com/scitools/Applications/RealTimePCR/); the primer sequences are listed in (Supplementary Table S9). The Semiquantitative RT-PCR conditions were as follows: predenaturation step at 95 °C for 4 min, 35 cycles of amplification (95 °C for 30 s, 58 or 60 °C for 30 s and 72 °C for 1 min), and a final extension step at 72 °C for 10 min. The PCR products were resolved on 1% agarose gel, and the expression levels of *NtEF-1α*, *SoNEOD*, *SoCINS*, *SoSABS*, *SoLINS*, and *SoTPS6* were detected.

#### Metabolite extraction from transgenic tobacco leaves

Terpenoid compounds from non-transgenic tobacco leaves (control) and transgenic tobacco leaves containing either *SoNEOD*, *SoCINS*, *SoSABS SoLINS*, or *SoTPS6* expression constructs were extracted and isolated. For this, three leaves from each transgenic tobacco line (one leaf from each plant) were homogenized in liquid nitrogen with a mortar and pestle, after which the plant material powder was directly soaked in n-hexane as a solvent in Amber storage bottles, 60 ml screw-top vials with silicone/PTFE septum lids (http://www.sigmaaldrich.com) were used to reduce loss of volatiles to the headspace then incubated with shaking at 37 °C and 200 rpm for 72 h. Afterward, the solvent was transferred using a glass pipette to a 10-ml glass centrifuge tube with screw-top vials with silicone/PTFE septum lids and centrifuged at 5,000 rpm for 10 minutes at 4 °C to remove plant debris. The supernatant was pipetted into glass vials with a screw cap and oil was concentrated until remaining 1.5 ml of concentrated oils under a stream of nitrogen gas with a nitrogen evaporator (Organomation) and water bath at room temperature (Toption-China-WD-12). The concentrated oils transferred to a fresh crimp vial amber glass, 1.5 ml screw-top vials with silicone/PTFE septum lids were used to reduce a loss of volatiles to the headspace. For absolute oil recovery, the remaining film crude oil in the internal surface of concentrated glass vials was dissolved in the minimum volume of n-hexane, thoroughly mixed and transferred to the same fresh crimp vial amber glass, 1.5 ml. And the crimp vial was placed on the autosampler of the gas chromatography mass spectrometer (GC-MS) system for GC-MS analysis, or each tube was covered with parafilm after closed with screw-top vials with silicone/PTFE septum lids and stored at −20 °C until GC-MS analysis. The same programme and standard conditions that were used for GC-MS analysis with *S*. *officinalis* essential oil components were applied.

### Gene accession number

Gene accession numbers: Genes studied here are accessible to GenBank. Salvia officinalis geranyl-diphosphate synthase (*SoGPS*, KY399788); farnesyl pyrophosphate synthetase (*SoFPPS*, KY399787); (3S)-linalool synthase (*SoLINS*, KY399786); terpene synthase 6 (*SoTPS6*, KY399785); (−)-germacrene D synthase (*SoSABS*, KY399783); *Salvia officinalis* 1,8-cineole synthase (*SoCINS*, KY399782); *Salvia officinalis* geranyl diphosphate synthase 2 (*SoGGPP*, KY486794); *Salvia officinalis* squalene monooxygenase (*SoSQUS*, KY486795).

## Electronic supplementary material

Supplementary Tables and Figures

## References

[1] Alziar, G. Catalogue synonymique des Salvia L. dumonde (Lamiaceae). 5 (3–4):87–136; 6(1–2, 4):79–115, 163–204; 7(1–2):59–109; 9(2–3):413–497; 10(3–4). (*I*.–*VI*. Biocosme Mesogeén) 33–117 (1988–1993).

[2] Atsuko T, Hiroshi O (2011). Phylogenetic relationships among subgenera, species, and varieties of Japanese Salvia L. (Lamiaceae). J Plant Res..

[3] Carretero-Paulet L (2002). Campositionm Expression and molecular analysis of the Arabidopsis DXR gene encoding 1-Deoxy-D-xylulose-5-phosphate reductoisomerase, the first committed enzyme of the 2-C-Methyl-D-erythritol-4-phosphate pathway. Plant Physiol..

[4] Zhao J, Davis LC, Verpoorte R (2005). Elicitor signal transduction leading to production of plant secondary metabolites. Biotechnology advances.

[5] Ward JA, Ponnala L, Weber CA (2012). Strategies for transcriptome analysis in nonmodel plants. Am J Bot..

[6] Shubhra R (2014). De novo sequencing and comparative analysis of holy and sweet basil transcriptomes. BMC Genomics..

[7] Hua WP, Zhang Y, Song J, Zhao LJ, Wang ZZ (2011). De novo transcriptome sequencing in Salvia miltiorrhiza to identify genes involved in the biosynthesis of active ingredients. Genomics..

[8] Hyun TK (2012). De novo transcriptome sequencing of Momordica cochinchinensisto identify genes involved in the carotenoid biosynthesis. Plant Mol Biol..

[9] Huang HH (2012). De novo characterization of the Chinese fir (Cunninghamia lanceolata) transcriptome and analysis of candidate genes involved in cellulose and lignin biosynthesis. BMC Genomics..

[10] Shi CY (2011). Deep sequencing of the Camellia sinens is transcriptome revealed candidate genes for major metabolic pathways of tea-specific compounds. BMC Genomics..

[11] Aziz RA, Hamed FK, Abdulah NA (2008). Determination of the main components of the essential oil extracted from *Salvia fruticosa* by sing GC and GC-MS DAMASCUS. J AGR SCI..

[12] Nadaf M, Nasrabadi M, Halimi M (2012). GC-MS Analysis of n-Hexane Extract from Aerial Parts of *Salvia nemorosa*. Middle-East Journal of Scientific Research..

[13] Christophe S (2012). Characterization of two genes for the biosynthesis of the labdane diterpene Z-abienol in tobacco (*Nicotiana tabacum*) glandular trichomes. The Plant Journal..

[14] Monica RL (2010). Comparative Chemical Composition and Antiproliferative Activity of Aerial Parts of *Salvia leriifolia* Benth. And *Salvia acetabulosa* L. Essential Oils Against Human Tumor Cell *In Vitro* Models. J Med Food..

[15] Fateme A-M, Mohammad HF, Abdolhossein R, Ali Z, Maryam S (2013). Volatile Constituents of *Salvia compressa* and Logochilus macranthus, two Labiatae Herbs Growing wild in Iran. Res. J.Recent Sci..

[16] Aziz RA, Hamed F, Abdulah NA (2008). Determination of the Main Components of the Essential Oil Extracted From *Salvia fruticosa* by sing GC and GC-MS DAMASCUS. J. AGR. SCI..

[17] Daniel JS (2004). Localization of Salvinorin A and Related Compounds in Glandular Trichomes of the Psychoactive Sage. Salvia divinorum. Annals of Botany..

[18] Rafidah A (2014). Volatile Profiling of Aromatic Traditional Medicinal Plant, *Polygonum minus* in Different Tissues and Its Biological Activities. Molecules..

[19] Wang Z (2010). *De novo* assembly and characterization of root transcriptome using Illumina paired-end sequencing and development of cSSR markers in sweetpotato (*Ipomoea batatas*). BMC Genomics..

[20] Kim HA (2014). High-Throughput Sequencing and De Novo assembly of *Brassica oleracea* var. Capitata L. for transcriptome Analysis. PLoS One..

[21] Liang C, Liu X, Yiu S-M, Lim BL (2013). *De novo* assembly and characterization of *Camelina sativa* transcriptome by paired-end sequencing. BMC Genomics..

[22] Grabherr MG, Haas BJ, Yassour M, Levin JZ, Thompson DA (2011). Full-length transcriptome assembly from RNA-Seq data without a reference genome. Nat Biotechnol..

[23] Annadurai RS (2013). *De Novo* transcriptome assembly (NGS) of Curcuma longa L. rhizome reveals novel transcripts related to anticancer and antimalarial terpenoids. PLoS One..

[24] An J (2014). Transcriptome profiling to discover putative genes associated with paraquat resistance in goosegrass (*Eleusine indica L*.). PLoS One..

[25] Yan W (2013). *De novo* transcriptome sequencing of radish (*Raphanus sativus* L.) and analysis of major genes involved in glucosinolate metabolism. BMC Genomics..

[26] Huang LL, Yang X, Sun P, Tong W, Hu SQ (2012). The first Illumina-based de novo transcriptome sequencing and analysis of safflower flowers. PLoS One..

[27] Gahlan P (2012). *De novo* sequencing and characterization of *Picrorhiza kurrooa* transcriptome at two temperatures showed major transcriptome adjustments. BMC Genomics..

[28] Yang L (2013). Transcriptome analysis of medicinal plant *Salvia miltiorrhiza* and identification of genes related to tanshinone biosynthesis. PLoS One..

[29] Xie F (2012). De novo sequencing and a comprehensive analysis of purple sweet potato (*Impomoea batatas* L.) transcriptome. Planta..

[30] Kanehisa M, Goto S (2000). KEGG: Kyoto encyclopedia of genes and genomes. Nucleic Acids Res..

[31] Virginie D (2001). Crystal structure of isopentenyl diphosphate:dimethylallyl diphosphate isomerase. The EMBO Journal..

[32] Dorothea T (2006). Terpene synthases and the regulation, diversity and biological roles of terpene metabolism. Current Opinion in Plant Biology..

[33] Douglas JMG, Rodney C (1995). Terpenoid Metabolism. The Plant Cell..

[34] Nagegowda DA (2010). Plant volatile terpenoid metabolism: Biosynthetic genes, transcriptional regulation and subcellular compartmentation. FEBS Lett..

[35] Misra, R. C., Maiti, P., Chanotiya, C. S., Shanker, K. & Ghosh, S. Methyl jasmonate-elicited transcriptional responses and pentacyclic triterpenoid biosynthesis in sweet basil. *Plant Physiol*. https://doi.org/10.1104/pp. 113.232884 (2014). 10.1104/pp.113.232884 PMC3912077 24367017

[36] Huang L (2012). Molecular characterization of the pentacyclic triterpenoid biosynthetic pathway in *Catharanthus roseus*. Planta..

[37] Verma P, Shah N, Bhatia S (2013). Development of an expressed gene catalogue and molecular markers from the de novo assembly of short sequence reads of the lentil (*Lens culinarisMedik*.) transcriptome. Plant Biotechnol J..

[38] Sabine G-G, Corinna S, Ralf S, Johannes N (2012). Seasonal influence on gene expression of monoterpene synthases in *Salvia officinalis* (Lamiaceae). J Plant Physiolol..

[39] Croteau R, Felton M, Karp F, Kjonaas R (1981). Relationship of camphor biosynthesis to leaf development in sage *Salvia officinalis*. Plant Physiol..

[40] Dudareva N, Cseke L, Blanc VM, Pichersky E (1996). Evolution of floral scent in Clarkia: novel patterns of S-linalool synthase gene expression in the *C*. *breweri* flower. Plant Cell..

[41] McConkey ME, Gershenzon J, Croteau RB (2000). Developmental regulation of monoterpene biosynthesis in the glandular trichomes of peppermint. Plant Physiol..

[42] Mahmoud SS, Croteau RB (2003). Menthofuran regulates essential oil biosynthesis in peppermint by controlling a downstream monoterpene reductase. Proc Natl Acad Sci..

[43] Mahmoud SS, Williams M, Corteau RB (2004). Cosuppression of limonene-3-hydroxylase in peppermint promotes accumulation of limonene in the essential oil. Phytochemistry..

[44] Xie Z, Kapteyn J, Gang DR (2008). A systems biology investigation of the MEP/terpenoid and shikimate/phenylpropanoid pathways points to multiple levels of metabolic control in sweet basil glandular trichomes. Plant J..

[45] Lane A, Boecklemann A, Woronuk GN, Sarker L, Mahmoud SS (2010). A genomics resource for investigating regulation of essential oil production in *Lavandula angustifolia*. Planta..

[46] Schmiderer C, Grausgruber-Gröger S, Grassi P, Steinborn R, Novak J (2010). Influence of gibberellin and daminozide on the expression of terpene synthases in common sage (*Salvia officinalis*). J Plant Physiol..

[47] Kampranis SC (2007). Rational conversion of substrate and product specificity in a Salvia monoterpene synthase: structural insights into the evolution of terpene synthase function. Plant Cell..

[48] Xianzhong Z, Hongjian G, Lifen Z, Donghong L, Xingqian Y (2012). Extraction of essential oil from discarded tobacco leaves by solvent extraction and steam distillation, and identification of its chemical composition. Ind.Crops Prod..

[49] Fumin P, Liangquan S, Baizhan L, Hongwu T, Shaomin L (2004). Comparison of different extraction methods: steam distillation, simultaneous distillation and extraction and headspace co-distillation, used for the analysis of the volatile components in aged flue-cured tobacco leaves. J. Chromatogr. A..

[50] Natalia D (2003). (*E*)-β-Ocimene and Myrcene Synthase Genes of Floral Scent Biosynthesis in Snapdragon. The Plant Cell..

[51] Diane M M (2010). Functional annotation, genome organization and phylogeny of the grapevine (*Vitis vinifera*) terpene synthase gene family based on genome assembly, FLcDNA cloning, and enzyme assays. BMC Plant Biol..

[52] Rodney C, Mark F, Robert CR (1980). Biosynthesis of monoterpenes –conversion of the acyclic precursor’s geranyl pyrophosphate and nerylpyrophosphate to the rearranged monoterpenes fenchol and fenchone by a soluble enzyme preparation from fennel (*Foeniculum vulgare*). Arch. Biochem. Biophys..

[53] Rodney C (1987). Biosynthesis and catabolism of monoterpenoids. Chem. Rev..

[54] Wise ML, Rodney C (1999). Comprehensive Natural Products Chemistry, Isoprenoids Including Caroteinoids and Steroids. Elsevier, Amsterdam..

[55] Lücker J (2002). Monoterpene biosynthesis in lemon (Citrus Limon) – cDNA isolation and functional analysis of four monoterpene synthases. Eur. J. Biochem..

[56] Takehiko S (2004). Molecular cloning and functional characterization of four monoterpene synthase genes from Citrus unshiu Marc. Plant Sci..

[57] Huber DP (2005). Characterization of four terpene synthase cDNAs from methyl jasmonateinduced Douglas-fir, Pseudotsuga menziesii. Phytochemistry..

[58] Martin DM, Bohlmann J (2004). Identification of Vitis vinifera (−)-alpha-terpineol synthase by in silico screening of full-length cDNA ESTs and functional characterization of recombinant terpene synthase. Phytochemistry..

[59] David EC, Stephen S, Pushpalatha PNM (1981). Trichodiene biosynthesis and the enzymatic cyclization of farnesyl pyrophosphate. J. Am. Chem. Soc..

[60] David EC, Guohan Y (1994). Trichodiene synthase – stereochemical studies of the cryptic allylic diphosphate isomerase activity using an anomalous substrate. J. Org. Chem..

[61] David EC, Manish T (1995). Epicubenol synthase and the stereochemistry of the enzymatic cyclization of farnesyl and nerolidyl diphosphate. J. Am. Chem. Soc..

[62] Iris A (1998). The enzymatic cyclization of nerolidyl diphosphate by delta cadinene synthase from cotton stele tissue infected with Verticillium dahlia. Phytochemistry..

[63] Steele CL, Crock J, Bohlmann J, Croteau R (1998). Sesquiterpene synthases from grand fir (*Abies grandis*) – Comparison of constitutive and wound-induced activities, and cDNA isolation, characterization and bacterial expression of delta-selinene synthase and gamma-humulene synthase. J. Biol. Chem..

[64] Wise ML, Savage TJ, Katahira E, Croteau R (1998). Monoterpene synthases from common sage (*Salvia officinalis*) – cDNA isolation, characterization, and functional expression of (+)-sabinene synthase, 1, 8-cineole synthase, and (+)- bornyl diphosphate synthase. J. Biol. Chem..

[65] Anders, S., Huber, W. Differential expression analysis for sequence count data. *Genome Biology*, 10.1186/gb-2010-11-10-r106 (2010). 10.1186/gb-2010-11-10-r106 PMC3218662 20979621

[66] Li P (2016). Metabolic engineering of proantho-cyanidin production by repressing the isoflavone pathways and redirecting anthocyanidin precursor flux in legume. Plant Biotechnol J..

[67] Livak KJ, Schmittgen TD (2001). Analysis of relative gene expression data using real-time quantitative PCR and the 2^−ΔΔCT^ method. Methods..

[68] Hongmei L (2014). Transcriptional data mining of *Salvia miltiorrhiza* in response to methyl jasmonate to examine the mechanism of bioactive compound biosynthesis and regulation. Physiologia Plantarum..

[69] Sunjung, P. *Agrobacterium tumefaciens* –mediated transformation of tobacco (*Nicotiana tabacum* L.) leaf disks: evaluation of the co-cultivation conditions to increase β -Glucuronidase gene activity. (Master’s dissertation). Retrieved from http://etd.lsu.edu/docs/available/etd-07052006-173930/unrestricted/Park_thesis.Pdf (2006).

