# Supplementary material for: Transcriptome and metabolite analyses reveal the complex metabolic genes involved in volatile terpenoid biosynthesis in garden sage (Salvia officinalis)
Source: Sci Rep. 2017 Nov 22;7:16074. doi: 10.1038/s41598-017-15478-3 (PMC5700130; doi:10.1038/s41598-017-15478-3)
Supplement: Supplementary file 1 — Supplementary Tables and Figures [file 41598_2017_15478_MOESM1_ESM.pdf]

Transcriptome and metabolite analyses reveal the complex metabolic genes involved in the volatile terpenoid biosynthesis in garden sage (*Salvia officinalis*)

Mohammed Ali[1], Penghui Li[1], Guangbiao She[3], Daopu Chen[4], Xiaochun Wan[3], Jian Zhao[1]*

Supplementary Tables and Figures:

Table S1. The chemical composition of the essential oils of *S. officinalis*.

Table S2. Length distributions of transcripts and unigenes in *S. officinalis* transcriptome.

Table S3. Functional annotation of the *S. officinalis* transcriptome.

Table S4. KEGG classification based on secondary metabolism categories in *S. officinalis* trascriptome.

Table S5. Statistics of SSRs Identified from *S. officinalis* leaf transcriptome data.

Table S6. Summary of SSRs type in *S. officinalis* transcriptome.

Table S7. Length distribution of SSRs based on the number of repeat units.

Table S8. SSRs motifs that linked with unique sequences that involved in terpenoid biosynthesis.

Table S9. List of *S. officinalis* genes and primer pairs used for QRT-PCR.

Table S10. The major chemical composition and terpenes from transgenic *N. tabacum* leaves.

Table S11. List of *S. officinalis* genes and primer pairs used for full-Length Terpene Synthases cDNAs Clones.

Figure S1. Length distributions of transcripts and unigenes in *S. officinalis* trascriptome.

Figure S2. Blast NR similarity distribution in *S. officinalis* trascriptome.

Figure S3. Distribution of SSRs based on the motif units in *S. officinalis* transcriptome.

Figure S4. Typical GC-MS Peak of five terpene synthase genes from S. officinalis expression in transgenic *N. tabacum* leaves compares with wild type, 1- control (wild type of *N. tabacum*), 2-SoNEOD, 3-SoCINS, 4-SoSABS, 5- SoLINS, and 6-SoTPS6.

Table S1. The chemical composition of the essential oils of *S. officinalis*.

No.	Compounds	Retention time	Formula	M.W/Da	Terpene type	Young leaf	S.E, Old leaf	S.E, Stem	O.S.S
						% Peak	% Peak	% Peak	
1	Tricyclene	5.43	C10H16	136.234	Mono	0.01	0.08	-	
2	α-Thujene	5.57	C10H16	136.234	Mono	0.24	0.16	-	
3	Phellandrene	5.61	C10H16	136.234	Mono	-	-	0.24	
4	Linalyl propionate	5.68	C13H22O2	210.312	Mono	-	-	0.08	
5	(-)-α-Pinene	5.82	C10H16	136.234	Mono	2.14	1.96	0.35	SL, SA , SF, SC
6	Camphene	6.48	C10H16	136.234	Mono	0.54	2.17	0.15	S.L,S.A, S.F,S.C
7	(+)-Sabinene,	7.56	C10H16	136.234	Mono	0.37	0.16	-	S.L, S.A
8	Beta.-Pinene	7.76	C10H16	136.234	Mono	6.23	3.19	1.28	S.L,S.A, S.F, S.C
9	5-(Hydroxymethyl)spiro[2.4]heptan-5-ol	7.90	C8H14O2	142.195		-	-	0.04	
10	Beta.-Myrcene	8.55	C10H16	136.234	Mono	0.71	-	-	S.L,S.A, S.F
11	Lavandulol acetate	9.07	C12H20O2	196.286	Mono	0.01	-	-	
12	(+)-4-Carene	10.37	C10H16	136.23		0.05	-	-	S.L,S.A
13	β-Cymene	11.00	C10H14	134.218	Mono	0.03	-	-	S.F,S.C
14	1,8-Cineole	11.29	C10H18O	154.249	Mono	41.20	25.93	12.37	S.L,S.A, S.F
15	Gamma.-Terpinene	13.07	C10H16	136.23	Mono	0.06	-	-	S.L, S.A
16	4,4'-(3,3'-Dinitro-4,4'-biphenylylenebisazo)diphenol	13.16				0.04	-	-	
17	Cis-β-Terpineol	13.74	C10H18O	154.249	Mono	0.72	0.38	-	S.L, S.A
18	2-Carene	14.60	C10H16	136.23	Mono	0.03	-	-	S.L, S.A
19	4,7,7-Trimethylbicyclo[4.1.0] heptan-3-ol	14.69	C10H18O	154.249		-	-	-	
20	p-Menth-8-en-1-ol	15.47	C10H18O	154.249	Mono	0.07	0.15	-	
21	δ-Thujone	15.68	C10H16O	152.233	Mono	1.26	0.56	-	
22	Thujone	16.31	C10H16O	152.23	Mono	0.51	0.17	-	S.F
23	(-)-2-bornanone	17.69	C10H18O	154.2498	Mono	-	-	1.62	S.L, S.A , S.F, S.C
24	Camphor	17.7	C10H16O	152.23	Mono	6.27	11.52	-	S.L,S.A, S.F
25	Borneol	18.89	C10H18O	154.249	Mono	-	2.38	0.54	
26	α-Terpineol	18.92	C10H18O	154.249	Mono	1.34	-	-	
27	Terpinen-4-ol	19.33	C10H18O	154.249	Mono	0.06	0.16	-	S.L,S.A, S.C
28	Sabinene hydrate	19.58	C10H18O	154.249	Mono	0.01	-	-	S.F
29	β-Pinene epoxide	19.59	C10H16O	152.233	Mono	-	0.02	-	
30	2β-hydroxy-1,8-cineole	20.71	C10H18O2	170.248	Mono	0.19	0.14	-	

31	Methyl (2E,6Z)-2,6-dimethyl-8-oxo-2,6-octadienoate	20.83	C11H16O3	196.242		-	0.05	-	
32	Isobutyl carbonate	20.84	C9H18O3	174.24		0.12	-	-	
33	Isopregol	20.94	C10H18O	154.249	Mono	-	-	0.17	
34	Trans-2,7-Dimethyl-3,6-octadien-2-ol	21.04	C10H18O	154.249	Mono	-	0.01	-	
35	Linalyl 2-aminobenzoate	22.28	C17H23NO2	273.37		0.20	0.05	-	
36	9-Oxabicyclo[6.1.0]non-3-ene	22.55	C8H12O	124.180		0.01	-	-	
37	(E,Z)-α-Farnesene	22.59	C15H24	204.351	Sesqui	0.01	-	-	
38	Bornyl acetate	23.54	C17H24O4	196.286	Mono	0.24	1.40	0.51	S.F
39	3-Ketocamphor	24.46	C10H14O2	166.217	Mono	0.15	0.34	-	
40	Isocamphol	24.67	C10H18O	154.249	Mono	-	0.05	-	
41	α-Terpinenyl acetate	24.88	C12H20O2	196.286	Mono	4.23	1.26	1.52	
42	Exo-2-Hydroxycineole acetate	25.60	C12H20O3	212.285		0.22	0.05	-	
43	Alpha.-ylangene	25.73	C15H24	204.35	Sesqui	0.05	-	-	
44	7a-Methyl-3a,6,7,7a-tetrahydro-3H-benzofuran-2-one	25.73	C9H12O2	152.19		-	-	0.02	
45	cis-β-Terpineol	26.04	C10H18O	154.24	Mono	-	-	0.26	
46	α-Terpinyl isopentanoate	26.07	C15H26O2	238.36	Sesqui	-	-	0.24	
47	Glutaric acid	26.11	C20H28N2O8	424.44		-	-	0.48	
48	1-Hydroxymethyl-7,7-dimethy lbicyclo[2.2.1]heptan-2-one	26.14	C10H16O2	168.23		-	0.35	-	
49	Perhydronaphthalen-1-ol	26.14	C10H18O	154.24		-	-	0.29	
50	Spiro { 6,6-dimethyl-2,3-diazobicyclo [3.1.0] hex-2-ene-4,1'-cyclopropane }	26.22	C8H12N2	136.19		-	-	0.05	
51	α-Terpinenyl acetate	26.34	C12H20O2	196.28	Mono	0.01	0.11	-	
52	Tricyclo[5.3.0.0(3,9)]decane	26.36	C10H16	136.23		-	-	0.04	
53	Isoledene	26.50	C15H24	204.35	Sesqui	-	0.09	-	
54	Copaene	26.71	C15H24	204.35	Sesqui	0.02	-	-	
55	(-)-.Beta.-Bourbonene	26.98	C15H24	204.35	Sesqui	0.05	0.07	-	
56	Beta.-copaene	27.17	C15H24	204.35	Sesqui	0.02	0.01	-	
57	(E)-β-Elemene	27.25	C15H24	204.35	Sesqui	0.01	-	-	
58	5-Caranol	27.30	C10H18O	154.24	Mono	-	-	0.02	
59	α-Guajene	27.60	C15H24		Sesqui	0.01	-	-	
60	β-cis-Caryophyllene	27.70	C15H24	204.35	Sesqui	-	0.02	-	
61	α-Gurjunene	27.76	C15H24	204.35	Sesqui	0.03	0.02	-	S.L, S.A
62	Octahydro-1ah-indeno[1,2-b] oxirene	27.77	C9H14O	138.20		-	-	0.02	
63	Tricyclo[3.2.1.02,7]oct-3-ene, 2,3,4,5-tetramethyl-	27.90	C12H18	162.27		0.24	0.07	-	
64	(-)-Aristolene	27.91	C15H24	204.35	Sesqui	-	-	0.33	
65	Cedrene	27.99	C15H24	204.35	Sesqui	-	-	0.02	
66	Caryophyllene	28.20	C15H24	204.35	Sesqui	9.01	5.51	10.23	S.F
67	(E)-α-Bergamotene	28.45	C15H24	204.35	Sesqui	-	0.01	-	
68	(-)-.Alpha.-Panasinsen	28.67	C15H24	204.35	Sesqui	-	0.02	-	
69	7-Isopropenyl-1,4-dimethyl-1,2,3,3a,4,5,6,7-octahydroazulene	28.70	C15H24	204.35	Sesqui	0.02	-	-	

70	1H-Cycloprop[e]azulene, decahydro -1,1,7-trimethyl-4-methylene-	28.84	C15H24	204.35	Sesqui	0.08	0.25	-	
71	Bicyclo[3.2.0]heptan-2-one, 5-formylmethyl-6-hydroxy-3,3-dimethyl-6-vinyl-	28.95	C13H18O3	222.28		-	0.17	-	
72	Gallacetophenone	28.97	C8H8O4	168.1		0.03	-	-	
73	(+)-γ-Gurjunene	29.09	C15H24	204.35	Sesqui	-	0.04	-	
74	α-Caryophyllene	29.41	C15H24	204.35	Sesqui	2.22	3.72	7.30	S.L,S.A
75	1R,3Z,9s-4,11,11-Trimethyl-8-methylenebicycloundec-3-ene	29.538	C15H24	204.35	Sesqui	0.02	-	-	
76	5-Ketobornyl acetate	29.69	C12H18O3	210.26		-	0.02	-	
77	Naginata ketone	29.69	C10H12O2	164.20		-	-	0.31	
78	(-)-Germacrene D	30.09	C15H24	204.35	Sesqui	0.06	0.14	1.29	
79	Fumaric acid	30.43	C16H28O4	284.39		-	-	0.05	
80	α-Guajene	30.56	C15H24	204.35	Sesqui	-	0.05	-	
81	Guaia-1(10),11-diene	30.57	C15H24	204.35	Sesqui	0.02	-	-	

Table S1 (Continued)

No	Compound name	R.T	Formula	M.W/Da	Terpene type	Young leaf	Old leaf	Stem	O.S.S
						% Peak	% Peak	% Peak	
82	1,5,5-Trimethyl-6-methylene-cyclohexene	30.72	C10H16	136.23		-	0.03	-	
83	γ-Elemene	30.73	C15H24	204.35	Sesqui	0.80	-	0.36	
84	Longifolene-(V4)	30.87	C15H24	204.35	Sesqui	-	0.01	-	
85	8-Isopropenyl-1,5-dimethyl-cyclodeca-1,5-diene	31.12	C15H24	204.35	Sesqui	0.02	-	-	
86	Beta-ylangene	31.34	C15H24	204.35	Sesqui	-	0.03	-	
87	Selina-6-en-4-ol	31.35	C15H26O	222.36	Sesqui	-	-	0.03	
88	2,6,10,15,19,23-Hexamethyl-tetracosa-2,10,14,18,22-pentaene-6,7-diol	31.41	C30H52O2	444.73	triterpene	-	-	0.02	
89	Aromadendr-1-ene	31.48	C15H24	204.35	Sesqui	-	0.23	-	
90	cis-muurola-3,5-diene	31.48	C15H24	204.35	Sesqui	0.17	-	-	S.L,S.A
91	3-Isopropyl-4a,5-dimethyloctahydro-2(1H)-naphthalenone	32.33	C15H26O	222.36	Sesqui	0.01	-	-	
92	(+)-Viridiflorol	32.55	C15H26O	22236	Sesqui	0.01	-	-	S.L,S.A
93	Methyl (6E,9E,12E,15E)-6,9,12,15-docosatetraenoate	32.77	C23H38O2	346.54		-	-	0.01	
94	(-)-EPIGLOBULOL	32.88	C15H26O	222.36	Sesqui	-	0.01	-	
95	Ledene oxide	33.09	C15H24O	220.35	Sesqui	-	0.03	-	

96	6-Methyl-2-phenethyl[1,3] dioxan-4-one	33.18	C13H16O3	220.26		-	0.01	-	
97	1-Propene, 2-nitro-3-(1-cyclooctenyl)	33.26	C11H17NO2	195.25		-	-	0.09	
98	1H-Cycloprop[e] azulene, decahydro-1,1,7-trimethyl-4-methylene-	33.33	C15H24	204.35	Sesqui	-	0.24	-	
99	4-Epi-cubedol	33.33	C15H26O	222.36	Sesqui	0.15	-	-	
100	Caryophyllene oxide	33.42	C15H24	204.35	Sesqui	0.48	2.13	3.24	S.L,S.A, S.N
101	Docosahexaenoic acid	33.6	C22H32O2	328.48		-	-	0.07	
102	2-Methyl-5-octyn-4-yl dichloroacetate	33.63	C11H16Cl2O2	251.14		-	-	0.05	
103	(1R)-(-)-(10-CAMPHORSULF-ONYL)OXAZIRIDINE	33.83	C10H15NO3S	229.29	Mono	-	0.02	-	
104	Gleenol	33.93	C15H26O	222.36		0.02	-	-	
105	1,2-Dihydropyridine, 1-(1-oxobutyl)-	33.96	C9H13NO	151.20		-	0.07	-	
106	(-)-Ledol	34.14	C15H26O	222.36	Sesqui	0.03	0.01	-	S.L,S.A
107	Tricyclo[4.2.1.03,7] nonane-3,8-diol	34.15	C9H14O2	154.20		-	-	0.10	
108	α-Humulene epoxide II	34.29	C15H24O	220.35	Sesqui	0.03	0.65	-	
109	Cyclooctene, 1,2-dimethyl-	34.30	C10H18	138.24		-	-	0.50	
110	Ethanone, 1-(octahydro-1H-inden-1-yl)-,(1.alpha.,3a.alpha.,7a.beta.)-	34.36	C12H20 O	180.28		-	-	0.15	
111	Hexadecamethyl-cyclooctasioxane	34.47	C16H48O8Si8	593.23		-	-	0.14	
112	9,12-Octadecadien-1-ol, (Z,Z)-	34.92	C18H34O	266.46		-	0.01	-	
113	Beta-carotene	35.05	C20H28O	284.44	Diter	-	0.07	0.01	
114	Tetracyclo[6.3.2.0(2,5).0(1,8)]tridecan-9-ol, 4,4-dimethyl-	35.16	C15H24O	220.35		-	0.16	-	
115	Humulane-1,6-dien-3-ol	35.36	C15H26O	222.36	Sesqui	-	0.10	-	
116	9,10-Dimethyltricyclo[4.2.1.1(2,5)] decane-9,10-diol	35.18	C12H20O2	196.28		-	-	0.01	
117	3.beta.,4.alpha.,9.alpha.,11-Diepoxymuurolan-10-ol	36.04	C15H24O3	252.34		0.01	-	-	
118	Caryophyllene oxide	36.18	C15H24	204.35	Sesqui	-	0.22	-	
119	Bicyclo[4.4.0]dec-2-ene-4-ol, 2-methyl-9-(prop-1-en-3-ol-2-yl)-	36.21	C15H24O2	236.34	Diter	-	-	0.02	
120	(-)-Isolongifolol, acetate	36.29	C17H28O2	264.40		-	-	0.05	

Table S1 (Continued)

No	Compound name	R.T	Formula	M.W /Da	Terpene type	young leaf	old leaf	Stem	O.S .S
						% Peak	% Peak	% Peak	
121	8-hydroxygeraniol	36.74	$C_{10}H_{18}O_2$	170.24	Mono	-	0.35	-	
122	6-epi-shyobunol	36.77	$C_{15}H_{26}O$	222.36		0.08	-	-	
123	1-Isopropenyl-4-methyl-1,2-cyclohexanediol	37.09	$C_{10}H_{18}O_2$	170.24	Mono	-	0.03	-	
124	Diepicedrene-1-oxide	37.9	$C_{15}H_{24}O$	220.35		0.03	-	0.06	
125	Cyclononasiloxane, octadecamethyl-	38.63	$C_{18}H_{54}O_9$	667.38		-	-	0.09	
126	Androstalone	38.79	$C_{20}H_{32}O_2$	304.46	Diter	-	-	0.01	
127	β-Copaene	38.82	$C_{15}H_{24}$	204.35	Sesqui	0.07	-	-	
128	4-Hexyl-5-methyl-2-phenyl[1,3,2] dioxaborolane	38.89				0.02	-	-	
129	1-(7H-Purin-6-yl)proline	38.94	$C_{10}H_{11}N_5O_2$	233.22		0.03	-	-	
130	α-Bourbonene	39.1	$C_{15}H_{24}$	204.35	Sesqui	0.17	-	-	
131	11-Methylene-tricyclo[4.3.1.1(2,5)] undecane	39.10	$C_{12}H_{18}$	162.27		-	0.27	-	
132	(1E)-1-Ethylideneoctahydro-1H-indene	39.16	$C_{11}H_{18}$	150.26		-	-	0.27	
133	1-Butoxy-1-chlorosilolane	39.24	$C_8H_{17}ClO$	192.75		-	-	0.06	
134	cis-7-Ethyl-bicyclo[4.3.0]non-3-ene	39.34	$C_{11}H_{18}$	150.26		-	0.01	-	
135	Carbamic acid	39.45	$C_{10}H_{13}NO_2$	179.21		0.01	-	-	
136	Geranyl-α-terpinene	39.88	$C_{20}H_{32}$	272.46	Diter	0.06	0.03	-	S.L, S.A , ,S. F
137	cis-Phytol	39.97	$C_{20}H_{40}O$		Diter	-	0.02	-	
138	2-Methylenecholestan-3-ol	40.17	$C_{28}H_{48}O$	400.68		-	0.02	-	
139	Andrographolid	40.27	$C_{20}H_{30}O_5$	350.44		0.01	-	-	
140	12-Oxatetracyclo[4.3.1.1(2,5).1(4,10)] dodecane, 11-isopropylidene-	40.51	$C_{14}H_{20}O$	204.30		-	0.07	-	
141	Bicylo[4.1.0]heptane, 7-bicyclo[4.1.0] hept-7-ylidene-	40.53	$C_{14}H_{20}$	188.30		0.01	-	-	
142	Isoaromadendrene epoxide	40.79	$C_{15}H_{24}O$	220.35	Sesqui	0.4	-	0.08	S.L, S.A
143	Bicyclo[4.4.0]dec-2-ene-4-ol, 2-methyl-9-(prop-1-en-3-ol-2-yl)-	40.95	$C_{15}H_{24}O_2$	236.34		-	0.02	-	
144	Strophanthidol	40.95	$C_{23}H_{34}O_6$	406.51		0.01	-	-	
145	Beta-ylangene	41.45	$C_{15}H_{24}$	204.35	Sesqui	0.96	0.36	-	
146	10,12-Pentacosadiynoic Acid	41.54	$C_{25}H_{42}O_2$	374.59		-	-	0.14	
147	2-Propen-1-ol, 3-(2,6,6-trimethyl-1-cyclohexen-1-yl)-	41.57	$C_{12}H_{20}O$	180.28		-	-	0.27	
148	4-Methoxyphenyl methyl[4-(1-pyrrolidinyl)-2-butyn-1-yl]carbamate	41.72	$C_{17}H_{22}N_2O_3$	302.36		-	-	0.03	
149	Biformene	42.35	$C_{20}H_{32}$	272.46	Diter	0.17	0.25	-	S.L, S.A

150	Verticillol	42.38	C20H34O	290.48	Diter	-	-	0.43	
151	Kaur-16-en-18-yl acetate	42.79	C22H34O2	330.50		0.02	0.06	-	
152	10,12,14-Nonacosatriynoic acid	42.90				-	0.07	-	
153	6,9-Octadecadiynoic acid, methyl ester	42.90	C19H30O2	290.44		0.04	-	-	
154	Cedran-diol, 8S,13-	43.23	C15H26O2	238.36	Sesqui	0.01	-	-	
155	(+)-Beyerene	43.33	C20H32	272.46	Diter	0.01	0.01	-	
156	Androstan-17-one, 3-ethyl-3-hydroxy-, (5.alpha.)-	43.35	C21H34O2	318.49		0.01	0.06	-	
157	Z,Z,Z-1,4,6,9-Nonadecatetraene	43.46	C19H32	260.45		-	-	0.01	
158	Cycloeucalenone	43.55	C30H48 O			-	0.01	-	
159	trans-γ-Caryophyllene	43.82	C15H24	204.35	Sesqui	0.02	0.03	-	S.F
160	Androteston	44.1	C22H32O3	344.48		0.02	-	-	

Table S1 (Continued)

No	Compound name	R.T	Formula	M.W /Da	Terpene type	Young leaf	Old leaf	Stem	O.S.S
						% Peak	% Peak	% Peak	
161	Alloaromadendrene oxid	44.60	C15H24O	220.35	Sesqui	0.03	-	-	S.L, S.A
162	14-Methylcholest-8-en-3-yl acetate	44.63	C30H50O2	442.71		-	-	0.02	
163	Cycloheptane, 4-methylene-1-methyl-2-(2-methyl-1-propen-1-yl)-1-vinyl-	44.80	C15H24	204.35	Sesqui	-	0.10	-	
164	β.-Ionon-5,6-epoxide	44.84	C13H20O2	208.29		-	-	0.27	
165	Stearaldehyde	45.33	C18H36O	268.47		0.01	0.03	-	S.L, S.A
166	Cyclooctasiloxane, hexadecamethyl-	45.69	C16H48O8Si8	593.23		-	-	0.12	
167	Epimanool	45.80	C20H34O	290.48	Diter	-	2.07	2.10	
168	Thunbergol	45.83	C20H34O	290.48		0.17	-	-	
169	Eudesm-11-en-1-ol	46.51	C15H26O	222.36		-	0.54	-	
170	(-)-Isolongifolol, methyl ether	46.56				0.01	-	-	
171	Androst-5,7-dien-3-ol-17-one	46.66	C19H26O2	286.40		-	-	0.01	
172	Linoleic acid ethyl ester	46.87	C20H36O2	308.49		0.04	-	-	
173	Methyl 8,11,14-heptadecatrienoate	46.99	C18H30O2	278.42		-	0.01	-	
174	Methyl 7,10,13-hexadecatrienoate	47.01	C17H28O2	264.40		0.07	-	-	
175	Trans-Phytol	47.23	C20H40O	296.53	Diter	-	0.05	-	
176	(5E)-4,9-Dihydroxy-6-methyl-3,10-bis(methylene)-3a,4,7,8,9,10,11,11a-octahydrocyclodeca[b]furan-2(3H)-one	47.26	C15H20O4	264.31		0.07	-	-	
177	12-Hydroxypregnan-20-one	47.62	C21H34O2	318.49		-	0.02	-	
178	O-Trimethylsilyl cholesterol	47.625	30H54OSi	458.83		0.01	-	-	
179	(R)-(-)-14-Methyl-8-hexadecyn-1-ol	47.77	C17H32O	252.43		-	0.01	-	
180	Isoborneol	47.91	C10H18O	154.24	Mono	0.01	-	-	S.L,S.A ,S.F,S.C
181	N-Methyl-pseudotomatidine diacetate	47.99	C32H51NO4	513.75		-	0.01	-	
182	2-Methylenecholestan-3-ol	48.40	C28H48O	400.68		-	-	0.06	

183	2-(Cholest-5-en-3-yloxy)ethyl acetate	48.54	C31H52O3	472.74		0.01	-	-	
184	31,32 Dioxapentacyclo[20.8.1.17,16. 01, 22.07,16]dot	48.74	C30H52O2	444.73		-	0.15	-	
185	Cyclononasiloxane	48.76	C18H54O9S i9	667.38		-	-	0.40	
186	Ergost-25-ene-3,5,6-triol	48.90	C28H48O3	432.67		-	0.02	-	
187	Tetracosane, 2-methyl	49.14	C25H52	352.68		-	0.01	-	
188	Murolan-3,9(11)-diene-10-peroxy	49.59	C15H24O2	236.34		0.01	-	-	
189	2H-1-benzopyran-2-one, 8-methoxy-3-(1H-naphth[2,3-d]imidazol-2-yl)-	49.74	C21H14N2 O3	342.34		0.04	-	-	
190	2',5'-Dimethoxy-4,6-bis(2-methyl-2-propanyl)-2-biphenylol	49.86	C22H30O3	342.47		1.00	1.67	-	
191	Estra-1,3,5(10)-trien-16-one, 3-[(trimethylsilyl)oxy]-	49.87	C22H32O2S i	356.57	Diter	0.18	-	1.07	
192	Cycloheptane, 4-methylene-1-methyl-2-(2-methyl-1-propen-1-yl)-1-vinyl-	50.28	C15H24	204.35		0.03	-	-	
193	Methyl 3-acetamido-5-methyl-1-(2-oxopropyl)-1H-indole-2-carboxylate	50.63	C16H18N2 O4	302.32		-	0.41	-	
194	Beyeran-18-oic acid	50.64	C20H32O2	304.46		0.09	-	-	
195	4,5,6,7-Tetrahydroxy-1,8,8,9-tetramethyl-8,9-dihydrophenaleno[1,2-b]furan-3-one	50.78	C19H18O6	342.34		-	-	0.87	
196	4,14-Dimethyl-9,19-cycloergost-24(28)-en-3-yl acetate	50.85	C32H52O2	468.75		0.63	0.83	-	
197	Cupressene	50.89	C20H32	272.46		-	-	0.91	
198	13,17-Seco-5.alpha.-pregn-13(18)-en-20-one	50.92	C21H34O	302.49		-	-	2.16	
199	Humulane-1,6-dien-3-ol	50.99	C15H26O	222.36	Sesqui	0.87	0.91	-	
200	1-[3,3-Dimethyl-2-(3-methyl-buta-1,3-dienyl)-cyclopentyl]-2-hydroxy-ethanone	51.13	C14H22O2	222.32		-	-	0.39	

Table S1 (Continued)

No	Compound name	R.T	Formula	M.W/Da	Terpene type	Young leaf	Old leaf	Stem	O.S.S
						% Peak	% Peak	% Peak	
201	4,4'-Di-tert-butyl-o,o'-biphenol	51.31	C20H26O2	298.41		0.14	-	-	
202	Trimethylsilyl ether of estriol, Oestriol, tris-TMS	51.60	C27H48O3Si3	504.92		0.48	0.91	-	
203	Norcaradiene, 2,3,4,5-tetramethyl-7,7-diphenyl-	51.77	C23H24	300.43		0.31	0.80	-	
204	Trans-Ferruginol	52.11	C20H30O	286.45	Diter	0.51	0.63	-	
205	Estra-1,3,5(10)-trien-16-one, 3-[(trimethylsilyl)oxy]-	52.21	C25H35NO7Si	489.63	Diter	0.42	-	1.75	
206	Androst-5-en-4-one	51.24	C19H28O	272.42		-	0.12	-	
207	12-Hydroxyabieta-1,8(14),9(11),12-tetraen-3-one	51.3	C20H26O2	298.41		-	0.34	-	
208	D-Homopregnan-20-one, (5.alpha.)-	52.03	C22H36O	316.52		-	0.36	-	
209	Silane, [[(16.beta.,17.beta.)-16,17-epoxyestra-1,3,5(10)-trien-3-yl]oxy]trimethyl-	52.22	C21H30O2Si	342.54	Diter	-	0.76	3.66	
210	Octadeca-3,13-dien-1-ol	52.34	C18H34O	266.46		-	-	1.67	

211	Sugiol	53.07	C20H28O2	300.43	Diter	0.47	10.80	15.89	
212	(3,3-dimethyl-2,3-dihydro-1H-benzo[f]chromen-8-yloxy) trimethylsilane	53.08	C18H24O2Si	300.46		-	-	1.48	
213	(-)-Globulol	53.72	C15H26O	222.36	Sesqui	0.01	-	-	S.L,S.A
214	4,14-Dimethyl-9,19-cycloergost-24(28)-en-3-yl acetate	53.87	C32H52O2	468.75		0.09	-	-	
215	Estra-1,3,5(10),9(11)-tetraen-17-one, 3-[(trimethylsilyl)oxy]-	54.43	C21H28O2Si	340.53		-	-	1.33	
216	Phenol, 2,6-bis(1,1-dimethylethyl)-4-[(4-hydroxy-3,5-dimethylphenyl) methyl]-	54.45	C23H32O2	340.49		0.07	-	-	
217	Colchicine, 7-deacetoamino-5,6-dedihydro-	54.68	C20H20O5	340.36		0.05	-	-	
218	9-(4-Butyl-benzyl)-acridine	55.27	C24H23N	325.44		-	0.13	-	
219	Acetamide, N-(5,6,7,9-tetrahydro-2-hydroxy-1,3,10-trimethoxy-9-oxobenzo(a)heptalen-7-yl)-, (S)-	55.72	C21H23NO6	385.41		-	0.37	-	
220	Benzene, 1-(4-phenyl-1,3-butadiynyl)-3-[2-(trimethylsilyl)ethynyl]-	55.73	C21H18Si	298.45		0.06	-	-	
221	Phen-1,4-diol, 2,3-dimethyl-5-trifluoromethyl-	59.40	C9H9F3O2	206.16		-	-	0.09	
222	(+)-Totarol,	60.84	C20H30O	286.45	Diter	-	0.13	-	
223	Dibenz[d,f]cycloheptanone, 2,3,9-trimethoxy-	63.11	C18H18O4	298.33		-	0.18	-	
224	N-Eicosane	68.32	C20H42	282.54	Diter	-	0.12	-	
225	Heneicosane	68.33	C21H44	296.57		0.34	-	-	S.L,S.A,S.N
226	1-Octadecanesulphonyl chloride	68.42	C18H37ClO2S	353.00		-	-	0.49	
227	Methyl 2-[(2Z)-1-hydroxy-2-buten-2-yl]-1,2,6,7,12,12b-hexahydroindolo [2,3-a]quinolizine-3-carboxylate	68.46	C21H24N2O3	352.42		-	-	0.13	
228	d-Mannitol, 1-decylsulfonyl-	68.64	C16H34O7S	370.50		-	-	0.04	
229	12-hydroxyabieta-8,11,13-trien-7-one	71.01	C20H28O2	300.43		0.11	-	-	
230	4,8,12-Tetradecatrien-1-ol, 5,9,13-trimethyl-	74.17	C17H30O	250.41		-	-	2.11	
231	Docosa-2,6,10,14,18-pentaen-22-al, 2,6,10,15,18-pentamethyl-, all-trans	74.11	C27H44O	384.63		0.10	-	-	
232	Squalene	74.12	C30H50	410.71	Triter	-	0.16	-	
233	E,E,Z-1,3,12-Nonadecatriene-5,14-diol	74.22	C19H34O2	294.47		-	-	1.43	
234	Pregnan-20-one, 3-hydroxy-	75.06	C21H34O2	318.49		-	-	0.04	
235	25,26-dihydroxycholecalciferol	78.17	C27H44O3	416.63		-	-	0.02	
236	2-methyloctacosane	79.94	C29H60	408.78		0.12	0.23	0.65	
	Total					89.29	91.54	85.27	

R.T: Retention Time O.S.S: Other salvia species SA: *Salvia acetabulosa* S.L: *Salvia leriifolia*
S.F: *Salvia fruticosa* S.N: *Salvia nemorosa* S.C: *Salvia compressa*

Table S2. Length distributions of transcripts and unigenes in *S. officinalis* transcriptome.

Nucleotide length (bp)	Number of transcripts	Number of Unigenes
200-500bp	34.051 (38.45 %)	27.381 (56.26 %)
500-1kbp	17.658 (19.94 %)	8.576 (17.62 %)
1k-2kbp	22.529 (25.44%)	8.068 (16.58 %)
>2kbp	14.316 (16.17%)	4.646 (9.54 %)
Total	88.554	48.671
Min Length (bp)	201	201
Mean Length (bp)	1.113	813
Median Length (bp)	758	417
Max Length (bp)	14.571	14.571
N50 (bp)	1.793	1.485
N90 (bp)	479	298
Total Nucleotides length (bp)	98.521.170	39.579.914

Table S3. Functional annotation of the *S. officinalis* transcriptome.

Annotation database	No. of unigene hits	Percentage %
Annotated in NR	28.413	58.37
Annotated in NT	12.032	24.72
Annotated in KO	9.716	19.96
Annotated in Swiss-Prot	21.264	43.68
Annotated in PFAM	20.439	41.99
Annotated in GO	22.891	47.03
Annotated in KOG	11.952	24.55
Annotated in all Databases	4.259	8.75
Annotated in at least one Database	30.308	62.27
Total Unigenes	48.671	100

Table S4. KEGG classification based on secondary metabolism categories in *S. officinalis* trascriptome.

N	Pathway Hierarchy	KEGG Pathway	Pathway ID	Gene Number
1	Metabolism of Terpenoids and Polyketides	Terpenoid backbone biosynthesis	ko00900	70
2	Metabolism of Terpenoids and Polyketides	Monoterpenoid biosynthesis	ko00902	20
3	Metabolism of Terpenoids and Polyketides	Sesquiterpenoid and triterpenoid biosynthesis	ko00909	14
4	Metabolism of Terpenoids and Polyketides	Diterpenoid biosynthesis	ko00904	30
5	Metabolism of Terpenoids and Polyketides	Limonene and pinene degradation	ko00903	28
6	Metabolism of Terpenoids and Polyketides	Geraniol degradation	ko00281	1
7	Metabolism of Terpenoids and Polyketides	Carotenoid biosynthesis	ko00906	45
8	Metabolism of Terpenoids and Polyketides	Polyketide sugar unit biosynthesis	ko00523	1
9	Metabolism of Terpenoids and Polyketides	Tetracycline biosynthesis	ko00253	10
10	Metabolism of Terpenoids and Polyketides	Zeatin biosynthesis	ko00908	24
11	Metabolism of Terpenoids and Polyketides	Biosynthesis of ansamycins	ko01051	18
12	Metabolism of Terpenoids and Polyketides	Brassinosteroid biosynthesis	ko00905	8
13	Metabolism of Terpenoids and Polyketides	Biosynthesis of siderophore group nonribosomal peptides	ko01053	1
14	Biosynthesis of other secondary metabolites	Streptomycin biosynthesis	ko00521	22
15	Biosynthesis of other secondary metabolites	Stilbenoid, diarylheptanoid and gingerol biosynthesis	ko00945	39
16	Biosynthesis of other secondary metabolites	Phenylpropanoid biosynthesis	ko00940	124
17	Biosynthesis of other secondary metabolites	Flavone and flavonol biosynthesis	ko00944	8
18	Biosynthesis of other secondary metabolites	Flavonoid biosynthesis	ko00941	34
19	Biosynthesis of other secondary metabolites	Glucosinolate biosynthesis	ko00966	10
20	Biosynthesis of other secondary metabolites	Isoquinoline alkaloid biosynthesis	ko00950	31
21	Biosynthesis of other secondary metabolites	Novobiocin biosynthesis	ko00401	8
22	Biosynthesis of other secondary metabolites	Tropane, piperidine and pyridine alkaloid biosynthesis	ko00960	32
23	Biosynthesis of other secondary metabolites	Caffeine metabolism	ko00232	5
24	Biosynthesis of other secondary metabolites	Butirosin and neomycin biosynthesis	ko00524	11
25	Biosynthesis of other secondary metabolites	Anthocyanin biosynthesis	ko00942	1
26	Biosynthesis of other secondary metabolites	Betalain biosynthesis	ko00965	4
27	Biosynthesis of other secondary metabolites	Aflatoxin biosynthesis	ko00254	9

Table S5. Statistics of SSRs identified from *S. officinalis* leaves transcriptome data.

Items	Characteristics
Total number of sequences examined	48.671
Total size of examined sequences (bp)	39.579.914
Total number of identified SSRs	9.149
Number of SSR containing sequences	7.439
Number of sequences containing more than 1 SSR	1.391
Number of SSRs present in compound formation	583

Table S6. Summary of SSRs in *S. officinalis* transcriptome.

**Repeat motif**	**Number** [a]	**Percentage**[b]
Mon- nucleotide		
A/T	2272	
C/G	76	
Total	2348	24.13
Di- nucleotide		
AC/GT	918	
AG/CT	2999	
AT/AT	369	
CG/CG	9	
Total	4295	44.132
Tri- nucleotide		
AAC/GTT/AAG/CTT	609	
AAT/ATT/ACC/GGT	333	
ACG/CGT/ACT/AGT	62	
AGC/CTG/AGG/CCT	586	
ATC/ATG/CCG/CGG	727	
Total	2317	23.81
Tetra- nucleotide		
AAAC/GTTT/AAAG/CTTT/AAAT/ATTT	51	
AACC/GGTT/AACT/AGTT/AAGC/CTTG	10	
AAGG/CCTT/AATC/ATTG/AATG/ATTC	15	
AATT/AATT/ACAT/ATGT/ACGC/CGTG	15	
ACTC/AGTG/ACTG/AGTC/AGAT/ATCT	15	
AGCT/AGCT/AGGC/CCTG/AGGG/CCCT/ATCC/ATGG	10	
Total	116	1.191
Penta- nucleotide		
AAAAC/GTTTT/AAAAG/CTTTT/AAAAT/ATTTT/AAACC/GGTTT	4	
AAATC/ATTTG/AAATT/AATTT/AACAC/GTGTT/AACCC/GGGTT	4	
AACGT/ACGTT/AAGAG/CTCTT/AAGCT/AGCTT/AAGGG/CCCTT	4	
AATAC/ATTGT/AATAG/ATTCT/AATCG/ATTCG/AATGC/ATTGC	4	
AATTC/AATTG/ACAGT/ACTGT/ACATC/ATGTG/ACCCC/GGGGT	4	
ACCGC/CGGTG/ACGCC/CGTGG/ACTGG/AGTCC/AGAGG/CCTCT	7	
AGATG/ATCTC/AGCAT/ATGCT/AGGGG/CCCCT/ATATC/ATATG/CCCGG/CCGGG	7	
Total	34	0.35
Hexa- nucleotide		
AAAATG/ATTTTC/AAATAG/ATTTCT/AAATTC/AATTTG/AACAAT/ATTGTT	4	
AACACC/GGTGTT/AACACG/CGTGTT/AACTAC/AGTTGT/AACTCC/AGTTGG	4	
AACTCG/AGTTCG/AACTGC/AGTTGC/AAGAGC/CTCTTG/AAGAGG/CCTCTT	4	
AAGCCC/CTTGGG/AAGGAG/CCTTCT/AATACC/ATTGGT/AATAGT/ACTATT	4	
AATATC/ATATTG/AATATG/ATATTC/AATCAC/ATTGTG/AATCTG/AGATTC	4	
AATGAT/ATCATT/AATGGC/ATTGCC/AATTAG/AATTCT/ACACCC/GGGTGT	4	
ACACCG/CGGTGT/ACATAT/ATATGT/ACCCGC/CGGGTG/ACGTCC/ACGTGG	4	
ACTCTG/AGAGTC/ACTGAT/AGTATC/AGAGGC/CCTCTG/AGATAT/ATATCT	4	
AGATGG/ATCTCC/AGCCGC/CGGCTG/AGCGGC/CCGCTG/AGCGGG/CCCGCT	4	
AGGCCG/CCTCGG/AGGCGG/CCGCCT/ATCCCG/ATCGGG	3	
Total	39	0.4
compound SSRs	583	5.99
sum	9732	100.003

[a] Number of total SSRs detected in unigenes, [b] percentage of total SSRs with different repeat motifs.

Table S7. Length distribution of SSRs based on the number of repeat units.

Number of repeat units	Mon-	Di-	Tri-	Tetra-	Penta-	Hexa-	Total	Percentage
5	0	0	1281	88	29	13	1411	15.43
6	0	1388	577	23	1	10	1999	21.86
7	0	878	420	0	1	2	1301	14.23
8	0	697	35	0	0	5	737	8.06
9	0	650	1	1	1	2	655	7.16
10	967	522	0	0	0	1	1490	16.30
11	421	152	0	0	0	3	576	6.30
12	228	6	1	0	1	0	236	2.58
13	135	0	0	0	0	0	135	1.48
14	103	0	0	0	0	0	103	1.13
15	87	0	0	0	0	0	87	0.95
16	52	0	1	0	0	1	54	0.59
17	38	0	0	0	0	0	38	0.42
18	41	0	0	0	0	0	41	0.45
19	57	0	0	0	1	0	58	0.64
20	78	0	0	0	0	0	78	0.85
21	80	0	0	0	0	0	80	0.87
22	36	0	0	0	0	0	36	0.40
23	21	0	0	0	0	0	21	0.22
≥24	4	2	1	0	0	0	7	0.08
							9143	

Table S8. SSRs motifs that linked with unique sequences that involved in terpenoid biosynthesis.

Pathway	Gene name	Kegg Entry	Unigene ID	SSR nr.	SSR Type	SSR	Size	Start	End
MEP	DXS 4	K01662	*So*|comp10248_c0	1	c	(TC)9tacgatctctttcttggagtgaagctgcac ggcagagaatgaaagagtattaat(GA)7	86	2479	2564
	DXS5	K01662	*So* |comp28480_c0	1	p2	(CA)6	12	122	133
	HDR2	K03527	*So* |comp26756_c1	1	p4	(CATT)5	20	1	20
MVA	HMGS	K01641	*So* |comp10117_c0	1	p2	(GA)6	12	1742	1753
	HMGR3	K00021	*So* |comp17290_c0	1	p3	(CGC)5	15	107	121
Other.Tepene	FLDH	K15891	*So* |comp24181_c1	1	p2	(TC)10	20	1258	1277
	PCYOX1	K05906	*So* |comp17568_c0	1	p2	(TC)6	12	418	429
	FNTA	K05955	*So* |comp21306_c0	1	p1	(A)10	10	2	11
	DHDDS 1	K11778	*So* |comp24971_c0	1	p2	(TC)6	12	179	190
	DHDDS 5	K11778	*So* |comp16046_c0	1	p1	(A)10	10	44	53
Sesquiterpene	Farnesol dehydrogenase (FARD)	K15891	*So* |comp24181_c1	1	p2	(TC)10	20	1258	1277
Diterpene	Momilactone-A synthase	K13070	*So* comp21612_c0	1	p1	(T)10	10	1283	1292
	GGPSII7	K13789	*So* |comp107254_c0	1	p3	(ACC)6	18	1	18
	GGPSII10	K13789	*So* |comp28724_c0	1	p2	(GA)7	14	1502	1515
	Ent-copalyl diphosphate synthase	K04120	*So* |comp23218_c0	1	p3	(CCG)5	15	3240	3254
	Ent-kaurenoic acid hydroxylase	K04123	*So* comp15654_c0	1	p2	(GA)6	12	1906	1917
Triterpene	Beta-amyrin synthase	K15813	*So* |comp27006_c0	1	p2	(AT)6	12	2830	2841
	Squalene monooxygenase	K00511	*So* |comp24504_c0	1	p2	(AG)6	12	302	313

Table S9. List of *S. officinalis* genes and primer pairs used for QRT-PCR and Semiquantitative RT-PCR.

Gene	Primer name	Primer sequence	PCR product (bp)
SoACTIN	SoACTIN -F	5′- GGCAGTTCTCTCCCTCTAT-3′	157
	SoACTIN-R	5′- GAGGTGGTCGGTGAGAT-3′	
SoNEOD	SoNEOD -F	5′- GTCAATGTCTCCTCCACTTTAG -3′	153
	SoNEOD -R	5′- CTCTTGCAGTTTACCCTCTTT-3′	
SoGPS	SoGPS-F	5′- CTGGACAAACGGCAGAAG -3′	150
	SoGPS-R	5′- CAATCCCGTGGCGAATATC -3′	
SoFPPS2	SoFPPS2-F	5′- CTCTCGGCTGGTGTATTG -3′	159
	SoFPPS2-R	5′- GGATATGGTTCCGGAGAATG -3′	
SoCINS	SoCINS-F	5′- GGTGTTGCAGGAAGAAGTAG -3′	161
	SoCINS-R	5′- CTGTTGAGTACAGATCCCTTTC -3′	
So SABS	SoSABS -F	5′- CAACGCCAAAGTTTCGATATCC-3′	150
	SoSABS -R	5′- GCAAGCCTTAAAATCATTCCCG-3′	
SoLINS	SoLINS -F	5′- AGAATTGGTGAAGGCAGAGG-3′	155
	SoLINS -R	5′- GTAGGATGTGGGTCTGATTGG-3′	
SoTPS6	SoTPS6-F	5′- TGAGGATACACTTCAAAGCCC-3′	158
	SoTPS6-R	5′- GTACATCTCAGCCATCCTTATCAT-3′	
NtEF-1α	NtEF-1α-F	5′- TGGTTGTGACTTTTGGTCCCA-3′	160
	NtEF-1α-R	5′- ACAAACCCACGCTTGAGATCC-3′	
SoMYRC	SoMYRC-F	CACTGCACACGCTATGAA	160
	SoMYRC-R	GTGCTACATGAACGACCATA	
SoGGPP	SoGGPP-F	GCGGAGATTCTTGATGAGTG	154
	SoGGPP-R	CCGAAATTCCTGAGCTTCTC	
*So*HUMS	*So*HUMS-F	GGATGTGTGTAGCCATCTTG	158
	*So*HUMS-R	CAAGAGGATGGCTGAGAATG	
*So*SQUS	*So*SQUS-F	GTATCTCTGTGCTGCTGATG	155
	*So*SQUS-R	GCTCTCTCTGTTCTCCTGA	

Table S 10. The major chemical composition and terpenes from transgenic *N. tabacum* leaves

N	compound name	R.T	Terpene Type	***W.T***	***SoNEOD***	***SoCINS***	***SoSABS***	***SoLINS***	***SoTPS6***
1	Cyclotetrasiloxane, octamethyl-	7.657		–	0.03	–	–	–	–
2	Dodecane	15.526		–	–	0.06	–	–	–
3	Cyclohexanol, 3,5-dimethyl-	16.003		–	0.01	–	–	–	–
4	1-Methylcycloheptanol	16.079		0.01	–	–	–	–	–
5	Cyclopentasiloxane, decamethyl-	16.938		–	0.02	–	–	–	–
6	L-.alpha.-Terpineol	20.607	Mono	–	–	0.05	–	–	–
7	Cyclohexasiloxane, dodecamethyl-	24.067			0.01	0.03	–	–	–
8	Pyridine, 3-(1-methyl-2-pyrrolidinyl)-, (S)-	26.372		22.49	0.08	–	0.71	–	0.35
9	Pentasiloxane, dodecamethyl-	29.592		–	–	–	–	–	0.18
10	Cycloheptasiloxane, tetradecamethyl-	29.614		–	0.07	–	–	0.19	–
11	Cyclohexasiloxane, dodecamethyl-	29.703		–	–	0.19	–	–	–
12	Cycloheptasiloxane, tetradecamethyl-	30.199		–	–	0.12	–	–	–
13	trans-.beta.-Ionone	30.596		–	–	0.05	0.17	–	–
14	3-Buten-2-one, 4-(2,6,6-trimethyl-1-cyclohexen-1-yl)-	30.615		–	0.03	–	–	0.05	–
15	3-tert-Butyl-4-hydroxyanisole	30.727		–	–	0.07	–	–	–
16	Topanol;Stavox	31.146	Sesqui	–	0.04	–	–	0.04	–
17	2(4H)-Benzofuranone, 5,6,7,7a-tetrahydro-4,4,7a-trimethyl-, (R)-	32.314		–	–	0.04	0.21	–	–
18	1,2,4-Cyclopentanetrione, 3-(2-pentenyl)-	34.585		–	–	0.8	–	–	–
19	Cyclooctasiloxane, hexadecamethyl-	35.167		–	0.09	0.05	–	0.8	0.35
20	cis-Carveol	35.519	Mono	–	–	–	–	0.1	–
21	.alpha.-Campholenal	35.809	Mono	–	0.08	–	0.23	–	–
22	Menthofuran;	36.17	Mono	–	–	0.05	–	–	–
23	Benzofuran, 4,5,6,7-tetrahydro-3,6-dimethyl-	37.186		–	–	0.06		–	–
24	Tetradecanal	37.606		–	–	–	0.23	–	–
25	Nonadecane	37.821		–	–	0.11	–	–	–
26	Cyclohexasiloxane, dodecamethyl-	38.678		–	0.16	0	–	1.14	0.61

27	Oxirane, tetradecyl-	38.766		–	–	1.26	–	–	–
28	Tetradecanoic acid	39.223		–	0.33	–	–	–	–
29	n-Hexadecanoic acid	39.421		–	–	–	0.17	–	–
30	Octadecane	39.649		–	0.12	–	–	–	–
31	Cyclononasiloxane, octadecamethyl-	39.698		–	–	0.33	–	–	–
32	Tetratetracontane	39.71		–	–	–	–	0.07	–
33	Oxirane, tetradecyl-	40.561		–	–	–	0.94	–	–
34	1-Octadecyne	40.599		0.08	5.19	–	–	0.08	–
35	trans-11-Tetradecenyl acetate	40.648		–	–	–	–	–	0.1
36	Pentadecanoic acid	40.853		–	–	1.3	–	–	–
37	Pentadecanal-	41.094		–	–	0.14	–	–	–
38	Oxirane, tetradecyl-	41.216		–	0.17	–	–	–	–
39	1,2-Benzenedicarboxylic acid, mono(2-ethylhexyl) ester	41.45		–	–	–	–	0.05	–
40	1-Octadecyne	41.673		–	0.26	–	–	–	–
41	2-Pentadecanone, 6,10,14-trimethyl-	41.681		–	–	0.06	–	–	–
42	9,12,15-Octadecatrienoic acid, (Z,Z,Z)-	42.204		–	–		1.45		–
43	9,12,15-Octadecatrienoic acid, methyl ester, (Z,Z,Z)-	42.22		–	0.38	–	–	–	–
44	Cyclohexasiloxane, dodecamethyl-	42.369		–	0.3	–	–	–	0.87
45	α-Linolenic acid	42.37		0.19	–	–	–	–	–
46	Heptasiloxane, hexadecamethyl-	42.383		–	–	–	–	1.24	–
47	Hexadecanoic acid, methyl ester	42.969		–	0.19		–	–	–
48	1,2-Benzenedicarboxylic acid, bis(2-methylpropyl) ester	42.473		–	–	1.77	–	–	–
49	Hexadecanoic acid, methyl ester	43.019		–	–	–	–	0.07	–
50	Cyclononasiloxane, octadecamethyl-	43.039		–	–	0.36	–	–	–
51	7-Hexadecenal, (Z)-	43.26		–	–	0.32	–	–	–
52	4,7,10,13,16,19-Docosahexaenoic acid, methyl ester, (all-Z)-	43.378		–	0.26		–	–	–
53	9,12,15-Octadecatrienoic acid, methyl ester, (Z,Z,Z)-	43.709		–	–	–	2	0.78	–
54	Hexadecanoic acid, methyl ester	44.165			1.66	5.41	–	–	–
55	n-Hexadecanoic acid	44.301		3.77	6.74	0.37	8.72	5.43	2.09
56	9,12,15-Octadecatrienoic acid, (Z,Z,Z)-	44.855		–	–	18.57	–	–	–
57	α-Bulnesene	45.066	Sesqui	–	–		0.22	–	–
58	cis-9-Hexadecenal	45.266		–	–	–	0.33	–	–

59	4,8,13-Cyclotetradecatriene-1,3-diol, 1,5,9-trimethyl-12-(1-methylethyl)-	45.63		0.38	–	–	–	–	–
60	Triadimefon	45.59		–	–	0.32	–	–	–

Table S10.

N	compound name	R.T	Terpene Type	W.T	***SoNE OD***	***SoCI NS***	***SoSA BS***	***SoLI NS***	***SoTP S6***
61	4,8,13-Cyclotetradecatriene-1,3-diol, 1,5,9-trimethyl-12-(1-methylethyl)-	45.678		–	–	–	0.48	0.68	–
62	Cyclooctasiloxane, hexadecamethyl-	45.773		–	0.29	–	–	1.57	1.04
63	7-Hexadecenal, (Z)-	45.855		–	–	1.7	–	–	–
64	β-Elemol	46.067	Sesqui	–	2.33	–	1	–	–
65	Ledol	46.099	Sesqui	–	0.2	0.1	–	0.33	–
66	Ledol	46.367	Sesqui	–	–	0.37	1.22	–	–
67	2,6,10,14-Hexadecatetraen-1-ol, 3,7,11,15-tetramethyl-, acetate, (E,E,E)-	46.409	Mono	0.24	3.66	–	–	–	–
68	d-Ledol	46.431	Sesqui	–	–	–	–	0.51	–
69	Octadecanoic acid	46.54		–	–	0.43	–	–	–
70	Cholest-5-en-3-ol (3.beta.)-, carbonochloridate	46.622		–	–	–	–	–	0.74
71	4,7,10,13,16,19-Docosahexaenoic acid, methyl ester, (all-Z)-	46.62		–	–	–	0.49	–	–
72	Retinol, acetate	46.635		0.32	1.3	–	–	–	–
73	Phytol	46.736	Diter	–	–	–	0.74	0.36	5.11
74	9,12-Octadecadienoic acid, methyl ester, (E,E)-	46.93		–	0.43	–	–	–	–
75	Methyl linolenate	47.109		0.39	1.12	–	1.04	0.4	–
76	9,19-Cyclolanostan-3-ol, acetate, (3.beta.)-	47.27		–	–	0.3	–	–	–
77	cis-Phytol	47.331	Diter	3.19	6.28	6.75	10.84	3.92	–
78	9,12-Octadecadienoic acid (Z,Z)-, methyl ester	47.425		–	–	1.33			–
79	4,8,13-Cyclotetradecatriene-1,3-diol, 1,5,9-trimethyl-12-(1-methylethyl)-	47.773		0.23	1.77	–	1.43	0.51	–
80	11,14,17-Eicosatrienoic acid, methyl ester	47.547		–	–	0.16	–	–	–

81	Propane, 1,2-dibromo-3-chloro-	48.052		−	0.39	−	−	−	−
82	Carveol	48.108	Mono	−	−	−	−	0.27	−
83	Oxirane, dodecyl-	48.412		−	−	0.28	−	−	−
84	α-Linolenic acid	48.479		0.57	14.67	−	9.84	8.15	−
85	α-Limonene diepoxide	48.755	Mono	−	−	−	2.34		−
86	Cyclononasiloxane, octadecamethyl-	48.859		−	−	−	−	2.45	1.02
87	Ledol	48.982	Sesqui	−	−	−	0.28	2.22	−
88	Octadecanoic acid	49.081		−	3.03	−	1.34	−	−
89	Propane, 1,2-dibromo-3-chloro-	49.038		−	−	30.27	−	−	−
90	Heneicosane	49.26		0.99	−	−	−	−	−
91	Triacontane	49.297		−	1.93	−	−	−	−
92	9,12,15-Octadecatrienoic acid, (Z,Z,Z)-	49.406		−	−	5.2	−	−	−
93	Caryophyllene	50.085	Sesqui	30.71	31.53	−	45.45	54.04	72.67
94	Octadecanoic acid	50.255		−	−	0.14	−	−	−
95	Isopulegol	50.305	Mono	−	0.12		−	−	−
96	.alpha.-Guaiene	50.47	Diter	−	−	0.57	−	−	−
97	5.alpha.-Androstan-17-one, 3.alpha.,11.beta.-bis(trimethylsiloxy)-	50.624		−	−	−	0.29	−	−
98	5.beta.,7.beta.H,10.alpha.-Eudesm-11-en-1.alpha.-ol	50.703	Sesqui	−	−	−	−	0.24	−
99	4,8,13-Cyclotetradecatriene-1,3-diol, 1,5,9-trimethyl-12-(1-methylethyl)-	51.152		−	−	0.15	−	0.21	−
100	9,19-Cyclolanostan-3-ol, acetate, (3.beta.)-	51.161		−	0.08		−	−	−
101	Lycopene	51.348	Sesqui	−	−	0.19	0.39	−	−
102	Behenic alcohol	51.489		−	0.24	−	0.32	0.18	−
103	Heneicosane	51.612		3.01	1.56	−	−	−	−
104	Heptasiloxane, hexadecamethyl-	51.881		−	−	−	−	−	1.19
105	Cyclononasiloxane, octadecamethyl-	51.922		−	0.59	−	−	2.32	−
106	Ledol	52.009		−	−	2.89	−	−	−
107	Cyclononasiloxane, octadecamethyl-	52.153		−	−	0.18	−	−	−
108	Spiro[4.5]decane, 6-methylene-	53.001		−	−	−	−	−	0.34
109	11,14,17-Eicosatrienoic acid, methyl ester	53.047		0.1	0.11	−	−	−	−
110	9-Tetradecenal, (Z)-	53.18		−	−	0.14	−	−	−
111	α-Limonene diepoxide	53.194	Mono	−	−	−	−	0.15	−
112	2,6,10,14-Hexadecatetraen-1-ol, 3,7,11,15-tetramethyl-, acetate, (E,E,E)-	53.481	Mono	−	−	−	−	−	−
113	7,10-Hexadecadienoic acid, methyl ester	53.504		−	−	−	−	−	0.77

114	6,9-Octadecadienoic acid, methyl ester	53.532		–	–	–	0.58	–	–
115	α-Elemol	53.591	Sesqui		0.53	–	–	–	–
116	Triadimenol	53.554	Sesqui	–	–	0.2	–	–	–
117	Squalene	53.568	Triter	–	–	–	–	0.48	–
118	4,8,13-Cyclotetradecatriene-1,3-diol, 1,5,9-trimethyl-12-(1-methylethyl)-	53.934		–	–	–	0.26		–
119	Verbenol	53.994	Sesqui	–	–	–	–	0.1	–
120	Caryophyllene oxide	54.241	Sesqui	–	–	0.09	–	–	–
121	Heptadecane	54.464		–	18.37	2954<br>3	–	–	0.44
122	Octacosane	54.55		–	–	–	–	0.11	–
123	Heneicosane	54.539		5.24	1.84	–	–	–	–
124	4,8,13-Cyclotetradecatriene-1,3-diol, 1,5,9-trimethyl-12-(1-methylethyl)-	54.783		–	–	–	0.5	–	–
125	Globulol	54.833	Sesqui	–	–	–	–	0.05	–
126	4,8,13-Cyclotetradecatriene-1,3-diol, 1,5,9-trimethyl-12-(1-methylethyl)-	55.583		–	–	0.08	–		–
127	steviol	55.584	Diter	–	–	–	–	0.03	–
128	9,12,15-Octadecatrienoic acid, ethyl ester, (Z,Z,Z)-	55.863		–	–	–	–	0.06	–
129	9,19-Cyclolanostan-3-ol, acetate, (3.beta.)-	55.931		–	–	–	0.39	–	–
130	Octacosane	56.72		0.09	–	–	–	–	–
131	Heptasiloxane, hexadecamethyl-	56.123		–	–	–	–	–	1.43
132	Cyclononasiloxane, octadecamethyl-	56.186		–	0.52	–	–	1.98	
133	4,8,13-Cyclotetradecatriene-1,3-diol, 1,5,9-trimethyl-12-(1-methylethyl)-	56.308		–	–	2.42	–	–	–
134	8,11,14-Eicosatrienoic acid, (Z,Z,Z)-	56.834		–	0.06	–	–	–	–
135	2,4-Decadienoic acid, ethyl ester, (E,Z)-	56.873		–	–	–	–	0.02	–
136	Cyclononasiloxane, octadecamethyl-	56.953		–	–	0.08	–	–	–
137	Cyclohexanepropanol-	57.471		–	–	–	–	0.02	–
138	9,12,15-Octadecatrienoic acid, ethyl ester, (Z,Z,Z)-	57.659		–	–	–	0.18		–
139	9,12,15-Octadecatrienoic acid, methyl ester, (Z,Z,Z)-	57.744		–	–	–	–	0.04	–
140	Behenic alcohol	58.237		–	–	–	0.63	–	–
141	1-Decanol, 2-hexyl-	58.318		5.82	–	–	–	–	0.63

142	Octacosanol	58.324		–	–	–	–	0.5	–
143	Heneicosane	58.403		–	1.96	–	–	–	–
144	Caryophyllene oxide	58.543	Sesqui	–	–	0.13	–	–	–
145	9,12,15-Octadecatrienoic acid, methyl ester, (Z,Z,Z)-	59.185		–	–	1.44	–	–	–
146	Di-n-octyl phthalate	59.75		–	–	–	0.2	–	–
147	Bis(2-ethylhexyl) phthalate	59.843		0.3	0.13	–	–	0.1	1.03
148	1-Decanol, 2-hexyl-	60.712		–	–	0.57	–	–	–
149	1,2-Cyclohexanedimethanol	60.739		–	–	–	–	0.02	–
150	11,14,17-Eicosatrienoic acid, methyl ester	61.078		–	0.07	–	–	–	–
151	9,12,15-Octadecatrienoic acid, methyl ester, (Z,Z,Z)-	61.14		–	0.07	–	0.11	0.05	–
152	Dotriacontane	61.288		0.21	–	–	–	–	–
153	Pentacosane	61.398		–	0.09	–	–	–	–
154	Octadecane, 3-ethyl-5-(2-ethylbutyl)-	61.831		–	–	–	0.08	–	–
155	Nonacosane	61.88		0.08	0.1	–	–	–	–
156	Pentadecane	61.939		–	–	–	–	0.03	–
157	Bis(2-ethylhexyl) phthalate	61.976		–	–	0.12	–	–	–
158	Heptasiloxane, hexadecamethyl-	62.369		–	–	–	–	–	1.09
159	Cyclononasiloxane, octadecamethyl-	62.455		–	0.43	–	0.08	1.66	–
160	9,12,15-Octadecatrienoic acid, methyl ester, (Z,Z,Z)-	62.609		–	–	2.14	–	–	–
161	Nonacosane	63.277		6.12	–	–	–	–	0.21
162	Cyclononasiloxane, octadecamethyl-	63.292		–	–	0.05	–	–	–
163	Heneicosane	63.41		–	1	–	0.2	0.12	–
164	Nonanoic acid, phenylmethyl ester	64.32		–	–	0.22	–	–	–
165	Hexacosyl acetate	64.094		–	0.03	–	–	–	–
166	1-Cyclohexene-1-methanol	64.573		–	0.01	–	–	–	–
167	Tetracontane	65.08		–	0.05	0.06	–	–	–
168	Tetrapentacontane	66.589		0.3	–	–	–	–	–
169	Heptadecane	67.233			0.04		–	–	–
170	2-Methylbutanoic anhydride	67.279		–	–	–	0.09	0.11	–
171	Dotriacontane	68.746		6.53	–	–	–	–	–
172	Tetracontane	68.85		–	2.59	–	–	–	–
173	Heneicosane	68.884		–	–	–	–	1.04	–
174	Heptasiloxane, hexadecamethyl-	69.7		–	–	–	–	–	1.08

175	Dotriacontane	68.732		–	–	–	–	–	1.83
176	Nonacosane	68.781		–	–	–	2.03	–	–
177	Cyclononasiloxane, octadecamethyl-	69.796		–	0.39	–	0.06	1.48	–
178	Acetic acid n-octadecyl ester	69.939		–	–	5.26	–	–	–
179	Tetrapentacontane	70.772		–	0.03		–	–	–
180	Cyclohexanol, 5-methyl-2-(1-methylethenyl)-	71.092		–	–	–	–	0.01	–
181	4,7-Methano-1H-indene, octahydro-2-(1-methylethylidene)-	72.08		–	–	–	0.1	–	–
182	Benzene, [[(1-ethenyl-1,5-dimethyl-4-hexenyl)oxy]methyl]-	72.107		–	0.15	–	–	–	–
183	Tetrapentacontane	72.2		0.29	0.14	0.06	–	–	–
184	Octadecane, 1-chloro-	72.423		–	–	–	–	0.02	–
185	1,3-Benzenedicarboxylic acid, bis(2-ethylhexyl) ester	72.703		–	–	0.13	–	–	–
186	Oxirane, [(dodecyloxy)methyl]-	72.82		–	–	–	–	–	0.11
187	Octadecane, 3-methyl-	72.917		–	0.08	–	0.13	–	–
188	7-Hexadecenal, (Z)-	72.997		–	–	–	–	0.04	–
189	Tetracosane	73.344		–	–	0.32	–	–	–
190	Behenic alcohol	73.465		0.05	–	–	–	–	–
191	2H-Pyran-2-one, 6-heptyltetrahydro-	73.513		–	0.15	–	–	–	–
192	Nonacosane	74.603		–	0.56	–	0.42	–	–
193	Benzene, [[(1-ethenyl-1,5-dimethyl-4-hexenyl)oxy]methyl]-	74.1		–	–	0.31	–	–	–
194	Dotriacontane	74.491		5.48	–	–	–	–	–
195	Squalene	74.571	triter	–	–	–	–	–	1.24
196	Nonacosane	74.661		–	–	–	–	0.16	
197	Cyclononasiloxane, octadecamethyl-	75.784		–	–	0.36	–	–	–
198	Octadecane, 3-ethyl-5-(2-ethylbutyl)-	76.289		–	0.01	–	–	–	–
199	Cyclononasiloxane, octadecamethyl-	77.347		–	–	–	–	–	0.94
200	Cyclononasiloxane, octadecamethyl-	77.453		–	0.34	–	–	1.37	–
201	Nonacosane	77.605		–	0.76	2.27	–	–	–
202	Tetrapentacontane	78.141		1.47	–	–	–	–	2.54
203	Tetracontane	78.201		–	–	–	1.09	–	–
204	Nonacosane	78.318		–	0.02	–	–	0.4	–
205	Cyclononasiloxane, octadecamethyl-	79.505		–	–	0.63	–	–	–
206	Octadecane, 3-methyl-	72.917		–	0.08	–	–	–	–

Table S11. List of *S. officinalis* primer pairs used for cloning of full-length terpene synthase genes

**Gene ID from RNA-Seq**	**Gene name**	**Primer name**	**Short Primer Sequence**	**Long Primer Sequence**	**Size (bp)**
DSo-comp10962_c0	*SoNEOD*	PCR- NEOD -F	ATGGCAGATGCACTTGTCCAG	GGGGACAAGTTTGTACAAAAAAGCAGGCTTCATGGCAGATGCACTTGTC	1072
		PCR- NEOD -R	TTACAAATCACACAATTATTAGGAGGAG	GGGGACCACTTTGTACAAGAAAGCTGGGTCACAATTATTAGGAGGAG	
DSo-comp26990_c0	*SoCINS*	PCR-CINS-F	ATGTCGAGTCTTATAATGCAAGTTGTG	GGGGACAAGTTTGTACAAAAAAGCAGGCTTC ATGTCGAGTCTTATAATG	1776
		PCR-CINS-R	TCATAGCGGTGGAACAGCAAG	GGGGACCACTTTGTACAAGAAAGCTGGGTTCATAGCGGTGGAACAGC	
DSo-comp18462_c0	*SoSABS*	PCR-SABS-F	GAATTCTCAGACAACATGGTTTTG	GGGGACAAGTTTGTACAAAAAAGCAGGCTTC GAATTCTCAGACAACATG	1182
		PCR-SABS-R	GTTCATCTCCTTCCACGCCT	GGGGACCACTTTGTACAAGAAAGCTGGGTGTTCATCTCCTTCCACGC	
DSo-comp6814_c0	*SoLINS*	PCR- LINS -F	AGAGATATGTTGATGAAAATGGAGC	GGGGACAAGTTTGTACAAAAAAGCAGGCTTC AGAGATATGTTGATGA	1520

		PCR- LINS -R	CCTAGGAGTGATTTGGCGAAG	GGGGACCACTTTGTACAAGAAAGCTGGGTC CTAGGAGTGATTTGG	
DSo-comp26367_c0	*SoTPS6*	PCR- TPS6 -F	GATGAAGATGATTCAACCCCA A	GGGGACAAGTTTGTACAAAAAAGCAGGCTT C GATGAAGATGATTCAACC	1464
		PCR- TPS6 -R	CTAGCTAGAAAGCATGAAGG GG	GGGGACCACTTTGTACAAGAAAGCTGGGTCT AGCTAGAAAGCATGAA	

Figure S1

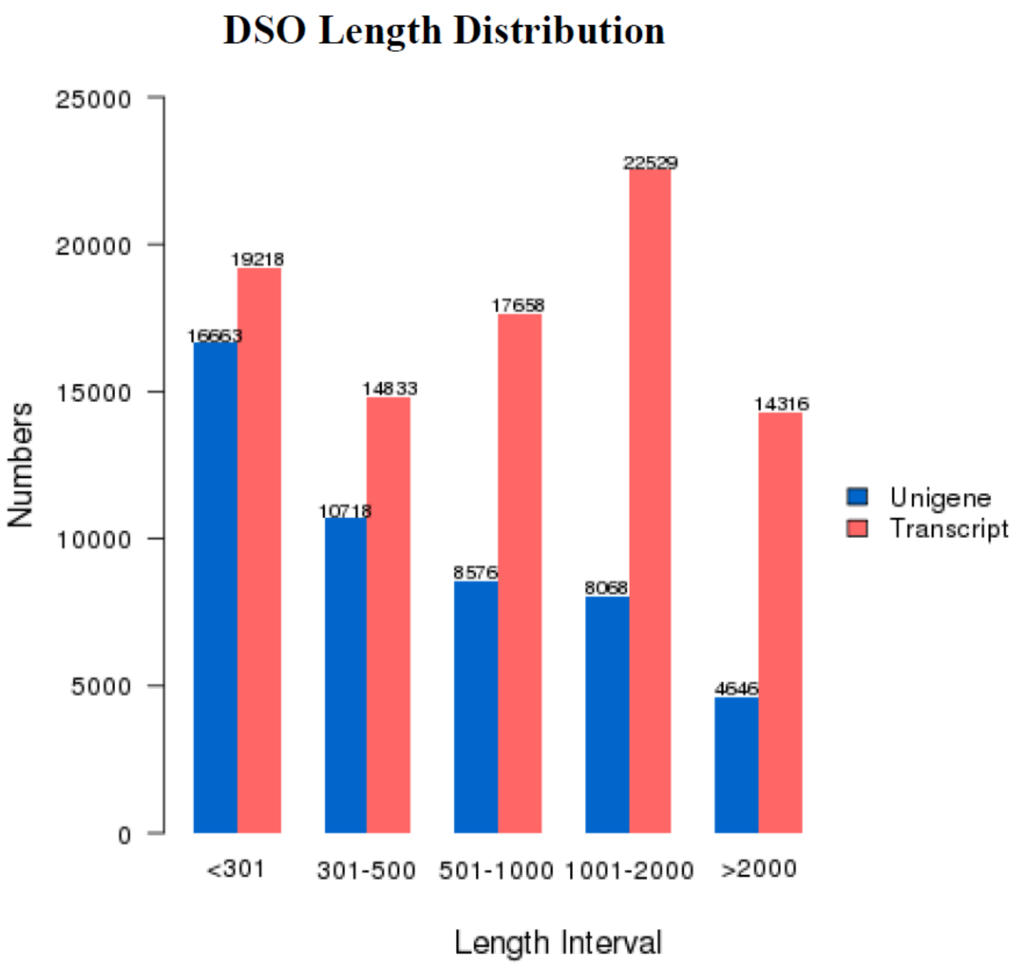
DSO Length Distribution
25000
20000
15000
10000
5000
0
Numbers
19218
16663
14833
10718
17658
8576
22529
8068
14316
4646
<301
301-500
501-1000
1001-2000
>2000
Length Interval
Unigene
Transcript

Figure S2

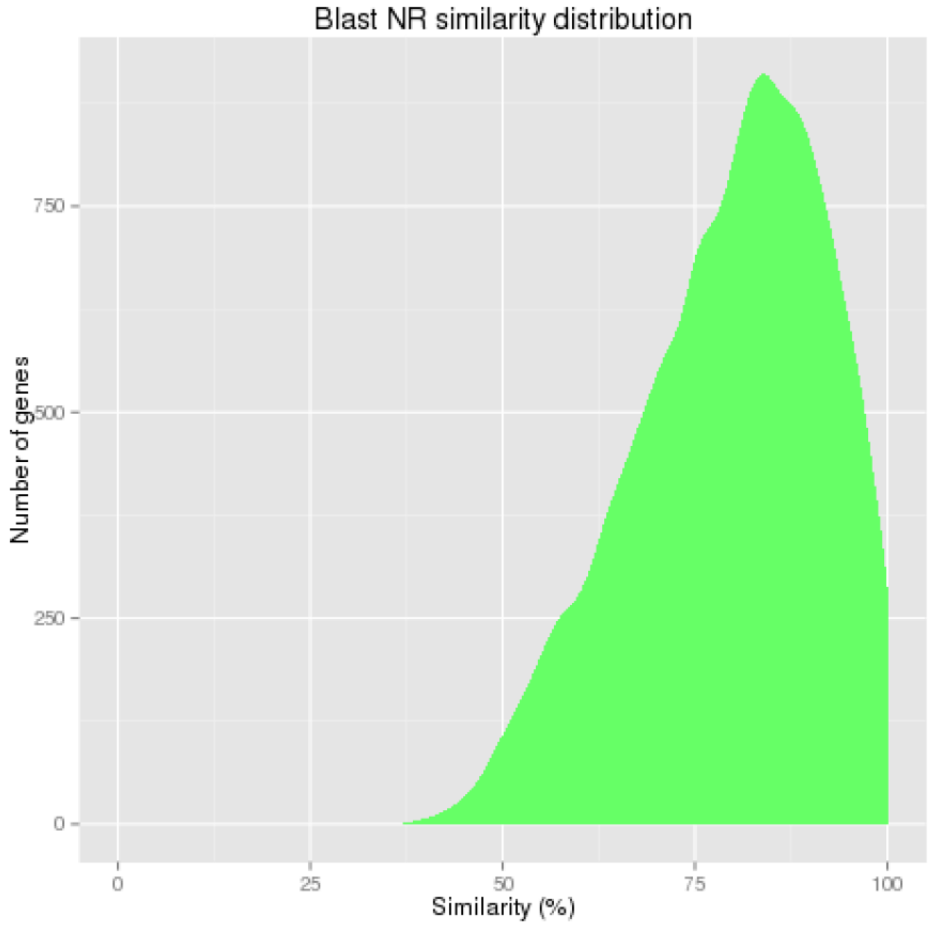

Blast NR similarity distribution
750
500
250
0
Number of genes
0
25
50
75
100
Similarity (%)

Figure S3

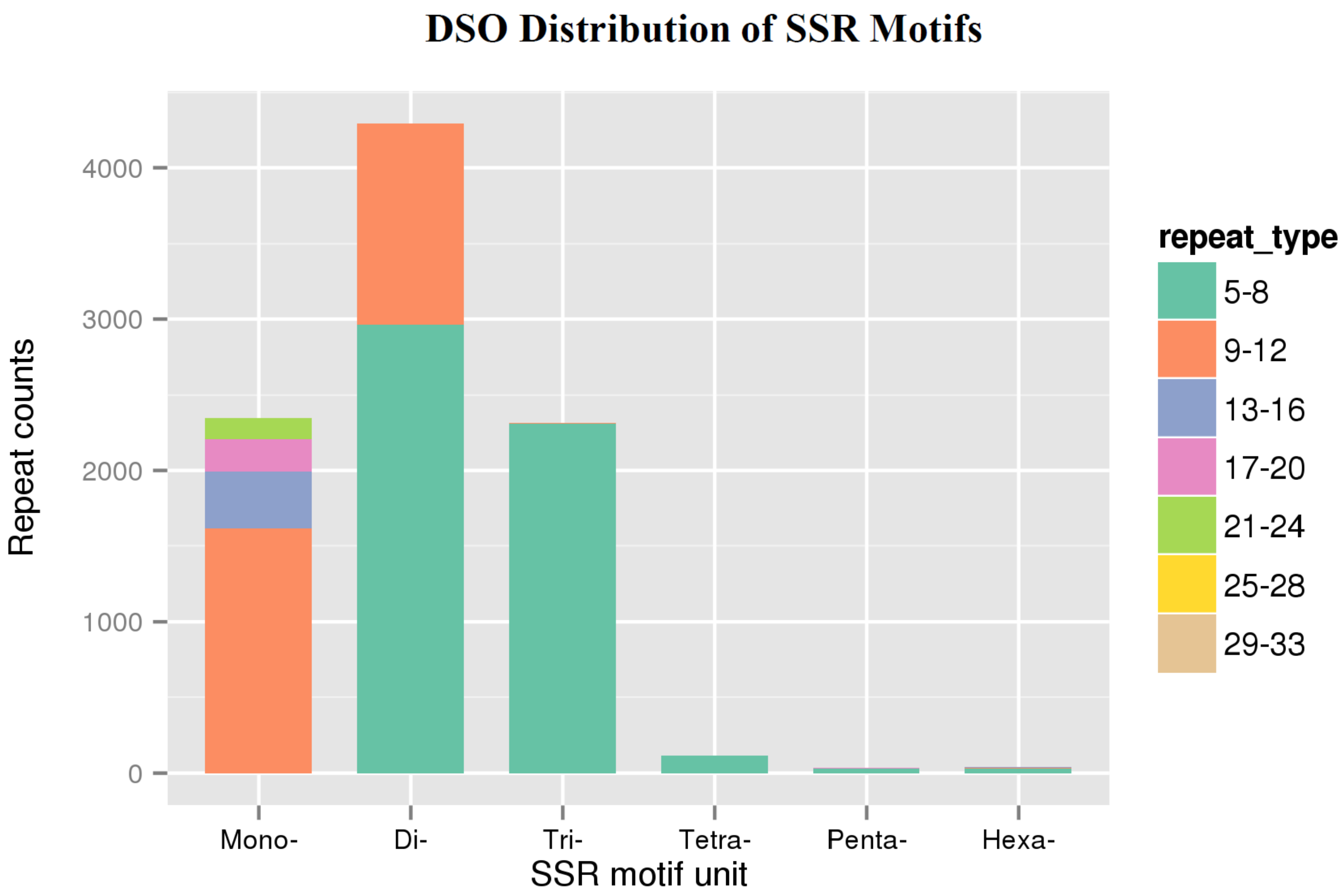
DSO Distribution of SSR Motifs
Repeat counts
4000
3000
2000
1000
0
Mono-
Di-
Tri-
Tetra-
Penta-
Hexa-
SSR motif unit
repeat_type
5-8
9-12
13-16
17-20
21-24
25-28
29-33

Figure S4.

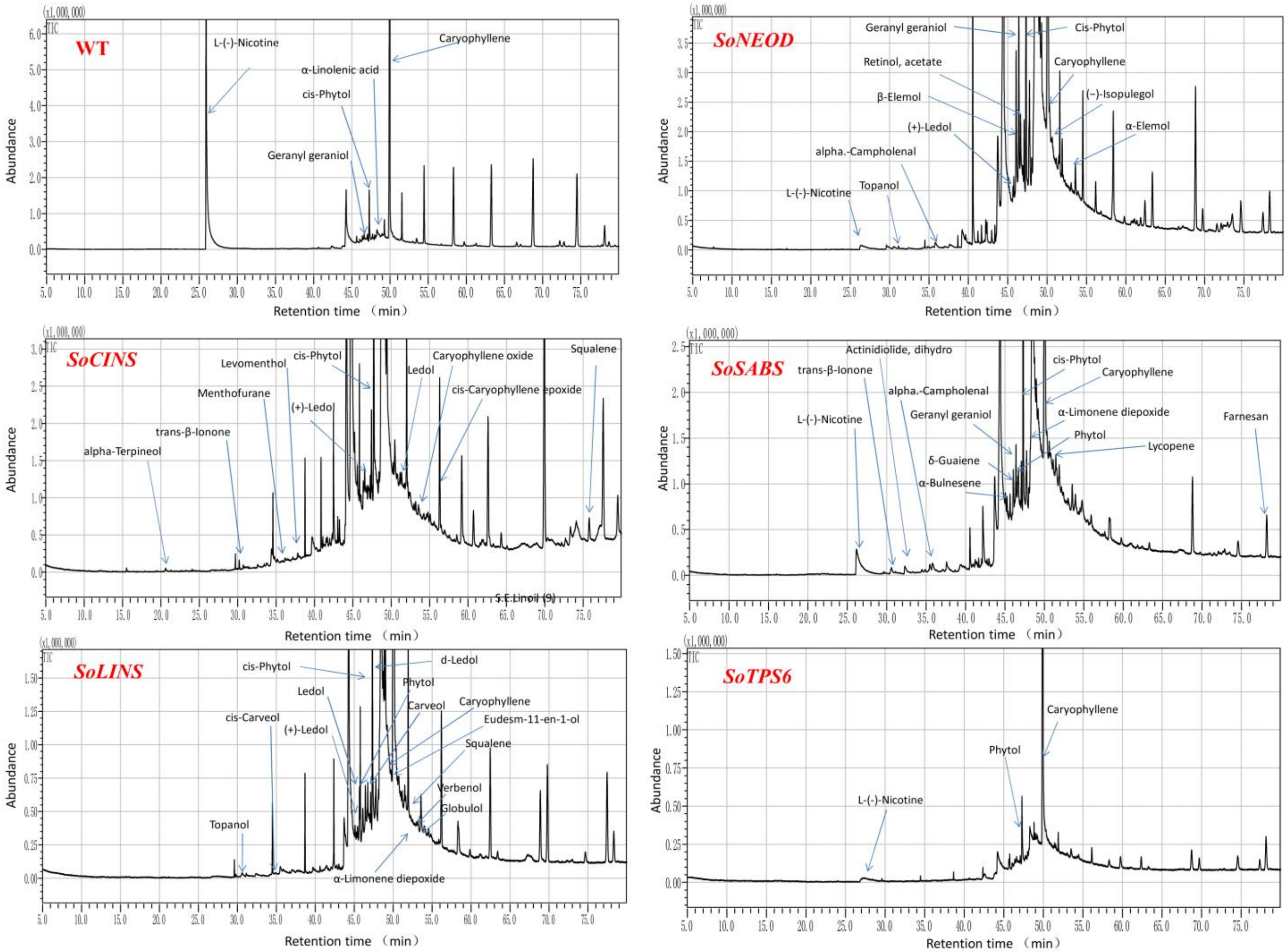
WT
L-(-)-Nicotine
Caryophyllene
α-Linolenic acid
cis-Phytol
Geranyl geraniol
SoNEOD
Geranyl geraniol
Cis-Phytol
Retinol, acetate
Caryophyllene
β-Elemol
(−)-Isopulegol
(+)-Ledol
α-Elemol
alpha.-Campholenal
Topanol
L-(-)-Nicotine
SoCINS
Levomenthol
cis-Phytol
Caryophyllene oxide
Squalene
Ledol
Menthofurane
cis-Caryophyllene epoxide
(+)-Ledol
trans-β-Ionone
alpha-Terpineol
SoSABS
Actinidiolide, dihydro
trans-β-Ionone
cis-Phytol
Caryophyllene
alpha.-Campholenal
α-Limonene diepoxide
L-(-)-Nicotine
Geranyl geraniol
Farnesan
Phytol
Lycopene
δ-Guaiene
α-Bulnesene
SoLINS
cis-Phytol
d-Ledol
Ledol
Phytol
cis-Carveol
Carveol
Caryophyllene
(+)-Ledol
Eudesm-11-en-1-ol
Squalene
Verbenol
Globulol
Topanol
α-Limonene diepoxide
SoTPS6
Caryophyllene
Phytol
L-(-)-Nicotine
Abundance
Retention time （min）
